# Supplementary material for: Self-assembly of emissive supramolecular rosettes with increasing complexity using multitopic terpyridine ligands
Source: Nat Commun. 2018 Feb 8;9:567. doi: 10.1038/s41467-018-02959-w (PMC5805703; doi:10.1038/s41467-018-02959-w)
Supplement: Supplementary file 1 — Supplementary Information [file 41467_2018_2959_MOESM1_ESM.pdf]

Self-Assembly of Emissive Supramolecular Rosettes with Increasing Complexity Using Multitopic Terpyridine Ligands

Guang-Qiang Yin, [1, 2, +] Heng Wang, [2, +] Xu-Qing Wang, [1] Bo Song, [2] Li-Jun Chen, [1] Lei Wang, [2] Xin-Qi Hao, [3] Hai-Bo Yang, [1, *] Xiaopeng Li[2, *]

[1]Shanghai Key Laboratory of Green Chemistry and Chemical Processes, Zhuang Chang Gong Institute, School of Chemistry and Molecular Engineering, East China Normal University, 3663 North Zhongshan Road, Shanghai 200062, P. R. China

[2]Department of Chemistry, University of South Florida, Tampa 33620, United States

[3]College of Chemistry and Molecular Engineering, Zhengzhou University, Zhengzhou 450001, P. R. China

[+] These authors contributed equally to this work.

*Correspondence and requests for materials should be addressed to

E-mail: xiaopengli1@usf.edu (X. L.); hbyang@chem.ecnu.edu.cn (H.-B.Y.)

Supplementary Methods

General Procedures. All reagents were purchased from Sigma-Aldrich, Matrix Scientific, Alfa Aesar, and used without further purification. 2-Bromo-1,1,2-triphenylethylene was purchased from TCL company. Compound **1**,[1] 4'-(4-boronophenyl)-2,2':6',2"-terpyridine (**2**),[2] Compound **3**,[3] and Compound **5**[4] were synthesized according to the literature procedures. Column chromatography was conducted using SiO_2 (VWR, 40–60 µm, 60 Å) and the separated products were visualized by UV light. NMR spectra data were recorded on a 400 MHz and 500 MHz Varian NMR spectrometer in $CDCl_3$, CD_3CN and DMSO-d_6 with TMS as reference. ESI-MS and TWIM-MS were recorded with a Waters Synapt G2 mass spectrometer, using solutions of 0.01 mg sample in 1 mL of $CHCl_3/CH_3OH$ (1:3, v/v) for ligands or 0.5 mg sample in 1 mL of MeCN/MeOH (3:1, v/v) for supramolecules. Electronic absorption and fluorescence experiments were conducted on a HORIBA FLUOROMAX-4C-L. The experimental quantum yields were determined by integrating sphere. Dynamic light scattering (DLS) was carried out on a Nano-ZS90 instrument at room temperature. Time-resolved fluorescence decay experiments were conducted with FLS 980 instrument (excited at 375 nm). The association constants of model compound **16** binding with $Cd(NO_3)_2$ in various solvents were calculated using BINDFIT[5-6] based on 2:1 binding model. The equation used for these analyses is available in the review by Thordarson.[7]

TWIM-MS. We used the following conditions to perform TWIM-MS experiments: sample cone voltage, 30 V; extraction cone voltage, 3.0 V; ESI capillary voltage, 3 kV; source temperature, 100 ℃; desolvation temperature, 100 ℃; cone gas flow, 10 L/h; desolvation gas flow, 700 L/h (N_2); source gas control, 0 mL/min; trap gas control, 2 mL/min; helium cell gas control, 100 mL/min; ion mobility (IM) cell gas control, 30 mL/min; sample flow rate, 5 µL/min; traveling wave height, 25 V; and traveling wave velocity, 1000 m/s.

Collision cross-section calibration. The calibration procedure of Scrivens *et al*[8] was used to convert the drift time scale of the TWIM-MS experiments to a collision

cross-section (CCS) scale. The calibration curve was constructed by plotting the corrected CCSs of the molecular ions of myoglobin against the corrected drift times of the corresponding molecular ions measured in TWIM-MS experiments at the same traveling wave velocity, traveling wave height and ion mobility gas flow settings *viz.*, 1000 m/s, 25 V, and 30 mL/min, respectively.

Molecular modeling. Energy minimization of the supramolecular rosettes was performed with Materials Studio version 4.2, using the Anneal and Geometry Optimization tasks in the Forcite module (Accelrys Software, Inc.). All counterions were omitted. An initially energy-minimized structure was subjected to 70 annealing cycles with initial and mid-cycle temperatures of 50 and 1400 K, respectively, twenty heating ramps per cycle, one thousand dynamic steps per ramp, and one femtosecond per dynamic step. A constant volume/constant energy (NVE) ensemble was used and the geometry was optimized after each cycle. Geometry optimization used a universal force field with atom-based summation and cubic spline truncation for both the electrostatic and Van der Waals parameters. Energy-minimized structures were used for the calculation of theoretical collision cross-sections using MOBCAL programs.[8-10]

TEM. The sample mixtures (10^{-6} M) were drop-casted on to a lacey carbon covered Cu grid (300 mesh, purchased from Ted Pella Inc.) or carbon-coated Cu grid (400 mesh, purchased from SPI supplies), and the extra solution was absorbed by filter paper to avoid further aggregation. The TEM images were taken with a FEI Morgagni transmission electron microscope.

AFM. AFM imaging was carried out with a Digital Instrument Nanoscope Dimension 3000 system. The sample was diluted to a concentration of 10^{-6} M using acetonitrile, dropped on freshly cleaved mica surface, and then dried in the air. Silicon cantilevers tip with spring constant of around 0.1 N/m was used for the experiments.

Synthesis of ligands L1, L2, and L3

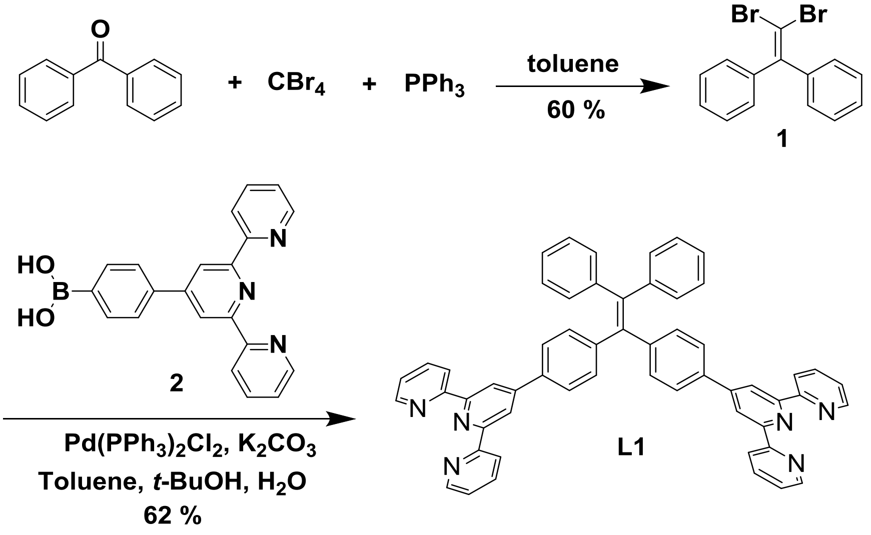

Supplementary Figure 1: Synthesis of ligand **L1**

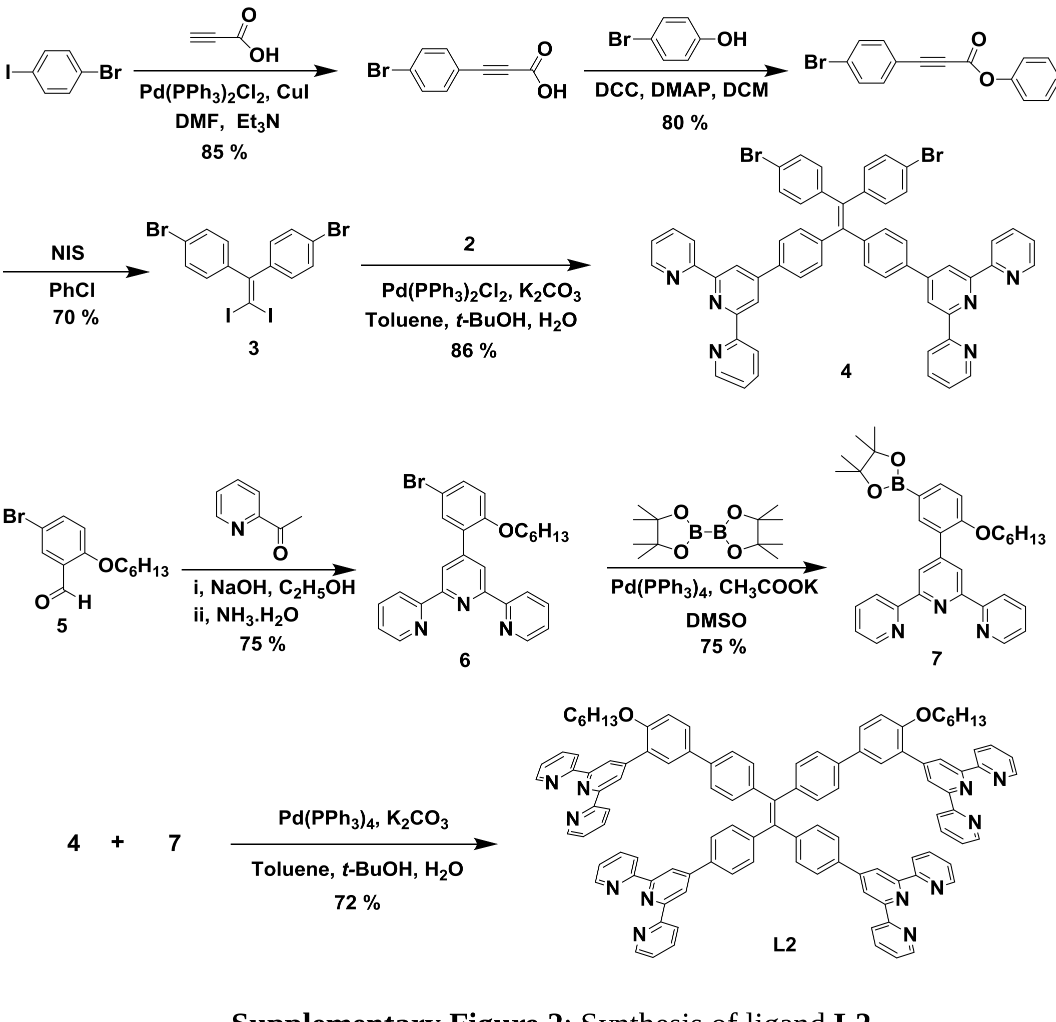

Supplementary Figure 2: Synthesis of ligand **L2**

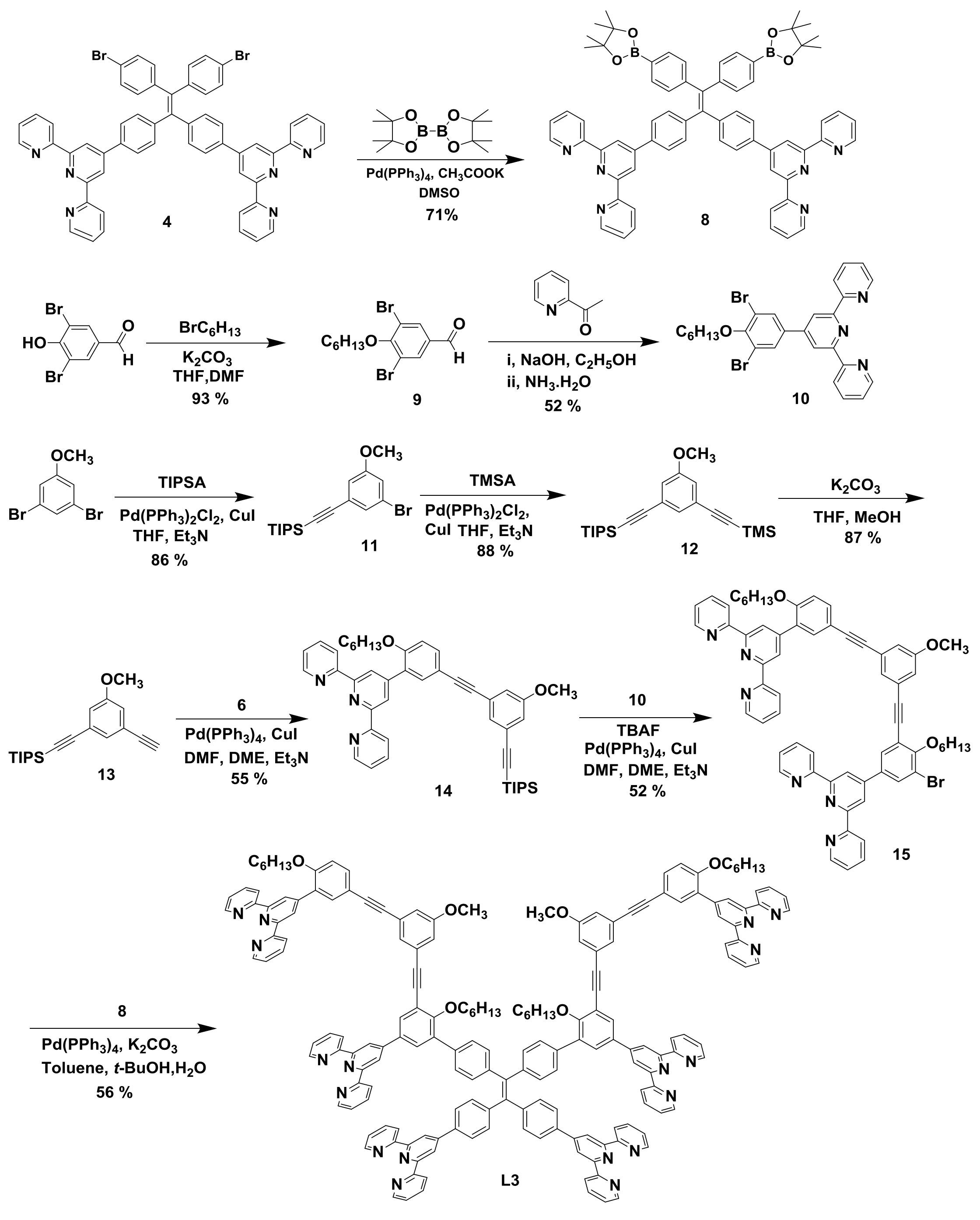

Supplementary Figure 3: Synthesis of ligand **L3**

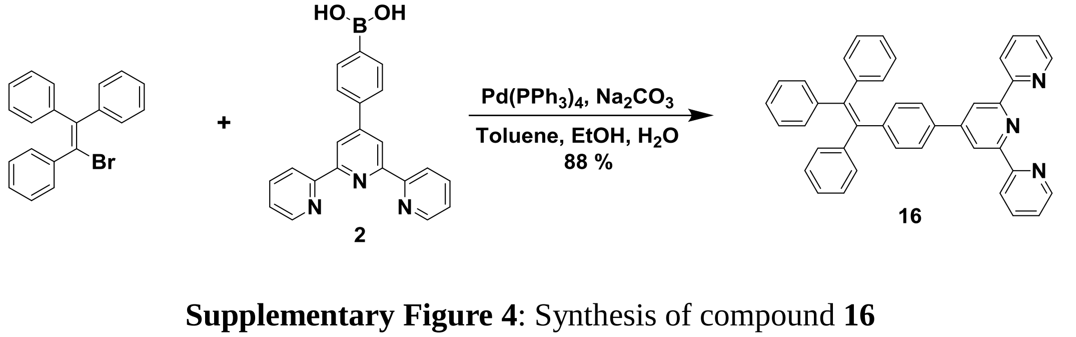

Supplementary Figure 4: Synthesis of compound **16**

Preparation of Ligands and Supramolecular Architectures.

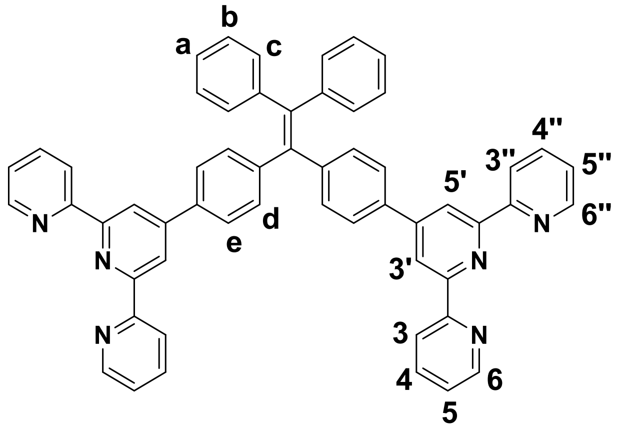

Ligand L1: To a Schlenk flask containing compound **1** (500 mg, 1.49 mmol), compound **2** (1.05 g, 2.98 mmol), $Pd(PPh_3)_2Cl_2$ (63 mg, 0.09 mmol) and K_2CO_3 (1.2 g, 9.0 mmol) were added. After the removal of air and back-filled with nitrogen, 15 mL of toluene, 15 mL of water and 5 mL of *t*-butanol were added. After stirring at 85 ℃ for 12 h, the mixture was cooled to 25 ℃. The aqueous layer was extracted with CH_2Cl_2, and then the combined organic phase was washed with brine and dried with $MgSO_4$. The solvents were then removed under reduced pressure. The residue was purified by silica gel chromatography, eluting with chloroform to afford compound **L1** as white solid (732 mg, 62%). ^1H NMR (500 MHz, $CDCl_3$) δ 8.72 (dd, J = 4.7, 0.7 Hz, 4H, tpy-$H^{6,6''}$), 8.70 (s, 4H, tpy-$H^{3',5'}$), 8.66 (d, J = 7.9 Hz, 4H, tpy-$H^{3,3''}$), 7.87 (td, J = 7.7, 1.8 Hz, 4H, tpy-$H^{4,4''}$), 7.69 (d, J = 8.3 Hz, 4H, Ph-H^{e}), 7.34 (ddd, J = 7.4, 4.8, 1.1 Hz, 4H, tpy-$H^{5,5''}$), 7.20 (d, J = 8.3 Hz, 4H, Ph-H^{d}), 7.16 (d, J = 1.6 Hz, 2H, Ph-H^{a}), 7.15 (d, J = 1.8 Hz, 4H, Ph-H^{c}), 7.13 – 7.09 (m, 4H, Ph-H^{b}). ^{13}C NMR (125 MHz, $CDCl_3$) δ 156.26, 155.83, 149.95, 149.09, 144.48, 143.41, 142.22, 139.62, 136.82, 136.43, 131.97, 131.32, 127.87, 126.81, 126.71, 123.76, 121.30, 118.74. ESI-TOF (*m/z*): Calcd. For $[C_{56}H_{39}N_6]^+$: 795.32, Found for $[M+H]^+$: 795.32. MALDI-TOF MS (*m/z*): Calcd. for $[C_{56}H_{38}N_6]^+$: 794.32, Found $[M•]^+$ (radical): 794.52.

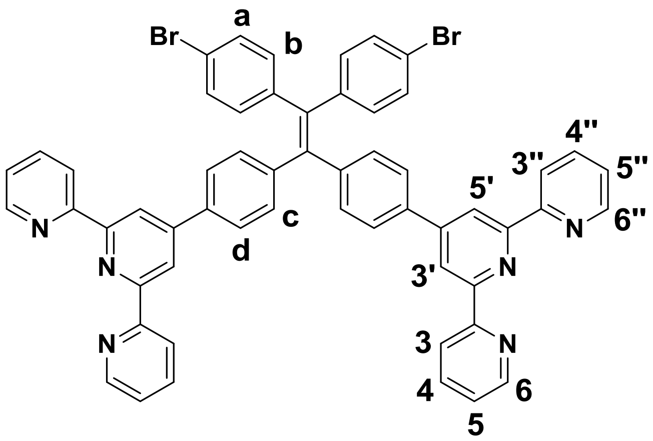

Compound 4: To a Schlenk flask containing compound **3** (1.0 g, 1.7 mmol), compound **2** (1.2 g, 3.4 mmol), $Pd(PPh_3)_2Cl_2$ (70.0 mg, 0.10 mmol) and K_2CO_3 (1.4 g, 10.2 mmol) were added. After the removal of air and back-filled with argon, 30 mL of toluene, 30 mL of water and 10 mL of *t*-butanol were added. After stirring at 80 ℃ for 12 h, the mixture was cooled to 25 ℃. The aqueous layer was extracted with CH_2Cl_2, and then the combined organic phase was washed with brine and dried with $MgSO_4$. The solvents were then removed under reduced pressure. The residue was purified by silica gel chromatography, eluting with chloroform to afford compound **2** as light yellow solid (1.4 g, 86%). ^{1}H NMR (400 MHz, $CDCl_3$) δ 8.72 (m, 8H, tpy-$H^{6,6''}$ and tpy-$H^{3',5'}$), 8.67 (d, J = 8.0 Hz, 4H, tpy-$H^{3,3''}$), 7.88 (t, J = 7.7 Hz, 4H, tpy-$H^{4,4''}$), 7.72 (d, J = 8.3 Hz, 4H, Ph-H^{d}), 7.37 – 7.33 (m, 4H, tpy-$H^{5,5''}$), 7.30 (d, J = 8.4 Hz, 4H, Ph-H^{a}), 7.18 (d, J = 8.2 Hz, 4H, Ph-H^{c}), 6.96 (d, J = 8.4 Hz, 4H, Ph-H^{b}). ^{13}C NMR (100 MHz, $CDCl_3$) δ 156.18 ,155.88, 149.71,149.08,143.71,141.88, 140.85, 139.52, 137.00, 136.82, 132.91, 131.79, 131.24, 126.98, 123.78, 121.29, 121.16, 118.75. ESI-TOF (*m/z*): Calcd. for $[C_{56}H_{37}Br_2N_6]^+$ and $[C_{56}H_{38}Br_2N_6]^{2+}$: 951.14 and 476.08, Found for $[M+H]^+$ and $[M+2H]^{2+}$: 951.23 and 476.09.

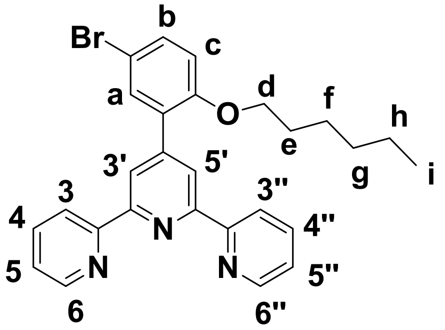

Compound 6: To a solution of NaOH powder (3.2 g, 80 mmol) in 70 mL of EtOH, **5** (4.3 g, 15 mmol) and 2-acetylpyridine (4.0 g, 33 mmol) were added. After stirring at 25 ℃ for 10 h, aqueous $NH_3 \cdot H_2O$ (50 mL) was added and the mixture was refluxed for 20 h. After cooling to room temperature, ethanol was removed under reduced pressure. The aqueous phase was extracted using DCM and the organic layer was washed with water for three times. After the solvent was removed under reduced pressure, the residue was purified by silica gel chromatography with DCM as eluent to afford compound **6** as white solid (5.5 g, 75%). ^{1}H NMR (400 MHz, $CDCl_3$) δ 8.69 (dt, *J* = 4.7, 2.3 Hz, 2H, tpy-$H^{6,6''}$), 8.66 (d, *J* = 6.7 Hz, 2H, tpy-$H^{3,3''}$), 8.65 (s, 2H, tpy-$H^{3',5'}$), 7.94 – 7.83 (m, 2H, tpy-$H^{4,4''}$), 7.67 (d, *J* = 2.5 Hz, 1H, Ph-H^a), 7.51 – 7.41 (m, 1H, Ph-H^b), 7.33 (ddd, *J* = 7.4, 4.8, 1.1 Hz, 2H, tpy-$H^{5,5''}$), 6.92 – 6.83 (m, 1H, Ph-H^c), 3.98 (t, *J* = 6.3 Hz, 2H, Alkyl-H^d), 1.68 (dd, *J* = 10.3, 4.1 Hz, 2H, Alkyl-H^e), 1.36 (dd, *J* = 15.3, 7.6 Hz, 2H, Alkyl-H^f), 1.19 – 1.08 (m, 4H, Alkyl-H^g and Alkyl-H^h), 0.72 (t, *J* = 7.1 Hz, 3H, Alkyl-H^i). ^{13}C NMR (100 MHz, $CDCl_3$) δ 156.12, 155.36, 155.08, 148.94, 146.82, 136.58, 132.76, 132.29, 130.13, 123.51, 121.46, 121.03, 113.83, 112.70, 68.68, 31.36, 28.91, 25.61, 22.21, 13.79. ESI-TOF (*m/z*): Calcd. for $[C_{27}H_{27}BrN_3O]^+$: 488.13, Found for $[M+H]^+$: 488.14.

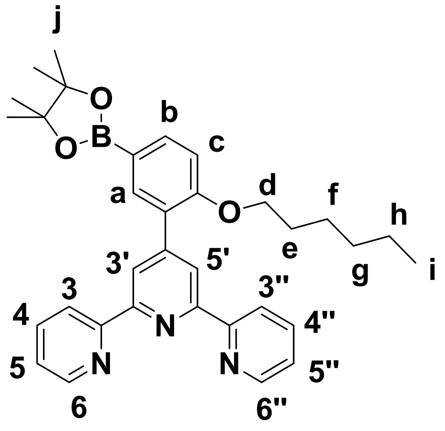

Compound 7: A Schlenk flask was charged with compound **6** (500 mg, 1.03 mmol), *bis*(pinacolato)diboron (308 mg, 1.21 mmol), $Pd(PPh_3)_4$ (35 mg, 0.03 mmol) and KOAc (294 mg, 3.0 mmol) under argon. Dry DMSO (15 mL) was then added, and the suspension was stirred at 80 ℃ for 15 h. 50 mL of brine was added and DCM was used to extract for three times. After that, the organic layer was washed for three times with water. DCM was removed and the residue was purified by silica gel chromatography with DCM as eluent to afford compound **7** as yellowish solid (360 mg, 75%). ^{1}H NMR (400 MHz, $CDCl_3$) δ 8.72 (d, J = 7.5 Hz, 2H, tpy-$H^{6,6''}$), 8.69 (d, J = 8.3 Hz, 2H, tpy-$H^{3,3''}$), 8.67 (s, 2H, tpy-$H^{3',5'}$), 7.90 (t, J = 7.4 Hz, 2H, tpy-$H^{4,4''}$), 7.83 (dd, J = 8.3, 1.3 Hz, 1H, Ph-H^{a}), 7.67 (dd, J = 12.0, 7.1 Hz, 1H, Ph-H^{b}), 7.39 – 7.32 (m, 2H, tpy-$H^{5,5''}$), 6.99 (d, J = 8.3 Hz, 1H, Ph-H^{c}), 4.04 (t, J = 6.3 Hz, 2H, Alkyl-H^{d}), 1.72 (d, J = 2.8 Hz, 2H, Alkyl-H^{e}), 1.44 – 1.38 (m, 2H, Alkyl-H^{f}), 1.26 (s, 12H, H^{j}), 1.18 – 1.09 (m, 4H, Alkyl-H^{g} and Alkyl-H^{h}), 0.72 (t, J = 7.0 Hz, 3H, Alkyl-H^{i}). ^{13}C NMR (125 MHz, $CDCl_3$) δ 158.80, 156.53, 154.88, 148.97, 148.46, 137.15, 137.04, 136.64, 127.78, 123.43, 121.99, 121.19, 111.24, 83.61, 68.27, 31.46, 29.01, 25.71, 24.82, 24.50, 22.29, 13.87. ESI-TOF (*m/z*): Calcd. for $[C_{33}H_{39}BN_3O_3]^+$: 536.31, Found for $[M+H]^+$: 536.34.

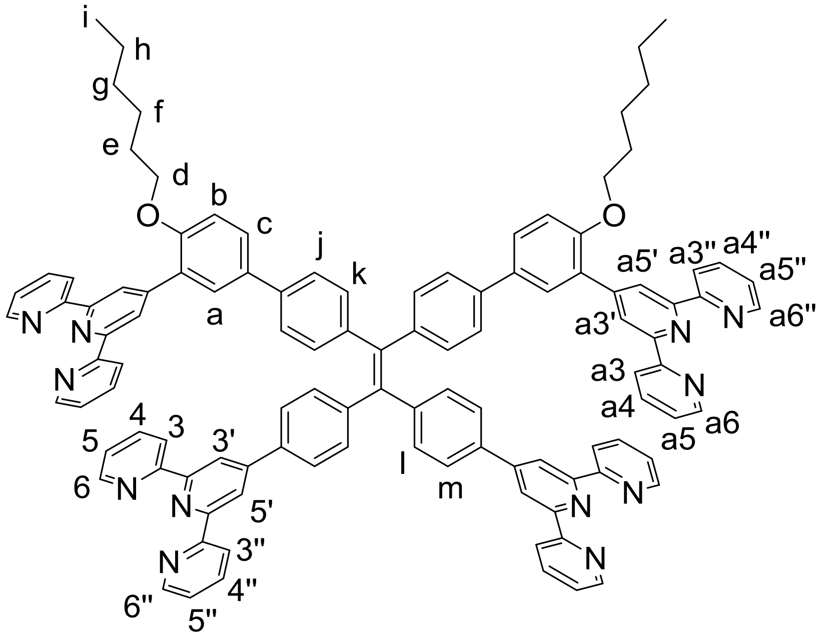

Ligand L2: Under argon, a mixture of compound **4** (200 mg, 0.21 mmol), compound **7** (225 mg, 0.42 mmol), $Pd(PPh_3)_4$ (12 mg, 0.01 mmol), and K_2CO_3 (174 mg, 1.26 mmol), in 15 mL of toluene , 15 mL of water and 5 mL of *t*-butanol was stirred at 85 ℃ for 24 h. 100 mL of water was added and DCM was used to extract for three times. After that, the organic layer was washed for three times with water. DCM was removed and the residue was purified by silica gel chromatography with chloroform (with 2% ethanol) as eluent to afford **L2** in 72% yield as a yellowish solid. ^{1}H NMR (500 MHz, $CDCl_3$) δ 8.72 (s, 4H, tpy-$H^{3',5'}$), 8.71 (s, 4H, tpy-$H^{a3',a5'}$), 8.68 (d, J = 4.4 Hz, 4H, tpy-$H^{6,6''}$), 8.64 (s, 4H, tpy-$H^{a6,a6''}$), 8.64 – 8.59 (m, 8H, tpy-$H^{3,3''}$ and tpy-$H^{a3,a3''}$), 7.86 (d, J = 8.0 Hz, 4H, tpy-$H^{4,4''}$), 7.82 (d, J = 8.6 Hz, 4H, tpy-$H^{a4,a4''}$), 7.78 (s, 2H, Ph-H^{a}), 7.75 (d, J = 8.1 Hz, 4H, Ph-H^{m}), 7.61 (d, J = 7.4 Hz, 2H Ph-H^{c}), 7.46 (d, J = 8.1 Hz, 4H, Ph-H^{j}), 7.31 (m, 8H, tpy-$H^{5,5''}$, tpy-$H^{a5,a5''}$), 7.28 (d, J = 3.6 Hz, 4H, Ph-H^{l}), 7.21 (d, J = 8.0 Hz, 4H, Ph-H^{k}), 7.03 (d, J = 8.6 Hz, 2H, Ph-H^{b}), 4.03 (t, J = 6.1 Hz, 4H, Alkyl-H^{d}), 1.75 – 1.67 (m, 4H, Alkyl-H^{e}), 1.43 – 1.35 (m, 4H, Alkyl-H^{f}), 1.17 (dd, J = 10.1, 5.2 Hz, 4H, Alkyl-H^{g}), 1.11 (dd, J = 14.4, 7.1 Hz, 4H, Alkyl-H^{h}), 0.72 (t, J = 7.0 Hz, 6H, Alkyl-H^{i}). ^{13}C NMR (125 MHz, $CDCl_3$) δ 156.54, 156.30, 155.80, 155.04, 149.97, 149.32, 149.09, 149.05, 148.46, 144.77, 142.11, 141.55, 139.45, 138.64, 136.73, 136.59, 136.42, 133.29, 132.08, 131.96, 129.08, 128.58, 128.39, 126.88, 126.20, 123.66, 123.42, 121.96, 121.24, 121.14, 118.84,

112.54, 77.25, 77.00, 76.75, 68.64, 31.53, 29.15, 25.78, 22.35, 13.92. ESI-TOF (*m/z*): Calcd. for, $[C_{110}H_{90}N_{12}O_2]^+$, $[C_{110}H_{91}N_{12}O_2]^{2+}$: 1609.72, 805.37. Found for $[M+H]^+$ and $[M+2H]^{2+}$: 1609.41, 805.38. MALDI-TOF MS (*m/z*): Calcd. for $[C_{110}H_{89}N_{12}O_2]^+$: 1609.72, Found: 1609.06.

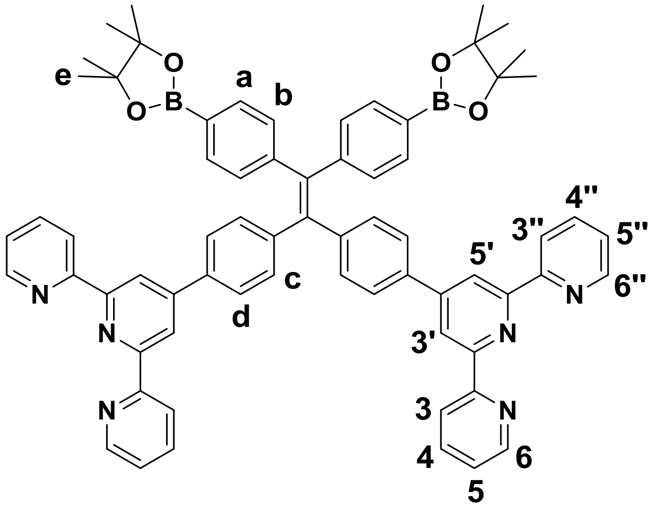

Compound 8: A Schlenk flask was charged with compound **4** (1.0 g, 1.05 mmol), *bis*(pinacolato)diboron (534 mg, 2.1 mmol), $Pd(PPh_3)_4$ (70 mg, 0.06 mmol) and KOAc (617 mg, 6.3 mmol) under argon. Dry DMSO (25 mL) was then added, and the suspension was stirred at 80 ℃ for 15 h. 80 mL of brine was added and DCM was used to extract for three times. After that, the organic layer was washed for three times with water. DCM was removed and the residue was purified by silica gel chromatography with chloroform (with 1% ethanol) as eluent to afford compound **8** as yellowish solid (780 mg, 71%). ^{1}H NMR (400 MHz, $CDCl_3$) δ 8.77 – 8.68 (m, 8H, tpy-$H^{3',5'}$ and tpy-$H^{6,6''}$), 8.66 (d, *J* = 8.0 Hz, 4H, tpy-$H^{3,3''}$), 7.87 (t, *J* = 7.6 Hz, 4H, tpy-$H^{4,4''}$), 7.69 (d, *J* = 7.1 Hz, 4H, Ph-H^{d}), 7.58 (d, *J* = 7.0 Hz, 4H, Ph-H^{a}), 7.33 (dd, *J* = 11.1, 5.1 Hz, 4H, tpy-$H^{5,5''}$), 7.20 (d, *J* = 7.6 Hz, 4H, Ph-H^{c}), 7.09 (d, *J* = 7.0 Hz, 4H, Ph-H^{b}), 1.40 – 1.27 (m, 24H, H^{e}). ^{13}C NMR (125 MHz, $CDCl_3$) δ 156.24, 155.80, 149.87, 149.08, 146.27, 144.28, 141.99, 136.79, 136.53, 134.27, 131.99, 130.71, 127.79, 127.56, 126.76, 123.72, 121.29, 118.76, 83.68, 24.89. ESI-TOF (*m/z*): Calcd. for $[C_{68}H_{61}B_2N_6O_4]^+$ and $[C_{68}H_{62}B_2N_6O_4]^{2+}$: 1047.49, 524.25. Found for $[M+H]^+$ and $[M+2H]^{2+}$: 1047.55, 524.26.

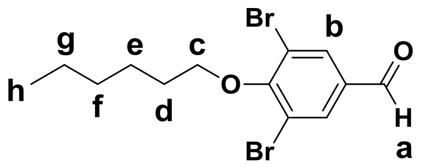

Compound 9: A mixture of 3,5-dibromo-4-hydroxybenzaldehyde (3.0 g, 10.8 mmol), 1-bromohexane (2.1 g, 13.0 mmol), K_2CO_3 (4.5 g, 32.4 mmol), DMF (20 mL) and THF (80 mL) was stirred at 85 ℃ under argon. After 12 h, the mixture was cooled to room temperature. After the white solid in the mixture was filtered off, the solvents were removed under reduced pressure, and the crude product was purified by column chromatography with hexane as eluent to afford compound **9** as yellowish oil (3.64 g, 93%). ^{1}H NMR (400 MHz, $CDCl_3$) δ 9.85 (s, 1H, H^a), 8.02 (s, 2H, Ph-H^b), 4.09 (t, J = 6.6 Hz, 2H, Alkyl-H^c), 1.94 – 1.85 (m, 2H, Alkyl-H^d), 1.35 (m, 6H, Alkyl-H^e, Alkyl-H^f and Alkyl-H^g), 0.92 (t, J = 6.8 Hz, 3H, Alkyl-H^h). ^{13}C NMR (125 MHz, $CDCl_3$) δ 188.42, 158.56, 133.93, 133.89, 119.47, 74.14, 31.57, 29.98, 25.44, 22.58, 14.05.

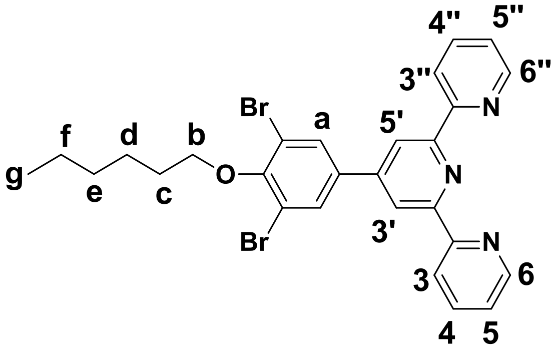

Compound 10: To a solution of NaOH powder (2.1 g, 54 mmol) in 50 mL of EtOH, **9** (3.6 g, 10 mmol) and 2-acetylpyridine (2.7 g, 22 mmol) were added. After stirring at 25 ℃ for 10 h, aqueous $NH_3 \cdot H_2O$ (35 mL) was added and the mixture was refluxed for 20 h. After cooling to room temperature, the suspension was filtered to give an off-white solid. Then the crude was washed with ethanol, dried in vacuo to afford compound **10** as white solid (2.9 g, 52%). ^{1}H NMR (500 MHz, $CDCl_3$) δ 8.74 (d, J = 4.2 Hz, 2H, tpy-$H^{6,6''}$), 8.66 (d, J = 7.9 Hz, 2H, tpy-$H^{3,3''}$), 8.63 (s, 2H, tpy-$H^{3',5'}$), 8.03 (s, 2H, Ph-H^a), 7.88 (td, J = 7.8, 1.6 Hz, 2H, tpy-$H^{4,4''}$), 7.40 – 7.34 (m, 2H, tpy-$H^{5,5''}$), 4.08 (t, J = 6.6 Hz, 2H, Alkyl-H^b), 1.95 – 1.88 (m, 2H, Alkyl-H^c), 1.60 – 1.53 (m, 2H,

Alkyl-H^d), 1.43 – 1.34 (m, 4H, Alkyl-H^e and Alkyl-H^f), 0.93 (t, J = 7.0 Hz, 3H, Alkyl-H^g). ^{13}C NMR (125 MHz, $CDCl_3$) δ 156.14, 155.79, 154.11, 149.13, 147.11, 136.94, 136.73, 131.36, 124.05, 121.38, 119.00, 118.42, 73.74, 31.66, 29.93, 25.55, 22.63, 14.09. ESI-TOF (*m/z*): Calcd. for $[C_{27}H_{26}Br_2N_3O]^+$: 566.04. Found for $[M+H]^+$: 566.06.

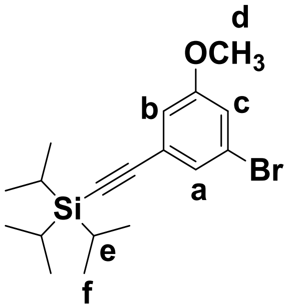

Compound 11: To a Schlenk flask containing 3,5-Dibromoanisole (3.0 g, 11.37 mmol), $Pd(PPh_3)_2Cl_2$ (239 mg, 0.34 mmol) and CuI (65 mg, 0.34 mmol) were added. After the removal of air and back-filled with nitrogen, 50 mL of trimethylamine, 20 mL of tetrahydrofuran and (triisopropylsilyl)acetylene (2.5 mL, 11.37 mmol) were added, and the suspension was stirred at 65 ℃ for 12 h. After the solvent was removed under reduced pressure, the crude product was purified by silica gel chromatography with hexane as eluent to afford compound **11** as colorless oil (3.6 g, 86%).^{1}H NMR (500 MHz, $CDCl_3$) δ 7.20 (t, J = 1.5 Hz, 1H, Ph-H^a), 7.03 – 7.00 (m, 1H, Ph-H^c), 6.91 (dd, J = 2.3, 1.2 Hz, 1H, Ph-H^b, 3.79 (s, 3H, Ph-H^d), 1.12 (s, 21H, H^e and H^f). ^{13}C NMR (125 MHz, $CDCl_3$) δ 159.85, 127.21, 125.84, 122.35, 117.92, 116.09, 105.25, 92.07, 55.55, 18.63, 11.24.

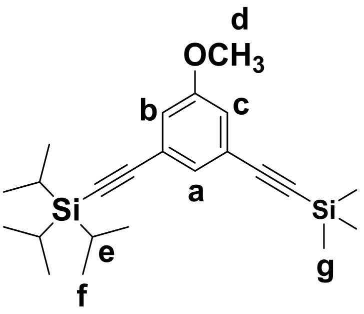

Compound 12: To a Schlenk flask containing compound **11** (4.0 g, 11 mmol), $Pd(PPh_3)_2Cl_2$ (230 mg, 0.33 mmol) and CuI (63 mg, 0.33 mmol) were added. After the removal of air and back-filled with nitrogen, 60 mL of trimethylamine, 30 mL of tetrahydrofuran and trimethylsilylacetylene (2.3 mL, 16 mmol) were added, and the suspension was stirred at 65 ℃ for 12 h. After the solvent was removed under reduced pressure, the crude product was purified by silica gel chromatography with hexane as eluent to afford compound **12** as colorless oil (3.7 g, 88%). ^{1}H NMR (500 MHz, $CDCl_3$) δ 7.21 – 7.15 (m, 1H, Ph-H^a), 6.96 – 6.92 (m, 2H, Ph-H^b and Ph-H^c), 3.79 (s, 3H, Ph-H^d), 1.12 (s, 21H, H^e and H^f), 0.26 – 0.22 (m, 9H, H^g). ^{13}C NMR (100 MHz, $CDCl_3$) δ 159.18, 128.32, 124.79, 124.37, 118.19, 117.44, 106.16, 104.25, 94.74, 91.21, 55.54, 18.81, 11.45, 0.05.

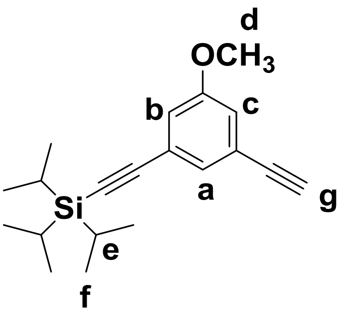

Compound 13: Potassium carbonate (3.2 g, 23.4 mmol) was added to a solution of compound **12** (3.0 g, 7.8 mmol) in tetrahydrofuran/methanol (30 mL/30 mL). The mixture was stirred at room temperature for 5 h. After that, the solvent was removed under reduced pressure, and the crude product was extracted with ethyl acetate. The combined organic layer was washed with water (for three times) and brine, dried over anhydrous Na_2SO_4, and then concentrated in vacuo. The crude prude was purified by column chromatography on silica gel (hexane) to afford the product as a colorless oil

(2.4 g, 87%). ^{1}H NMR (400 MHz, $CDCl_3$) δ 7.21 (s, 1H, Ph-H^a), 6.97 (s, 2H, Ph-H^b and Ph-H^c), 3.80 (s, 3H, Ph-H^d), 3.07 (d, J = 13.9 Hz, 1H, H^g), 1.12 (s, 21H, H^e and H^f). ^{13}C NMR (125 MHz, $CDCl_3$) δ 159.05, 128.31, 124.76, 123.14, 118.09, 117.71, 105.82, 91.22, 82.87, 77.45, 55.57, 18.63, 11.26.

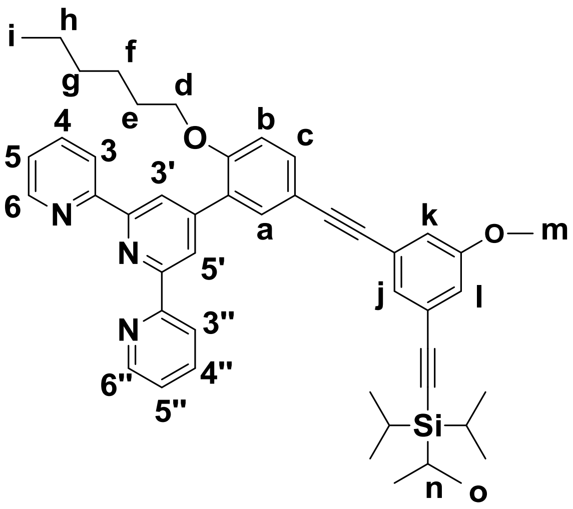

Compound 14: To a Schlenk flask containing compound **13** (700 mg, 2.24 mmol), compound **6** (1.09 g, 2.24 mmol), $Pd(PPh_3)_4$ (77 mg, 0.067 mmol) and CuI (13 mg, 0.067 mmol) were added. After the removal of air and back-filled with nitrogen, 20 mL of trimethylamine, 10 mL of DMF and 10 mL of DME were added, and the suspension was stirred at 80 ℃ for 12 h. After the solvent was removed under reduced pressure, the crude product was purified by silica gel chromatography with hexane as eluent to afford compound **14** as yellowish solid (886 mg, 55%). ^{1}H NMR (500 MHz, $CDCl_3$) δ 8.73 (s, 2H, tpy-$H^{3',5'}$), 8.71 (d, J = 4.1 Hz, 2H, tpy-$H^{6,6''}$), 8.68 (d, J = 7.9 Hz, 2H, tpy-$H^{3,3''}$), 7.87 (td, J = 7.7, 1.7 Hz, 2H, tpy-$H^{4,4''}$), 7.77 (d, J = 2.1 Hz, 1H, Ph-H^a), 7.54 (dd, J = 8.5, 2.1 Hz, 1H, Ph-H^c), 7.37 – 7.31 (m, 2H, tpy-$H^{5,5''}$), 7.27 (dd, J = 2.5, 1.1 Hz, 1H, Ph-H^j), 7.03 (dd, J = 2.3, 1.3 Hz, 1H, Ph-H^k), 6.99 – 6.96 (m, 1H, Ph-H^l), 6.96 (dd, J = 2.4, 1.3 Hz, 1H, Ph-H^b), 4.05 (t, J = 6.2 Hz, 2H, Alkyl-H^d), 3.83 (s, 3H, Alkyl-H^m), 1.77 – 1.69 (m, 2H, Alkyl-H^e), 1.46 – 1.34 (m, 2H, Alkyl-H^f), 1.24 – 1.14 (m, 4H, Alkyl-H^g and Alkyl-H^h), 0.73 (t, J = 7.1 Hz, 3H, Alkyl-H^i). ^{13}C NMR (125 MHz, $CDCl_3$) δ 159.08, 156.53, 156.33, 155.11, 149.02, 147.42, 136.66, 133.92, 133.32, 128.45, 127.70, 124.60, 123.54, 121.68, 121.13, 117.48, 117.02, 116.72,

115.06, 112.06, 106.15, 90.94, 89.51, 87.69, 68.56, 55.38, 31.47, 29.01, 25.73, 22.30, 18.63, 13.88, 11.24. ESI-TOF (*m/z*): Calcd. for $[C_{47}H_{54}N_3O_2Si]^+$: 720.40, Found for $[M+H]^+$: 720.41.

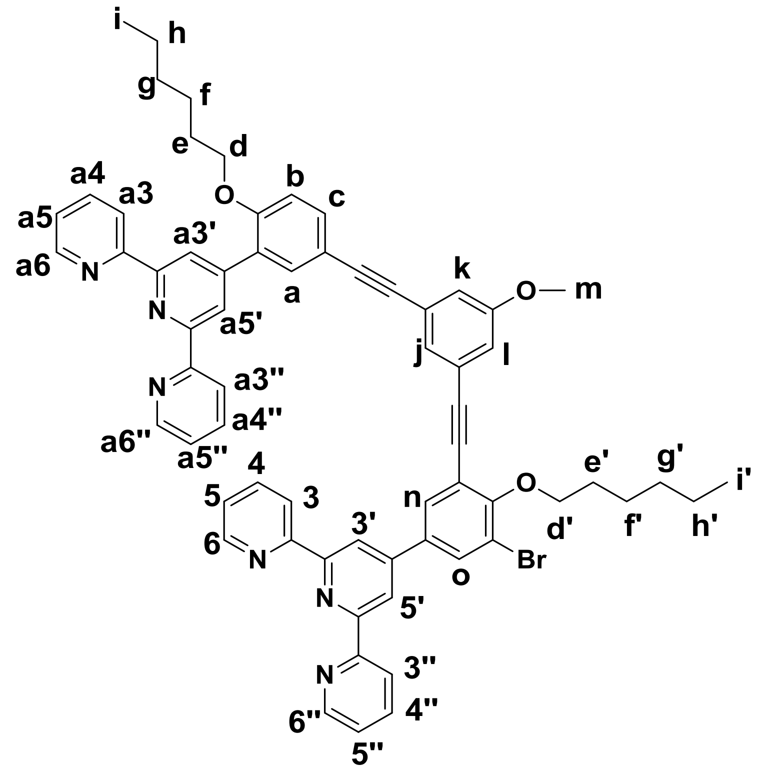

Compound 15: To a Schlenk flask containing compound **14** (500 mg, 0.70 mmol), compound **10** (395 mg, 0.70 mmol), $Pd(PPh_3)_4$ (24 mg, 0.021 mmol) and CuI (4 mg, 0.021 mmol) were added. After the removal of air and back-filled with argon, 15 mL of trimethylamine, 10 mL of DMF and 10 mL of DME were added. Then 1.05 mL of tetrabutylammonium fluoride (1.05 mmol) in THF (1.0 M) was added to the refluxing mixture under argon and the suspension was stirred at 70 ℃ for 15 h. After the solvent was removed under reduced pressure, the crude product was purified by silica gel chromatography with chloroform (with 1.5% ethanol) as eluent to afford compound **15** as yellowish solid (382 mg, 52%). ^{1}H NMR (500 MHz, $CDCl_3$) δ 8.73 (d, J = 4.1 Hz, 2H, tpy-$H^{6,6''}$), 8.71 (s, 2H, tpy-$H^{3',5'}$), 8.71 – 8.69 (m, 2H, tpy-$H^{a6,a6''}$), 8.68 (s, 2H, tpy-$H^{a3',a5'}$), 8.66 (dd, J = 4.9, 3.0 Hz, 4H, tpy-$H^{3,3''}$ and tpy-$H^{a3,a3''}$), 8.07 (t, J = 5.9 Hz, 1H, Ph-H^{a}), 8.01 (d, J = 2.2 Hz, 1H, Ph-H^{n}), 7.87 (ddd, J = 13.2, 7.4, 1.6 Hz, 4H, tpy-$H^{4,4''}$ and tpy-$H^{a4,a4''}$), 7.79 (t, J = 4.2 Hz, 1H, Ph-H^{o}), 7.55 (td, J = 8.8, 1.9

Hz, 1H, Ph-H^{c}), 7.36 (d, J = 1.0 Hz, 1H, Ph-H^{j}), 7.35 – 7.29 (m, 4H, tpy-$H^{5,5''}$ and tpy-$H^{a5,a5''}$), 7.08 (dt, J = 9.2, 4.6 Hz, 2H, Ph-H^{k} and Ph-H^{l}), 6.98 (t, J = 6.6 Hz, 1H, Ph-H^{b}), 4.28 (t, J = 6.5 Hz, 2H, Alkyl-H^{d}), 4.05 (t, J = 6.2 Hz, 2H, Alkyl-$H^{d'}$), 3.87 (s, 3H, Alkyl-H^{m}), 1.93 (dq, J = 13.8, 6.9 Hz, 2H, Alkyl-H^{e}), 1.74 (dd, J = 14.4, 7.1 Hz, 2H, Alkyl-$H^{e'}$), 1.61 (dd, J = 14.8, 7.5 Hz, 2H, Alkyl-H^{f}), 1.43 – 1.34 (m, 6H, Alkyl-$H^{f'}$, Alkyl-$H^{g'}$ and Alkyl-H^{g}), 1.18 (dd, J = 10.4, 5.3 Hz, 2H, Alkyl-H^{h}), 1.15 – 1.11 (m, 2H, Alkyl-$H^{h'}$), 0.90 – 0.85 (m, 3H, Alkyl-H^{i}), 0.73 (t, J = 7.1 Hz, 3H, Alkyl-$H^{i'}$). ^{13}C NMR (100 MHz, $CDCl_3$) δ 159.30, 157.87, 156.62, 156.38, 156.04, 155.88, 155.16, 149.08, 147.63, 147.45, 136.85, 136.69, 135.00, 133.96, 133.42, 132.16, 131.58, 128.53, 127.13, 124.97, 123.94, 123.58, 121.73, 121.34, 121.16, 119.08, 118.34, 117.34, 117.00, 115.11, 112.14, 94.03, 89.78, 87.65, 85.22, 77.36, 77.04, 76.72, 74.55, 68.65, 55.48, 31.75, 31.51, 30.38, 29.06, 25.85, 25.77, 22.65, 22.35, 14.09, 13.92. ESI-TOF (*m/z*): Calcd. for $[C_{65}H_{58}BrN_6O_3]^{+}$ and $[C_{65}H_{59}BrN_6O_3]^{2+}$: 1049.38 and 525.19. Found for $[M+H]^{+}$ and $[M+2H]^{2+}$: 1049.36 and 525.20.

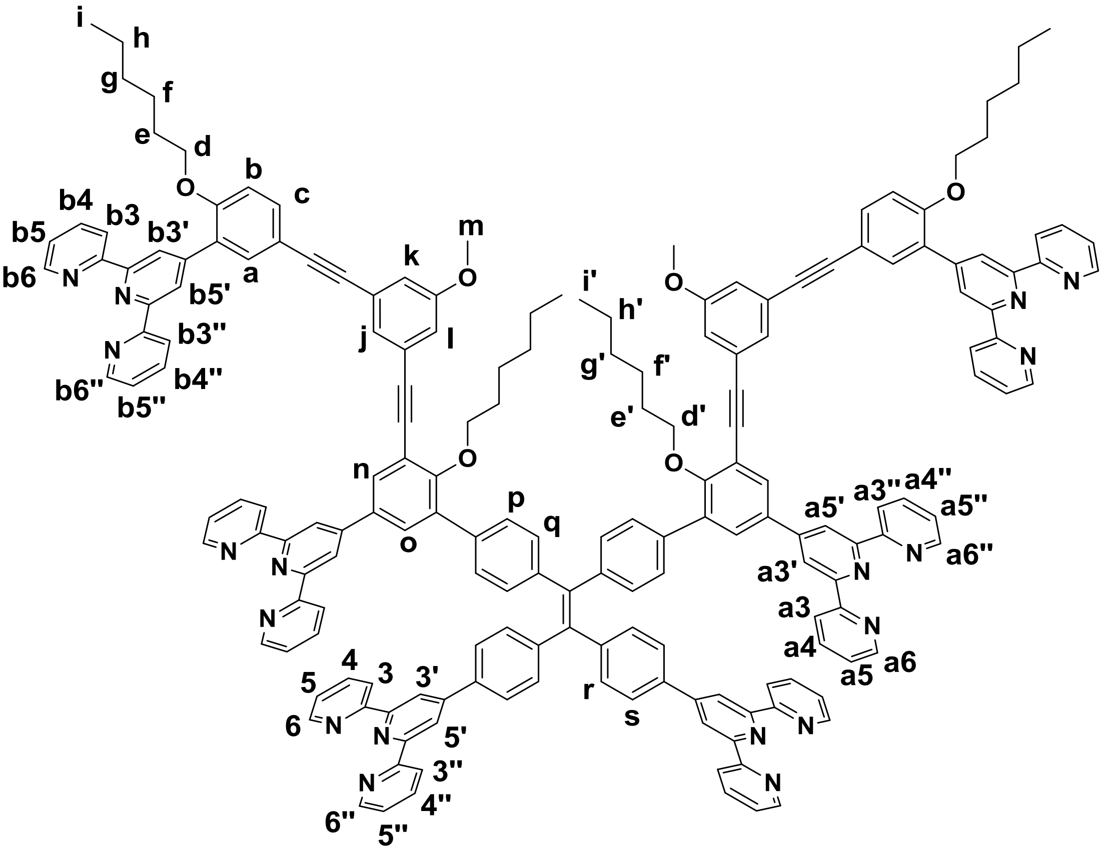

Ligand L3: Under argon, a mixture of compound **15** (200 mg, 0.19 mmol), compound **8** (94 mg, 0.09 mmol), $Pd(PPh_3)_4$ (6 mg, 5.2 μmol), K_2CO_3 (75 mg, 0.54 mmol), in 15 mL of toluene , 15 mL of water and 5 mL of *t*-butanol was stirred at 85 ℃ for 24 h. 100 mL of water was added and DCM was used to extract for three times. After that, the organic layer was washed for three times with water. DCM was removed and the residue was purified by silica gel chromatography with chloroform (with 2% ethanol) as eluent to afford **L3** in 56% yield as a yellowish solid. ^{1}H NMR (500 MHz, $CDCl_3$) δ 8.73 (s, 8H, tpy-$H^{a3',a5'}$ and tpy-$H^{b3',b5'}$), 8.71 (s, 4H, tpy-$H^{3',5'}$), 8.70 – 8.68 (m, 4H, tpy-$H^{6,6''}$), 8.66 (m, 12H, tpy-$H^{a6,a6''}$, tpy-$H^{b6,b6''}$ and tpy-$H^{3,3''}$), 8.62 (d, *J* = 8.2 Hz, 8H, tpy-$H^{a3,a3''}$ and tpy-$H^{b3,b3''}$), 8.05 (s, 2H, Ph-H^{a}), 7.90 (s, 2H), Ph-H^{o}, 7.86 – 7.80 (m, 12H, tpy-$H^{4,4''}$, tpy-$H^{a4,a4''}$ and tpy-$H^{b4,b4''}$), 7.78 – 7.76 (m, 4H, Ph-H^{s}), 7.75 (s, 2H, Ph-H^{n}), 7.53 (d, *J* = 6.7 Hz, 6H, Ph-H^{c} and Ph-H^{p}), 7.35 (d, *J* = 8.0 Hz, 8H, Ph-H^{r} and Ph-H^{q}), 7.30 (dd, *J* = 6.4, 4.9 Hz, 14H, tpy-$H^{5,5''}$, tpy-$H^{a5,a5''}$, tpy-$H^{b5,b5''}$ and Ph-H^{j}), 7.07 (d, *J* = 1.1 Hz, 4H, Ph-H^{k} and Ph-H^{l}), 6.97 (d, *J* = 8.6 Hz,

2H, Ph-H^b), 4.04 (t, J = 5.4 Hz, 4H, Alkyl-H^d), 3.87 (d, J = 5.1 Hz, 4H, Alkyl-$H^{d'}$), 3.84 (s, 6H, Alkyl-H^m), 1.71 (m, 4H, Alkyl-H^e), 1.62 – 1.57 (m, 4H, Alkyl-$H^{e'}$), 1.44 – 1.39 (m, 4H, Alkyl-H^f), 1.36 (m, 4H, Alkyl-$H^{f'}$), 1.20 – 1.16 (m, 4H, Alkyl-H^g), 1.16 – 1.06 (m, 12H, Alkyl-$H^{g'}$, Alkyl-H^h and Alkyl-$H^{h'}$), 0.74 (dd, J = 10.0, 4.2 Hz, 6H, Alkyl-H^i), 0.68 (t, J = 5.6 Hz, 6H, Alkyl-$H^{i'}$). ^{13}C NMR (125 MHz, $CDCl_3$) δ 159.21, 158.67, 156.54, 156.38, 156.18, 156.10, 155.90, 155.83, 155.15, 149.82, 149.07, 148.69, 147.49, 144.54, 142.79, 142.64, 141.78, 140.17, 139.83, 136.69, 136.20, 135.87, 133.91, 133.76, 133.42, 133.33, 132.13, 131.86, 131.36, 130.05, 129.03, 128.49, 127.17, 126.82, 124.76, 124.52, 123.77, 123.68, 123.57, 121.75, 121.29, 121.16, 118.77, 118.56, 118.48, 118.34, 117.11, 116.89, 116.75, 115.19, 115.00, 112.11, 92.77, 89.53, 87.76, 86.38, 74.16, 68.62, 55.44, 31.58, 31.50, 30.27, 29.05, 25.93, 25.76, 22.57, 22.33, 13.98, 13.91. ESI-TOF (*m/z*): Calcd. for and $[C_{186}H_{152}N_{18}O_6]^{2+}$, $[C_{186}H_{153}N_{18}O_6]^{3+}$ and $[C_{186}H_{154}N_{18}O_6]^{4+}$: 1366.60, 911.41 and 683.21. Found for $[M+H]^{2+}$, $[M+2H]^{3+}$ and $[M+3H]^{4+}$: 1366.48, 911.40 and 683.81. MALDI-TOF MS (*m/z*): Calcd. for $[C_{186}H_{151}N_{18}O_6]^+$: 2732.20, Found: 2732.81.

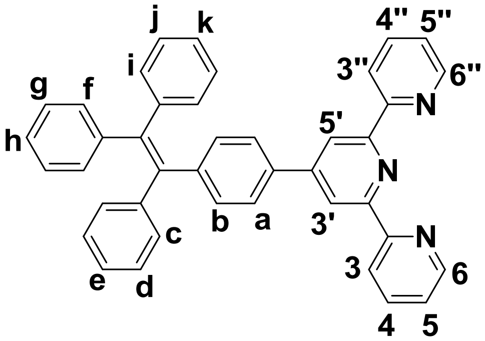

Compound 16: Under argon, a mixture of 2-Bromo-1,1,2-triphenylethylene (1.0 g, 2.98 mmol), compound **2** (2.11 g, 5.97 mmol), $Pd(PPh_3)_4$ (172 mg, 0.15 mmol), and Na_2CO_3 (3.16 g, 29.83 mmol), in 16 mL of toluene , 4 mL of water and 4 mL of ethanol was stirred at 85 ℃ for 12 h. 50 mL of water was added and DCM was used to extract for three times. After that, the organic layer was washed for three times with water. DCM was removed and the residue was purified by silica gel chromatography with chloroform (with 0.5% ethanol) as eluent to afford compound **16** in 88% yield as a light yellow solid. ^{1}H NMR (400 MHz, $CDCl_3$) δ 8.71 (d, J = 4.2 Hz, 2H, tpy-$H^{6,6''}$), 8.67 (s, 2H, tpy-$H^{3',5'}$), 8.65 (d, J = 7.8 Hz, 2H, tpy-$H^{3,3''}$), 7.87 (td, J = 7.8, 1.6 Hz, 2H, tpy-$H^{4,4''}$), 7.65 (d, J = 8.3 Hz, 2H, Ph-H^{a}), 7.34 (dd, J = 6.6, 5.0 Hz, 2H, tpy-$H^{5,5''}$), 7.16 (d, J = 8.3 Hz, 2H, Ph-H^{b}), 7.15 – 7.03 (m, 15H, Ph-H^{c-k}). ^{13}C NMR (101 MHz, $CDCl_3$) δ 156.39, 155.95, 150.08, 149.21, 144.86, 143.74, 143.66, 143.61, 141.71, 140.42, 136.95, 136.38, 132.04, 131.52, 131.45, 127.98, 127.87, 127.79, 126.84, 126.73, 126.70, 126.65, 123.89, 121.43, 118.83. ESI-TOF (*m/z*): Calcd. for, $[C_{41}H_{30}N_3]^+$: 564.24. Found for $[M+H]^+$: 564.71.

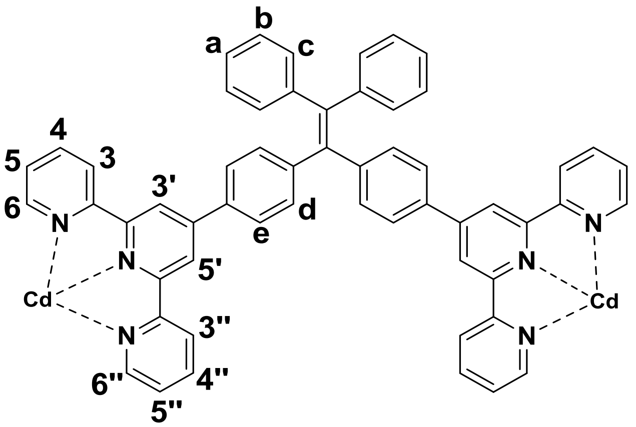

G1: To a solution of ligand **L1** (5.0 mg, 6.29 μmol) in $CHCl_3$ (1.5 mL), a solution of $Cd(NO_3)_2 \cdot 4H_2O$ (1.9 mg, 6.29 μmol) in MeOH (4.5 mL) was added, and then the mixture was stirred at 50 ℃ for 8 h. After cooling to room temperature, 161 mg of NH_4PF_6 was added and bright yellow precipitate was observed. The precipitate was washed by distilled water and the final product was obtained with a yield of 93%. ^{1}H NMR (400 MHz, CD_3CN) δ 8.87 (br, 4H, tpy-$H^{3',5'}$), 8.73 (d, J = 7.1 Hz, 4H, tpy-$H^{3,3''}$), 8.18 (m, 4H, tpy-$H^{4,4''}$), 8.04 (m, 8H, tpy-$H^{6,6''}$and Ph-H^{e}), 7.48 (m, 8H, tpy-$H^{5,5''}$and Ph-H^{d}), 7.26 (br, 10H Ph-H^{a}, Ph-H^{b} and Ph-H^{c}). ^{13}C NMR (100 MHz, CD_3CN) δ 155.35, 151.22, 150.37, 143.40, 142.20, 135.18, 135.08, 133.15, 131.88, 128.92, 128.44, 128.25, 122.28. ESI-MS (*m/z*): 573.5 [M3-5PF_6]$^{5+}$ (calcd *m/z*: 573.5), 1451.2 [M4-3PF_6]$^{3+}$ (calcd *m/z*: 1451.2), 813.0 [M4-5PF_6]$^{5+}$ (calcd *m/z*: 813.0), 539.1 [M4-7PF_6]$^{7+}$ (calcd *m/z*: 539.1), 1351.7 [M5-4PF_6]$^{4+}$ (calcd *m/z*: 1351.7), 852.8 [M5-6PF_6]$^{6+}$ (calcd *m/z*: 852.8), 710.3 [M5-7PF_6]$^{7+}$ (calcd *m/z*: 710.3), 603.4 [M5-8PF_6]$^{8+}$ (calcd *m/z*: 603.4), 881.3 [M6-7PF_6]$^{7+}$ (calcd *m/z*: 881.3). Macrocycle complexes are named Mn^{x+}, where M designates the repeat unit <tpy-Cd-tpy>, n is the number of repeat units, and x is the number of charges.

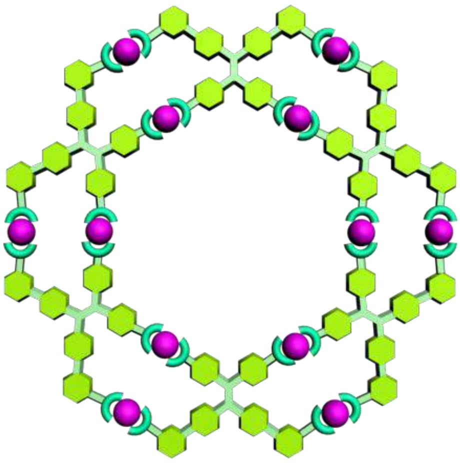

G2: To a solution of ligand **L2** (6.2 mg, 4.1 μmol) in $CHCl_3$ (1.0 mL), a solution of $Cd(NO_3)_2 \cdot 4H_2O$ (2.5 mg, 8.2 μmol) in MeOH (3 mL) was added, and then the mixture was stirred at 50 ℃ for 8 h. After cooling to room temperature, 210 mg of NH_4PF_6 was added and bright yellow precipitate was observed. The precipitate was washed by water, and the final product was obtained with a yield of 90%. ^{1}H NMR (500 MHz, CD_3CN) δ 9.03 (s, 4H, tpy-$H^{a3',a5'}$), 8.95 (d, J = 10.3 Hz, 4H, tpy-$H^{3',5'}$), 8.66 (m, 4H, tpy-$H^{3,3''}$), 8.54 (m, 4H, tpy-$H^{a3,a3''}$), 8.13 (m, 4H, Ph-H^{m}), 8.06 (d, J = 13.6 Hz, 2H, Ph-H^{a}), 8.03 (s, 4H, tpy-$H^{6,6''}$), 7.97 (s, 4H, tpy-$H^{a6,a6''}$), 7.88 (dd, J = 16.7, 8.9 Hz, 6H, Ph-H^{j}, tpy-$H^{4,4''}$), 7.80 (s, 4H, tpy-$H^{a4,a4''}$), 7.74 – 7.70 (m, 2H, Ph-H^{c}), 7.64 (s, 4H, Ph-H^{l}), 7.42 (s, 6H, Ph-H^{b} and Ph-H^{k}), 7.14 (d, J = 17.7 Hz, 4H, tpy-$H^{5,5''}$), 7.02 (m, 4H, tpy-$H^{a5,a5''}$), 4.29 (s, 4H, Alkyl-H^{d}), 1.80 (s, 4H, Alkyl-H^{e}), 1.49 (s, 4H, Alkyl-H^{f}), 1.26 (s, 4H, Alkyl-H^{g}), 1.15 – 1.08 (m, 4H, Alkyl-H^{h}), 0.66 (d, J = 6.8 Hz, 6H, Alkyl-H^{i}). ^{13}C DEPT 45° NMR (125 MHz, CD_3CN) δ 150.39, 141.84, 141.77, 132.97, 132.80, 130.30, 128.75, 128.67, 127.98, 127.63, 127.58, 125.37, 125.35, 124.56, 124.53, 124.16, 122.66, 122.54, 122.48, 118.15, 114.35, 69.82, 32.22, 29.95, 26.62, 23.09, 14.06. ESI-MS (*m/z*): 1303.7 $[M-10PF_6]^{10+}$ (calcd *m/z*: 1303.7), 1172.1 $[M-11PF_6]^{11+}$ (calcd *m/z*: 1172.1), 1062.4 $[M-12PF_6]^{12+}$ (calcd *m/z*: 1062.4), 969.5 $[M-13PF_6]^{13+}$ (calcd *m/z*: 969.5), 889.8 $[M-14PF_6]^{14+}$ (calcd *m/z*: 889.8), 820.9 $[M-15PF_6]^{15+}$ (calcd *m/z*: 820.9), 760.6 $[M-16PF_6]^{16+}$ (calcd *m/z*: 760.6), 707.3 $[M-17PF_6]^{17+}$ (calcd *m/z*: 707.3), 659.9 $[M-18PF_6]^{18+}$ (calcd *m/z*: 659.9).

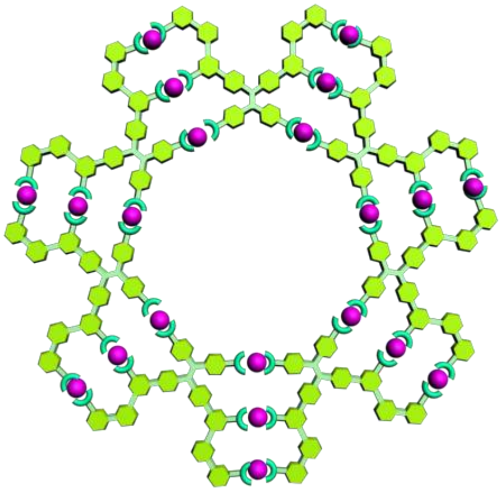

G3: To a solution of ligand **L3** (5.5 mg, 2.0 μmol) in $CHCl_3$ (2.0 mL), a solution of $Cd(NO_3)_2 \cdot 4H_2O$ (1.8 mg, 6.0 μmol) in MeOH (6 mL) was added, and then the mixture was stirred at 50 °C for 8 h. After cooling to room temperature, 154 mg of NH_4PF_6 was added and bright yellow precipitate was observed. The precipitate was washed by water, and the final product was obtained with a yield of 91%. ^{1}H NMR (500 MHz, CD_3CN) δ 9.00 (d, *J* = 15.7 Hz, 12H, tpy-$H^{3',5'}$, tpy-$H^{a3',a5'}$ and tpy-$H^{b3',b5'}$), 8.68 (d, *J* = 6.6 Hz, 12H, tpy-$H^{3,3''}$, tpy-$H^{a3,a3''}$ and tpy-$H^{b3,b3''}$), 8.37 (s, 2H, Ph-H^{a}), 8.13 (dd, *J* = 41.1, 9.2 Hz, 12H, Ph-H^{n}, Ph-H^{o}, tpy-$H^{4,4''}$ and tpy-$H^{a4,a4''}$), 8.03 (s, 8H, tpy-$H^{6,6''}$ and tpy-$H^{a6,a6''}$), 7.87 (s, 10H, Ph-H^{c}, tpy-$H^{b4,b4''}$ and tpy-$H^{b6,b6''}$), 7.70 (s, 8H, Ph-H^{s} and Ph-H^{p}), 7.56 (s, 2H Ph-H^{j}), 7.50 (s, 4H, Ph-H^{q}), 7.43 (s, 4H, Ph-H^{r}), 7.39 (d, *J* = 7.2 Hz, 2H, Ph-H^{b}), 7.32 (s, 2H, Ph-H^{k}), 7.23 (s, 2H, Ph-H^{l}), 7.02 (d, *J* = 50.0 Hz, 12H, tpy-$H^{5,5''}$, tpy-$H^{a5,a5''}$ and tpy-$H^{b5,b5''}$), 4.30 (s, 4H, Alkyl-H^{d}), 4.10 (s, 4H, Alkyl-$H^{d'}$), 4.00 (s, 6H, Alkyl-H^{m}), 1.89 (s, 4H, Alkyl-H^{e}), 1.83 (s, 4H, Alkyl-$H^{e'}$), 1.47 (s, 12H, Alkyl-H^{f}, Alkyl-$H^{f'}$ and Alkyl-$H^{g'}$), 1.25 (s, 8H, Alkyl-H^{g} and Alkyl-H^{h}), 1.10 (s, 4H, Alkyl-$H^{h'}$), 1.03 (s, 6H, Alkyl-H^{i}), 0.64 (s, 6H, Alkyl-$H^{i'}$). ^{13}C DEPT 45° NMR (125 MHz, CD_3CN) δ 150.52, 150.38, 142.20, 141.63, 128.01, 127.81, 127.69, 125.41, 124.29, 118.21, 117.78, 114.33, 75.69, 69.95, 56.36, 32.44, 32.18, 30.99, 29.80, 26.60, 26.48, 23.38, 23.05, 14.50, 14.02. ESI-MS (*m/z*): 1976.9 $[M-13PF_6]^{13+}$ (calcd *m/z*: 1976.9), 1825.3 $[M-14PF_6]^{14+}$ (calcd *m/z*: 1825.3), 1693.7 $[M-15PF_6]^{15+}$ (calcd *m/z*: 1693.7), 1578.9 $[M-16PF_6]^{16+}$ (calcd *m/z*: 1578.9), 1477.6 $[M-17PF_6]^{17+}$ (calcd *m/z*: 1477.6), 1387.4 $[M-18PF_6]^{18+}$ (calcd *m/z*: 1387.4).

ESI-MS spectra data of complex G1, G2, G3 (PF_6^- as counterion).

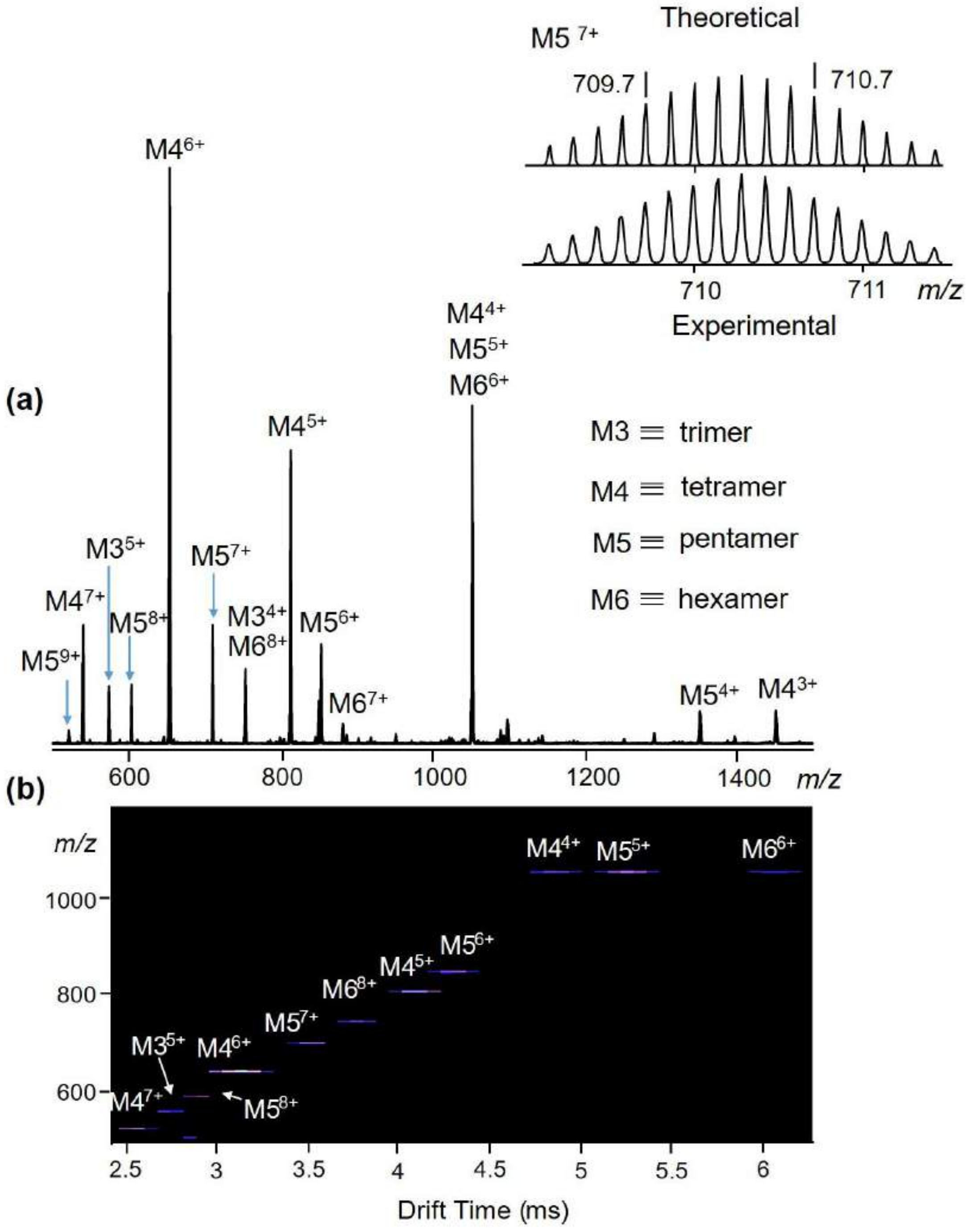

Supplementary Figure 5: (a) ESI-MS and (b) 2D ESI-TWIM-MS plot (*m/z* vs. drift time) of **G1.** Macrocycle complexes are named Mn^{x+}, where M designates the repeat unit <tpy-Cd-tpy>, *n* is the number of repeat units, and *x* is the number of charges.

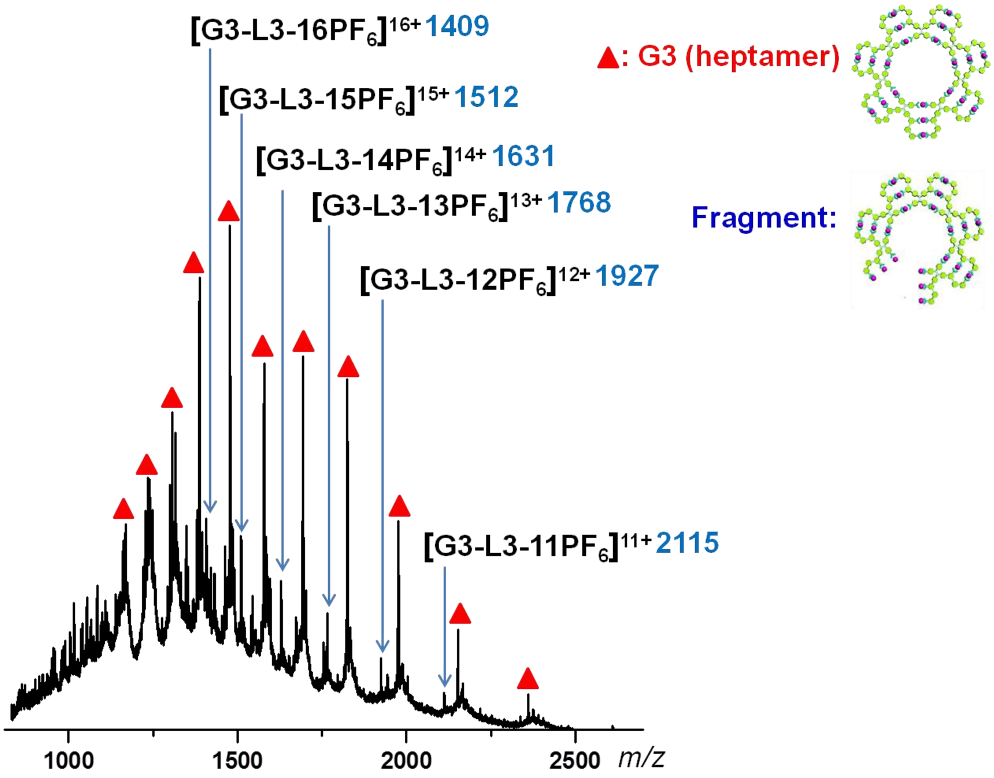

Supplementary Figure 6: ESI-MS of **G3**, the minor signals are assigned as fragments.

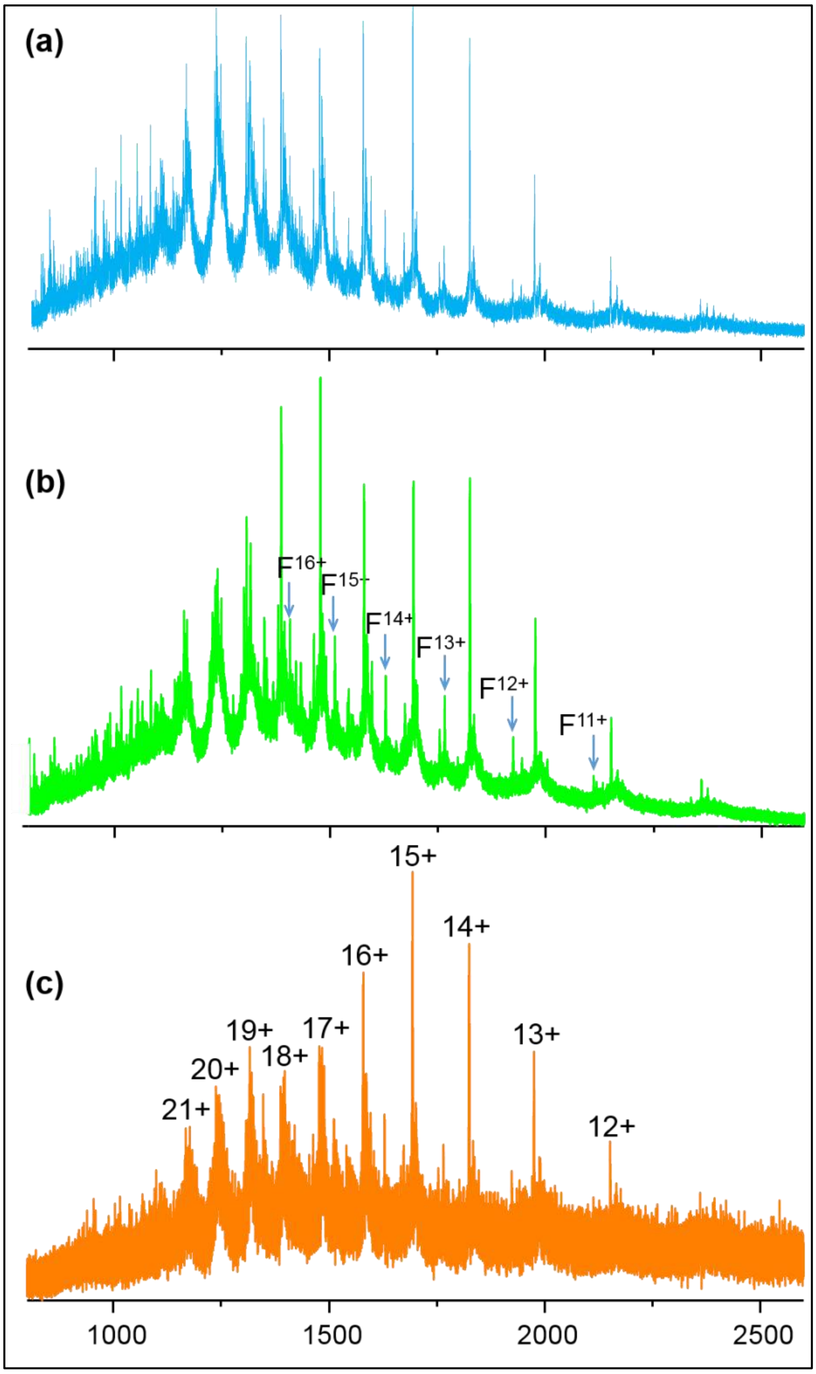

Supplementary Figure 7: ESI-MS of **G3** with different cone voltage (a) 4 kV, (b) 3 kV, (c) 2 kV.

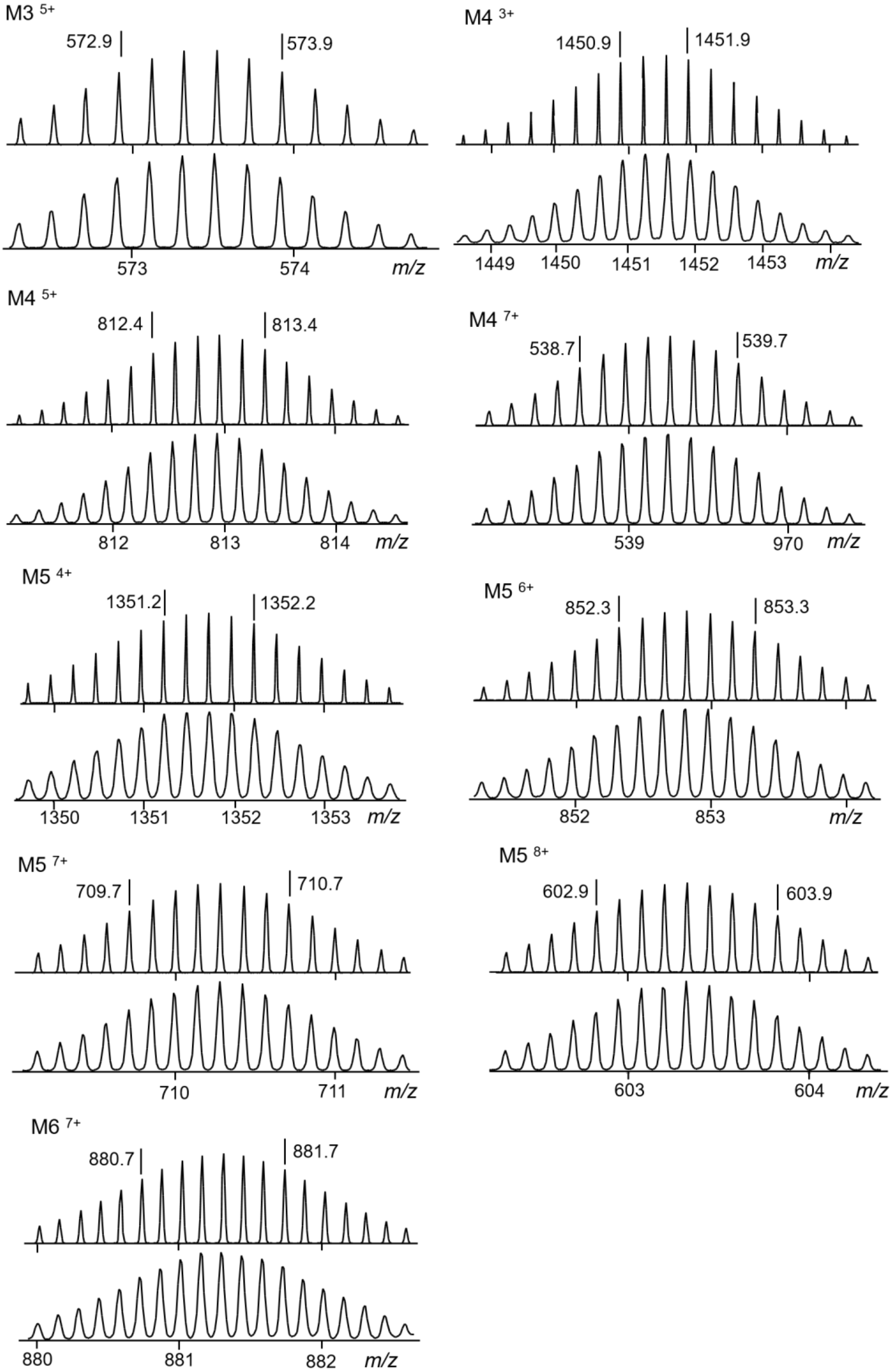

Supplementary Figure 8: Measured (bottom) and calculated (top) isotope patterns for different charge states observed from **G1** (PF_6^- as counterion).

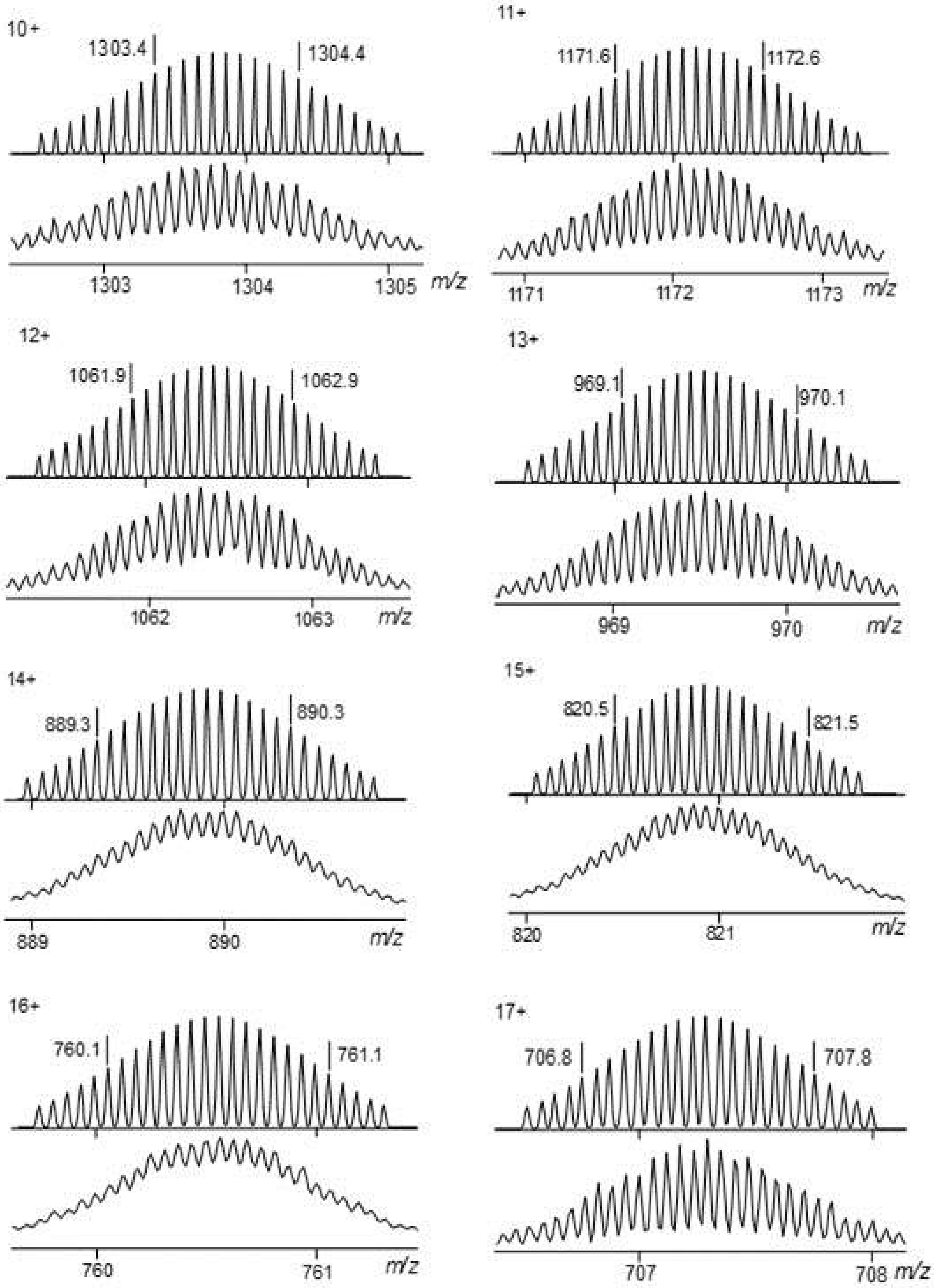

Supplementary Figure 9: Measured (bottom) and calculated (top) isotope patterns for different charge states observed from **G2** (PF_6^- as counterion).

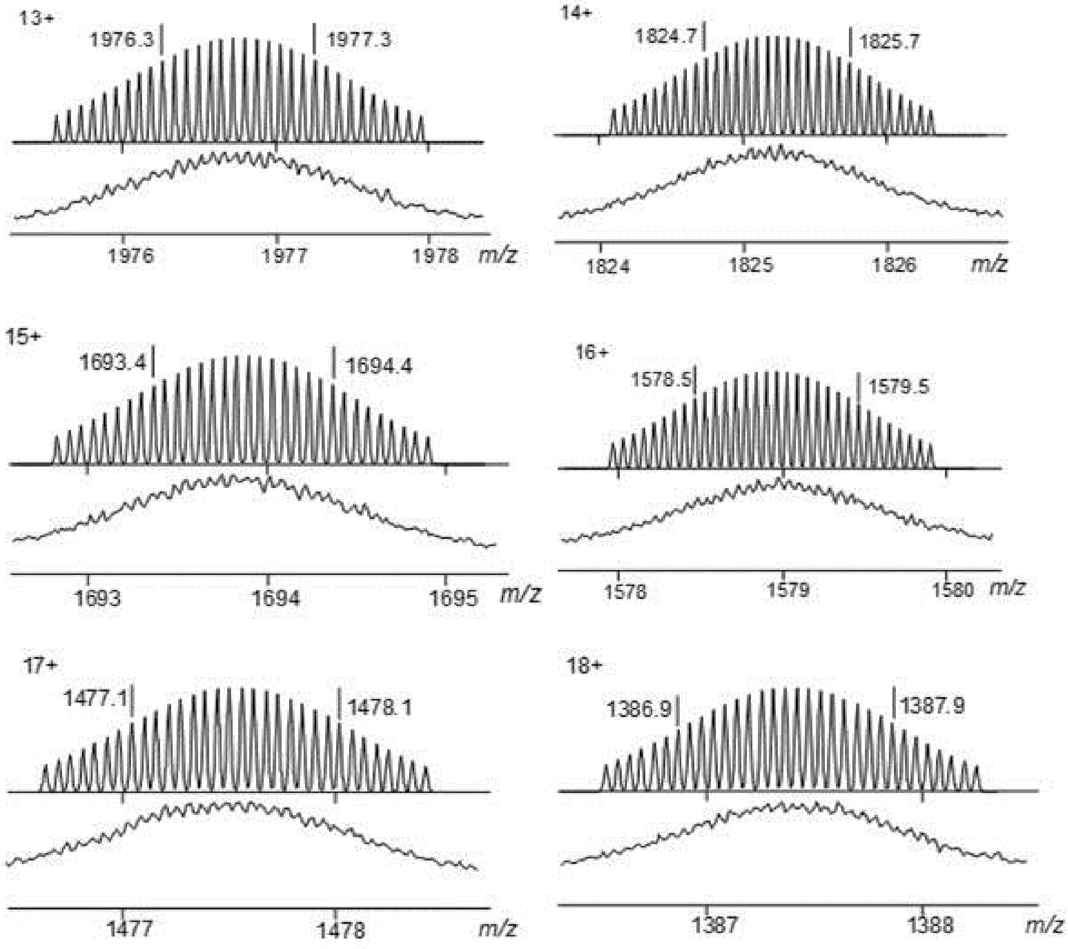

Supplementary Figure 10: Measured (bottom) and calculated (top) isotope patterns for different charge states observed from **G3** (PF_6^- as counterion).

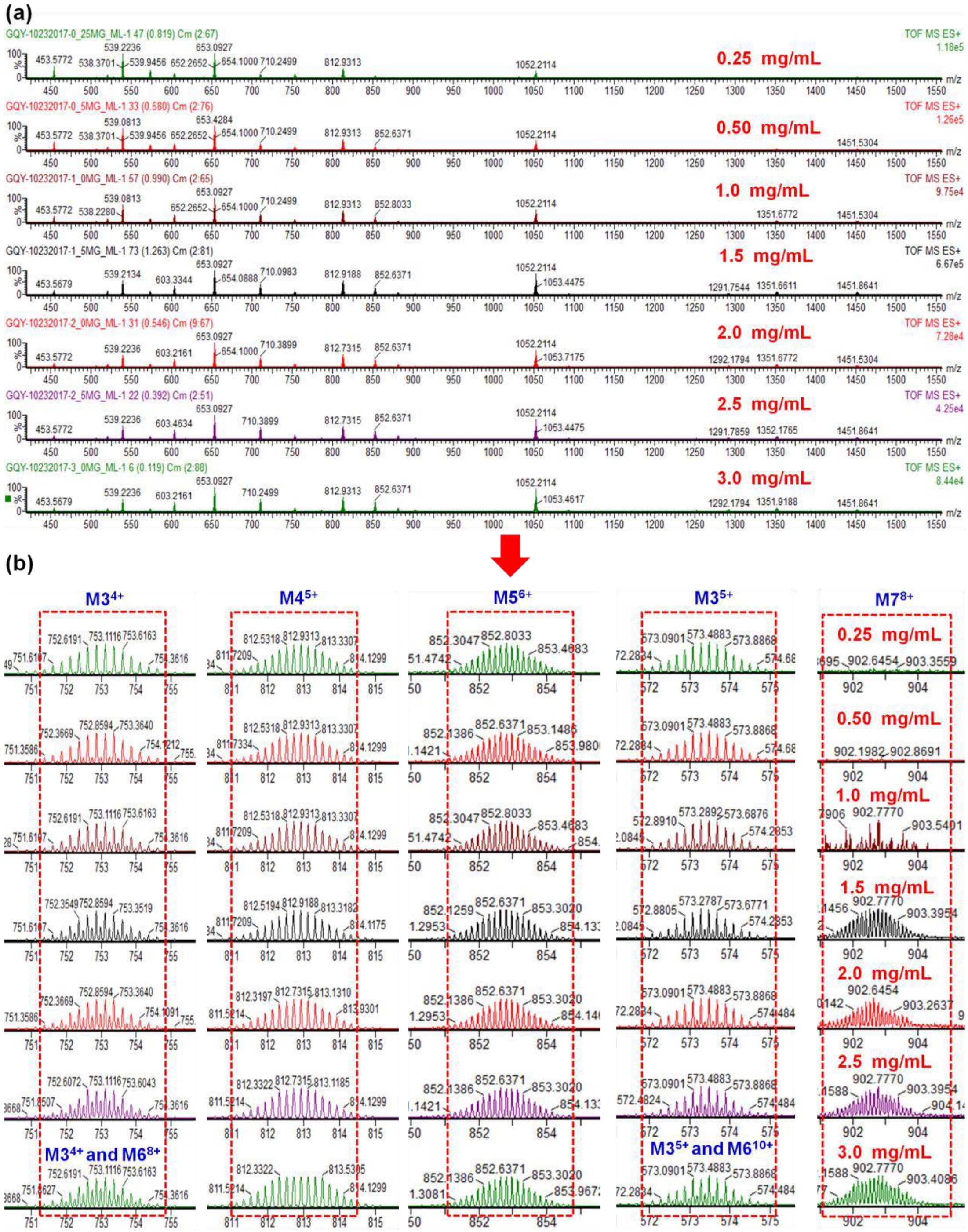

Supplementary Figure 11: (a)Variable concentration ESI-MS spectra of **G1** in CH_3CN/MeOH (v/v, 3/1) from 0.25 mg/mL to 3.0 mg/mL. (b) Expanded at *m/z* = 753, 813, 853, 574, 903. Macrocycle complexes are named Mn^{x+}, where M designates the repeat unit <tpy-Cd-tpy>, n is the number of repeat units, and x is the number of charges.

Molecular modeling

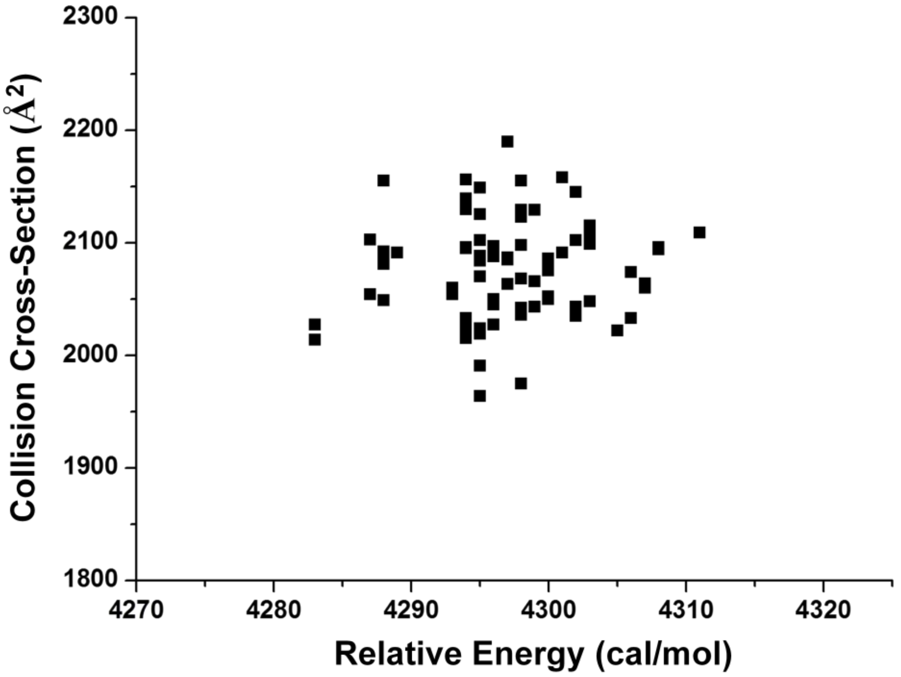

Supplementary Figure 12: Plot of collision cross-section (CCS) *vs.* relative energy for 70 candidate structures of **G2** generated by annealing simulations. CCSs were calculated by the TM method using the MOBCAL program. The average TM cross section area is 2065 Å^2.

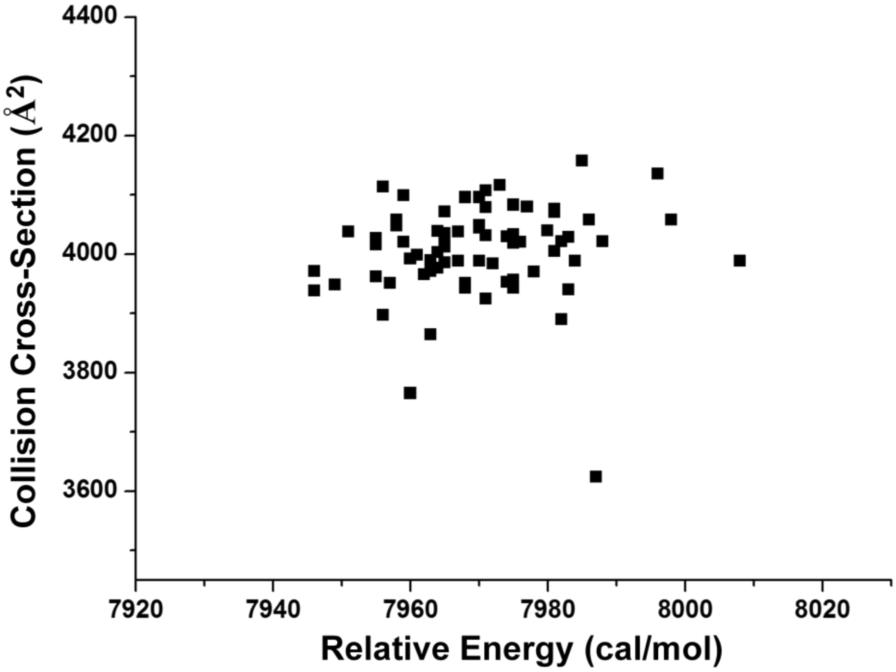

Supplementary Figure 13: Plot of collision cross-section (CCS) *vs.* relative energy for 70 candidate structures of **G3** generated by annealing simulations. CCSs were calculated by the TM method using the MOBCAL program. The average TM cross section area is 3981 Å^2.

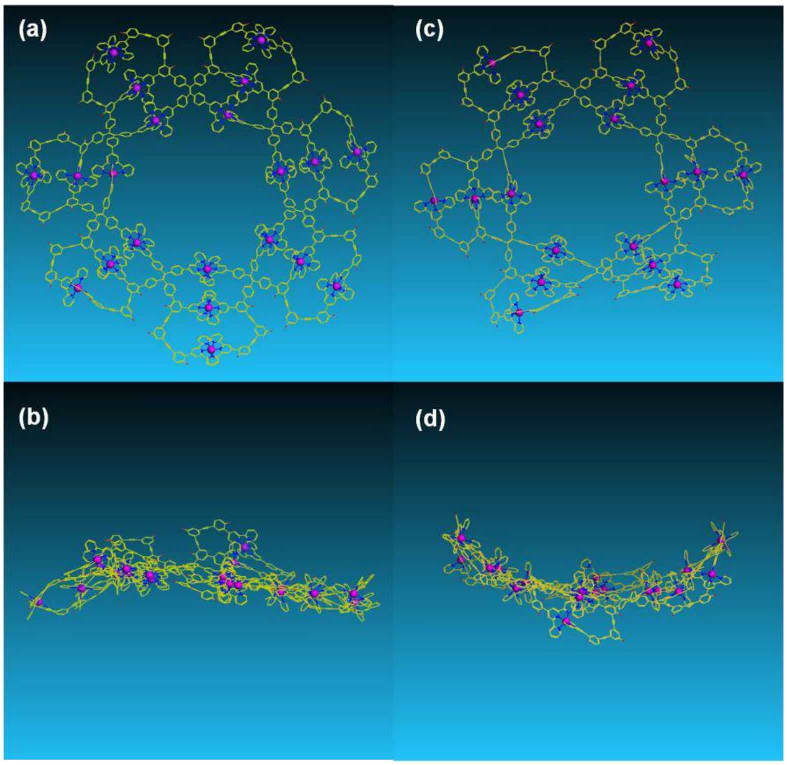

Supplementary Figure 14: The optimum structures of heptamer and hexamer of **L3** assembled with Cd^{2+}. (a) topview, (b) sideview of heptamer and (c) topview (d) sideview of hexamer.

Supplementary Table 1. Experimental and Theoretical Collision Cross Sections (CCSs).

	Drift times [ms]	CCS [Å^2]	CCS Average [Å^2]	CCS (calcd. avg) [Å^2]
**G2**	6.06 (10+)	2009.9		
	5.18 (11+)	2017.9		
	4.41 (12+)	2002.5		
	3.86 (13+)	2004.4		
	3.42 (14+)	2007.4	2040.9 ± 51.3	2065.0 ± 42
	3.09 (15+)	2022.7		
	2.87 (16+)	2062.9		
	2.65 (17+)	2087.1		
	2.54 (18+)	2153.4		
**G3**	7.28 (17+)	3793.7		
	6.62 (18+)	3802.5		
	5.95 (19+)	3776.3		
	5.51 (20+)	3801.5	3820.9 ± 40	3980.9 ± 55
	5.18 (21+)	3850.5		
	4.74 (22+)	3829.0		
	4.52 (23+)	3893.0		

^{1}H NMR, ^{13}C NMR, 2D COSY NMR, 2D NOESY NMR, 2D ROESY NMR and MALDI-TOF spectra

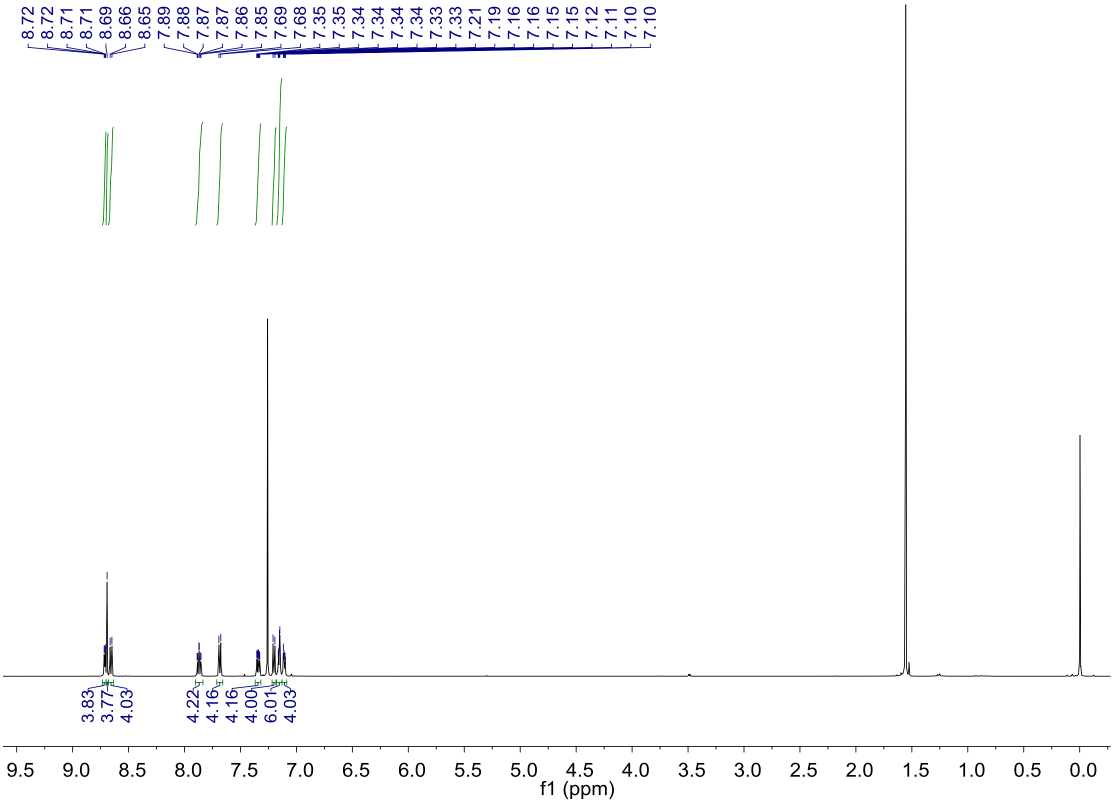

Supplementary Figure 15: ^{1}H NMR (500 MHz, $CDCl_3$, 300 K) spectrum of ligand **L1.**

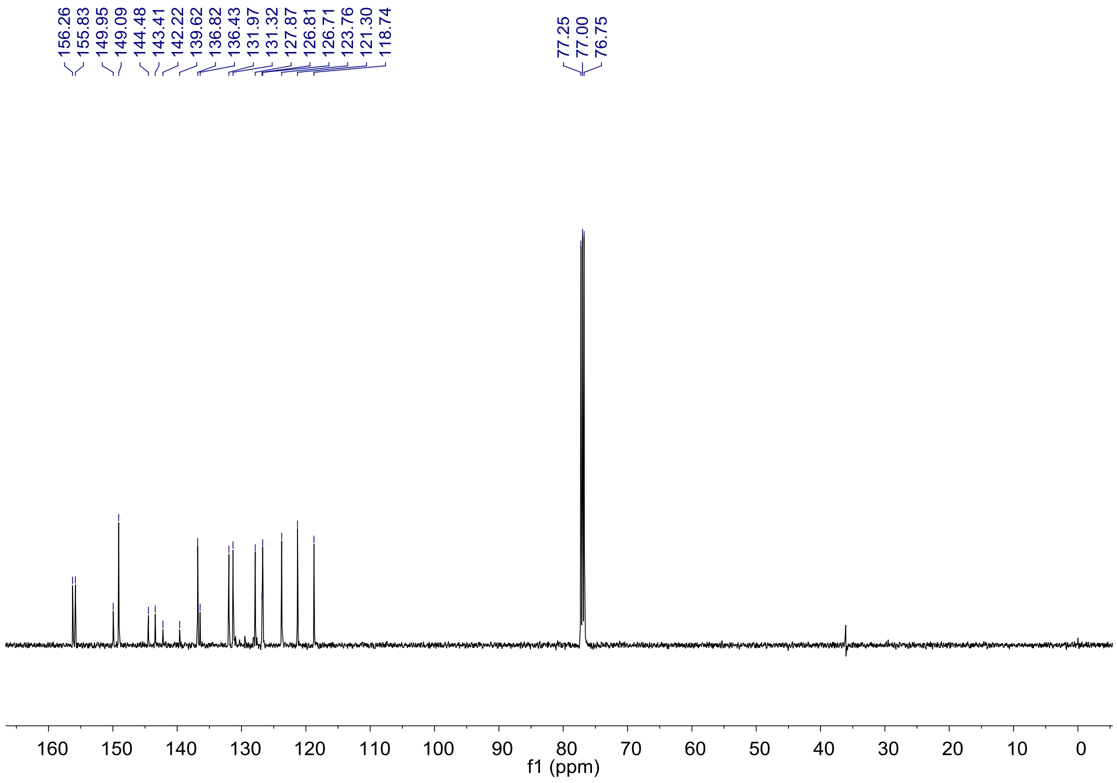

Supplementary Figure 16: ^{13}C NMR (125 MHz, $CDCl_3$, 300 K) spectrum of ligand **L1.**

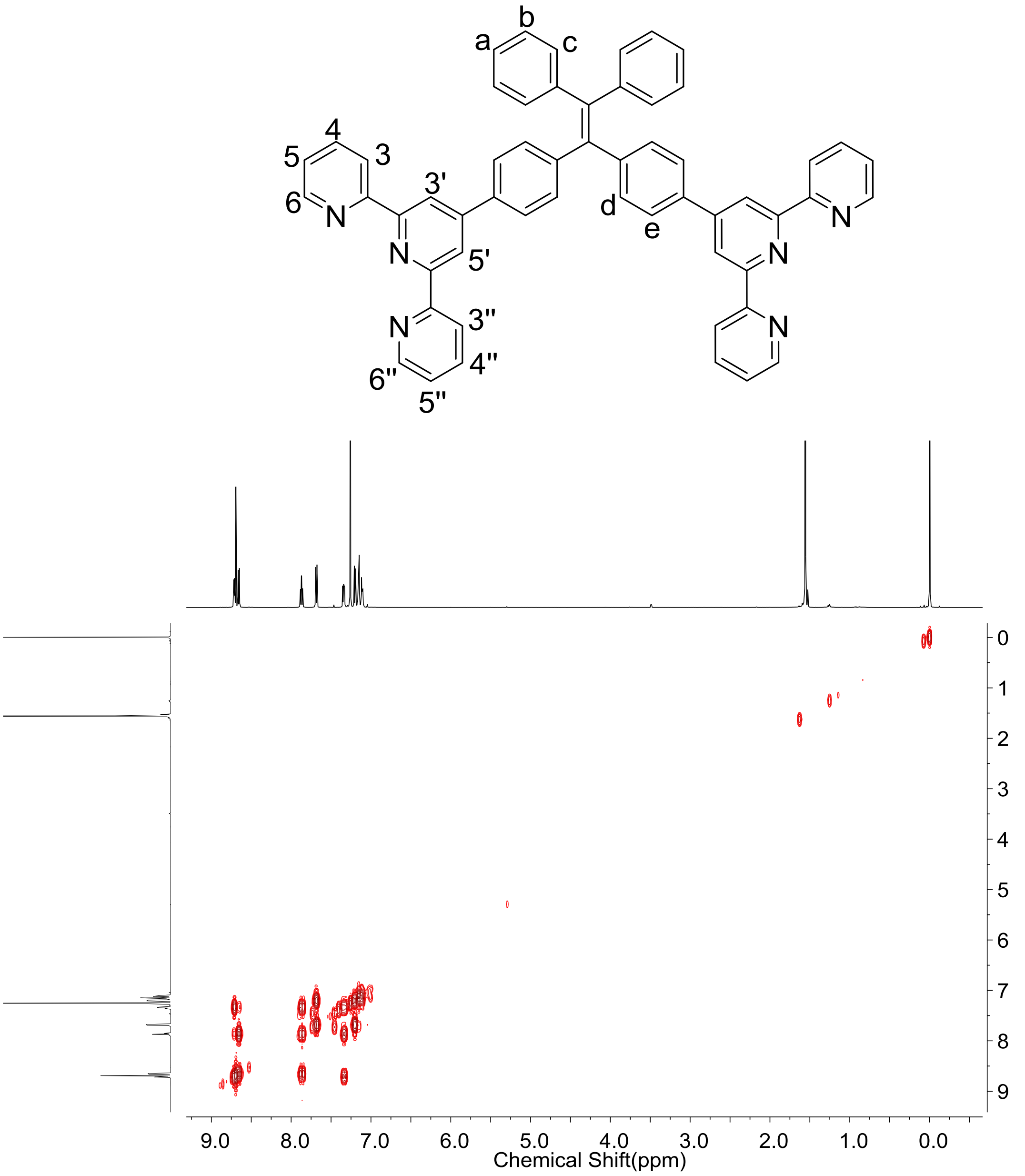

Supplementary Figure 17: 2D COSY NMR (500 MHz, $CDCl_3$, 300 K) spectrum of ligand **L1**

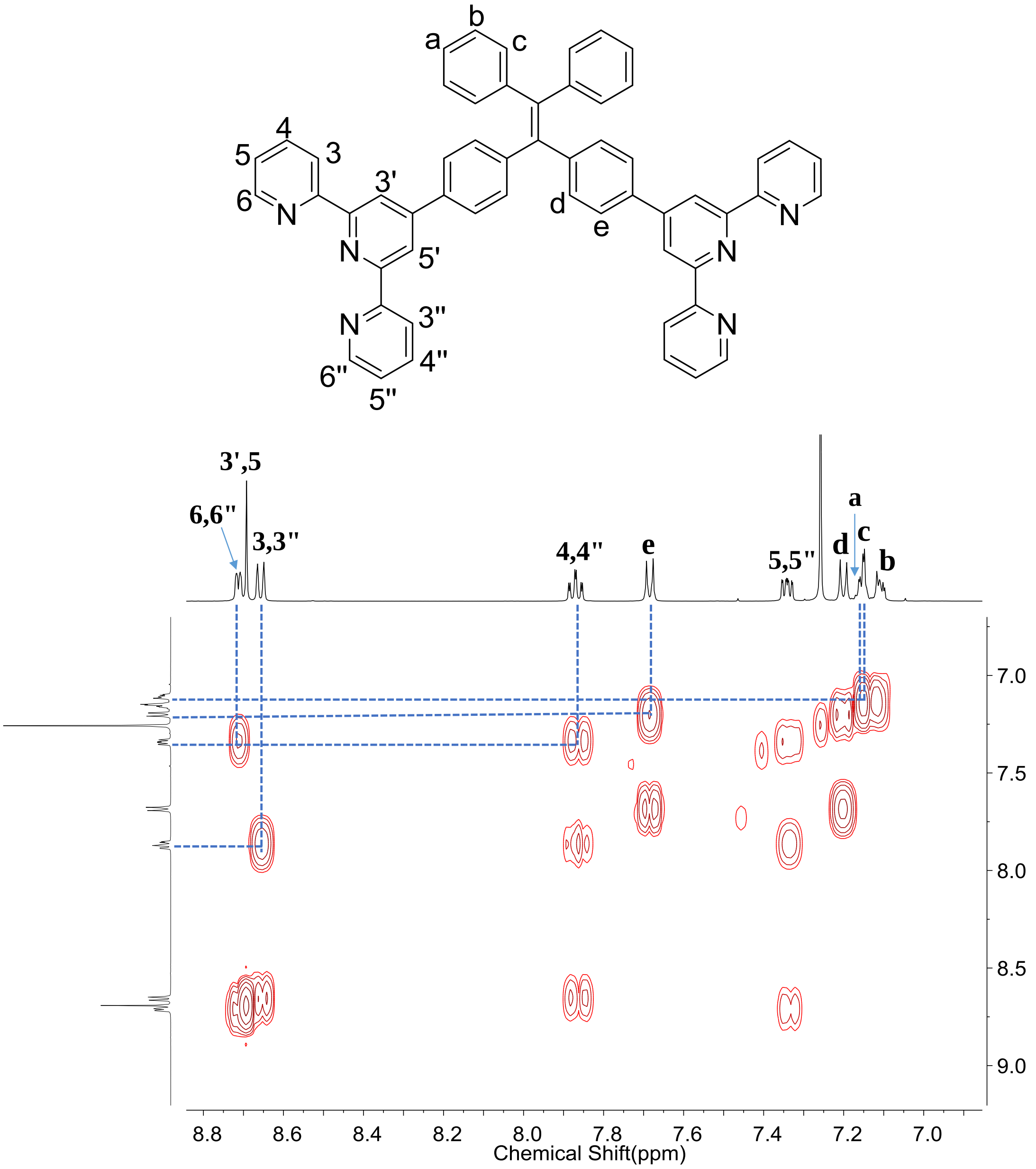

Supplementary Figure 18: 2D COSY NMR (500 MHz, $CDCl_3$, 300 K) spectrum of ligand **L1** (aromatic region).

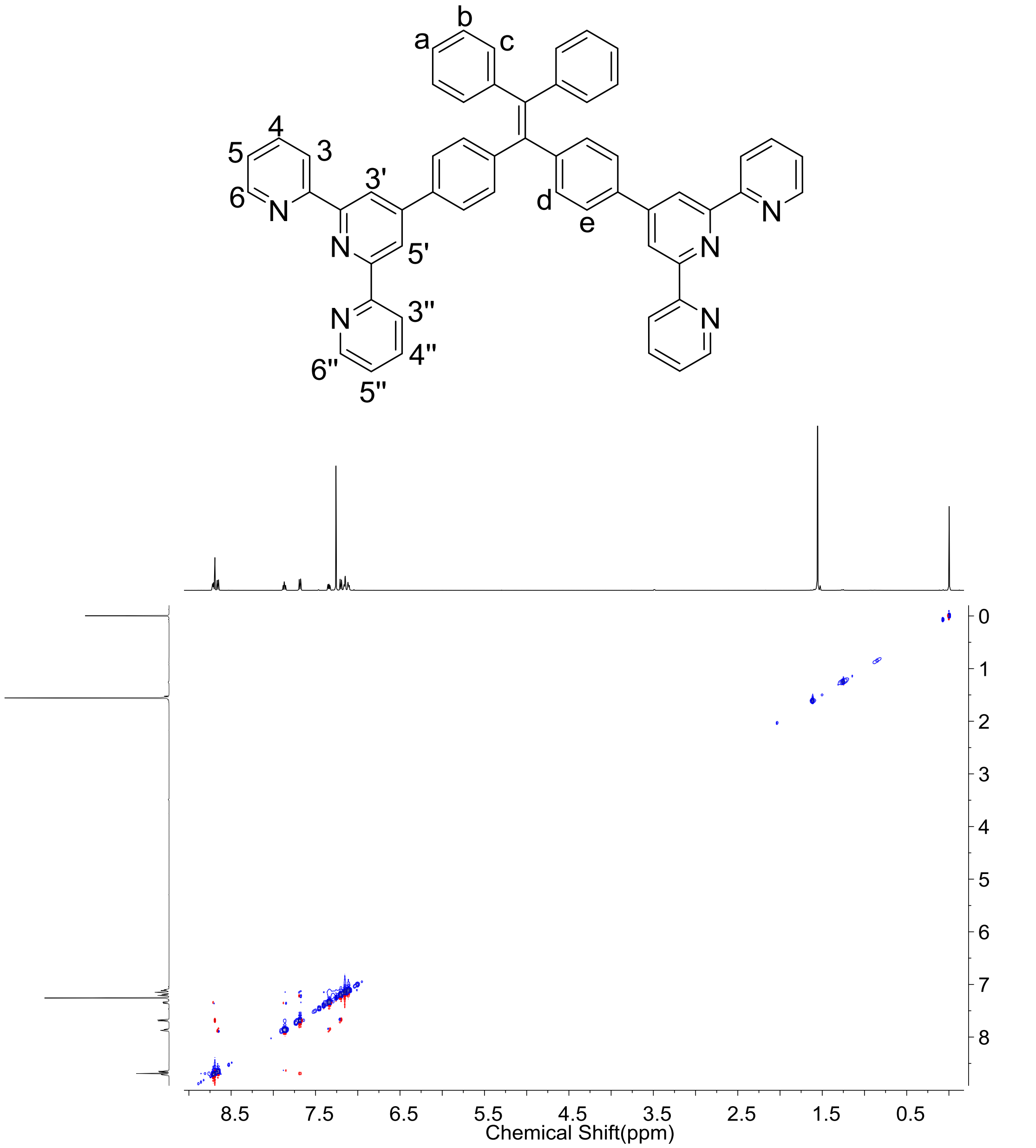

Supplementary Figure 19: 2D NOESY NMR (500 MHz, $CDCl_3$, 300 K) spectrum of **L1**.

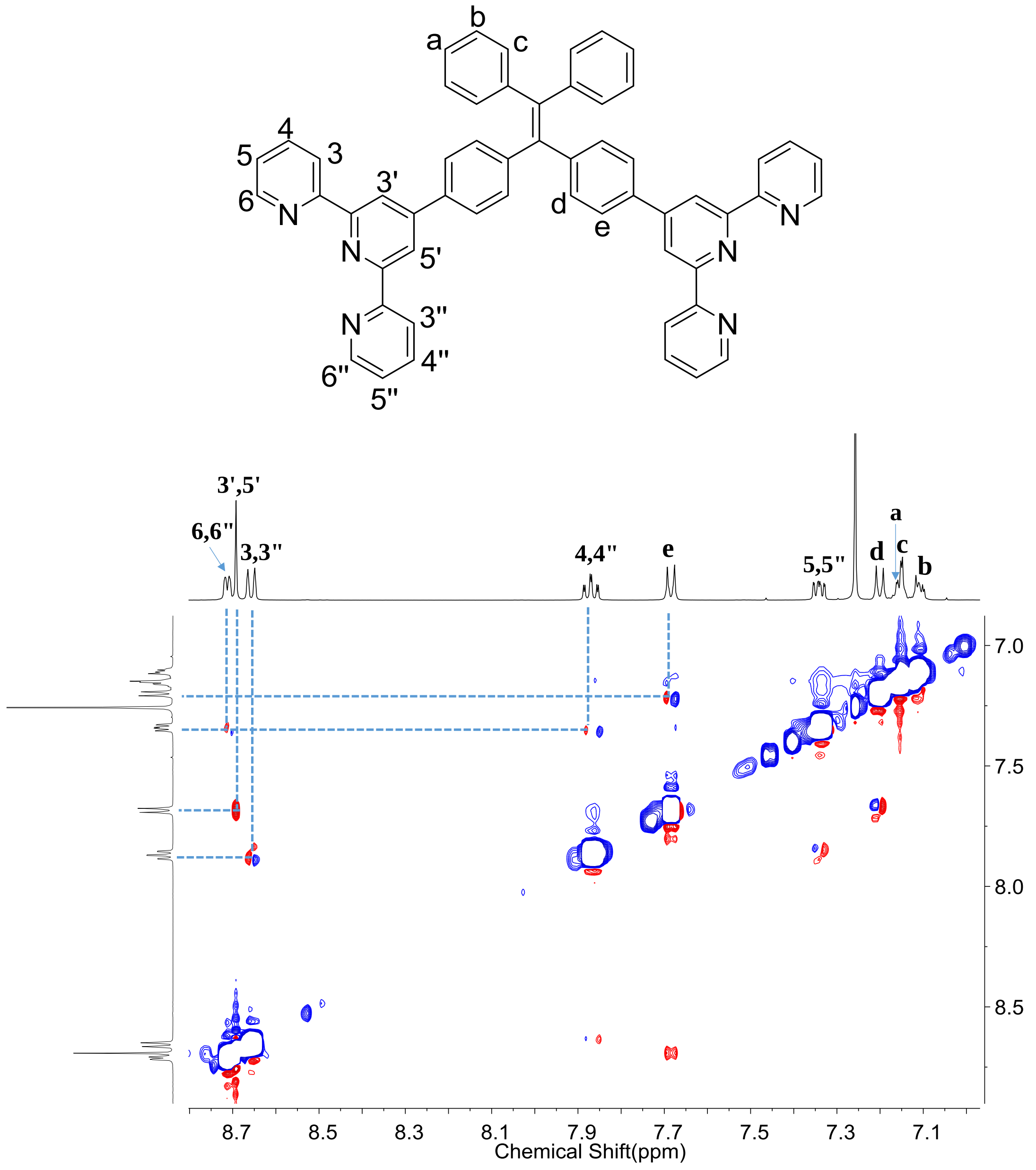

Supplementary Figure 20: 2D NOESY NMR (500 MHz, $CDCl_3$, 300 K) spectrum of **L1** (aromatic region).

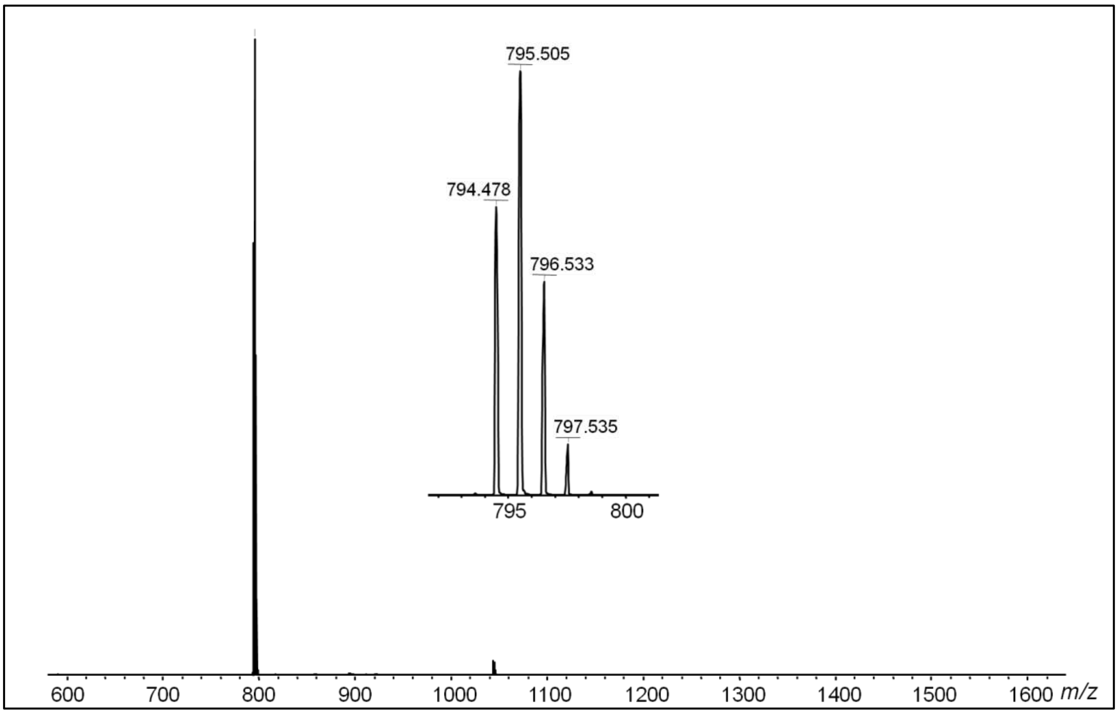

Supplementary Figure 21: MALDI-TOF mass spectrum of ligand **L1**.

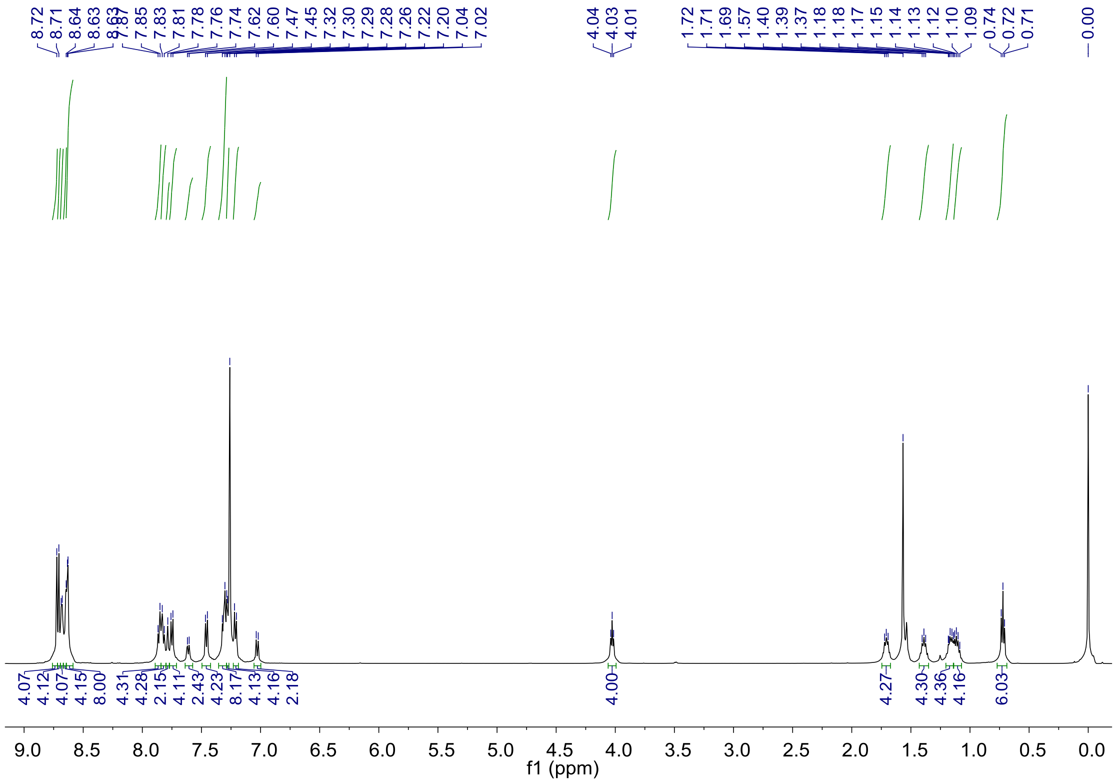

Supplementary Figure 22: ^{1}H NMR (500 MHz, $CDCl_3$, 300 K) spectrum of ligand **L2.**

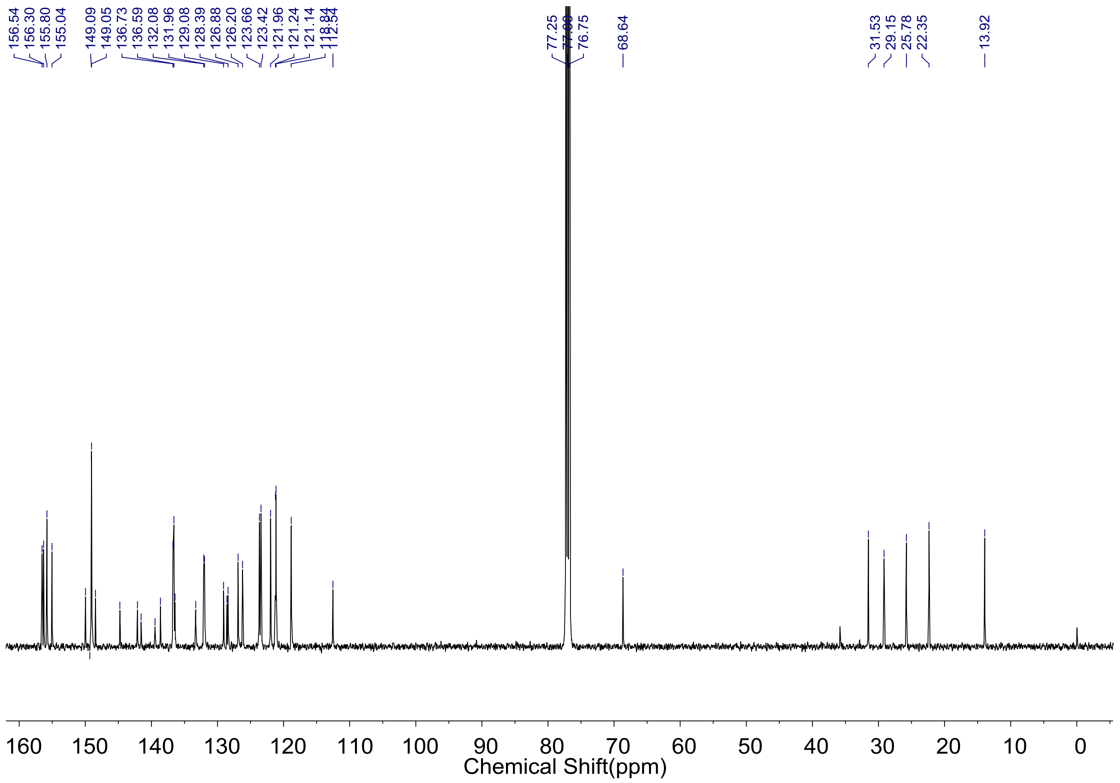

Supplementary Figure 23: ^{13}C NMR (100 MHz, $CDCl_3$, 300 K) spectrum of ligand **L2.**

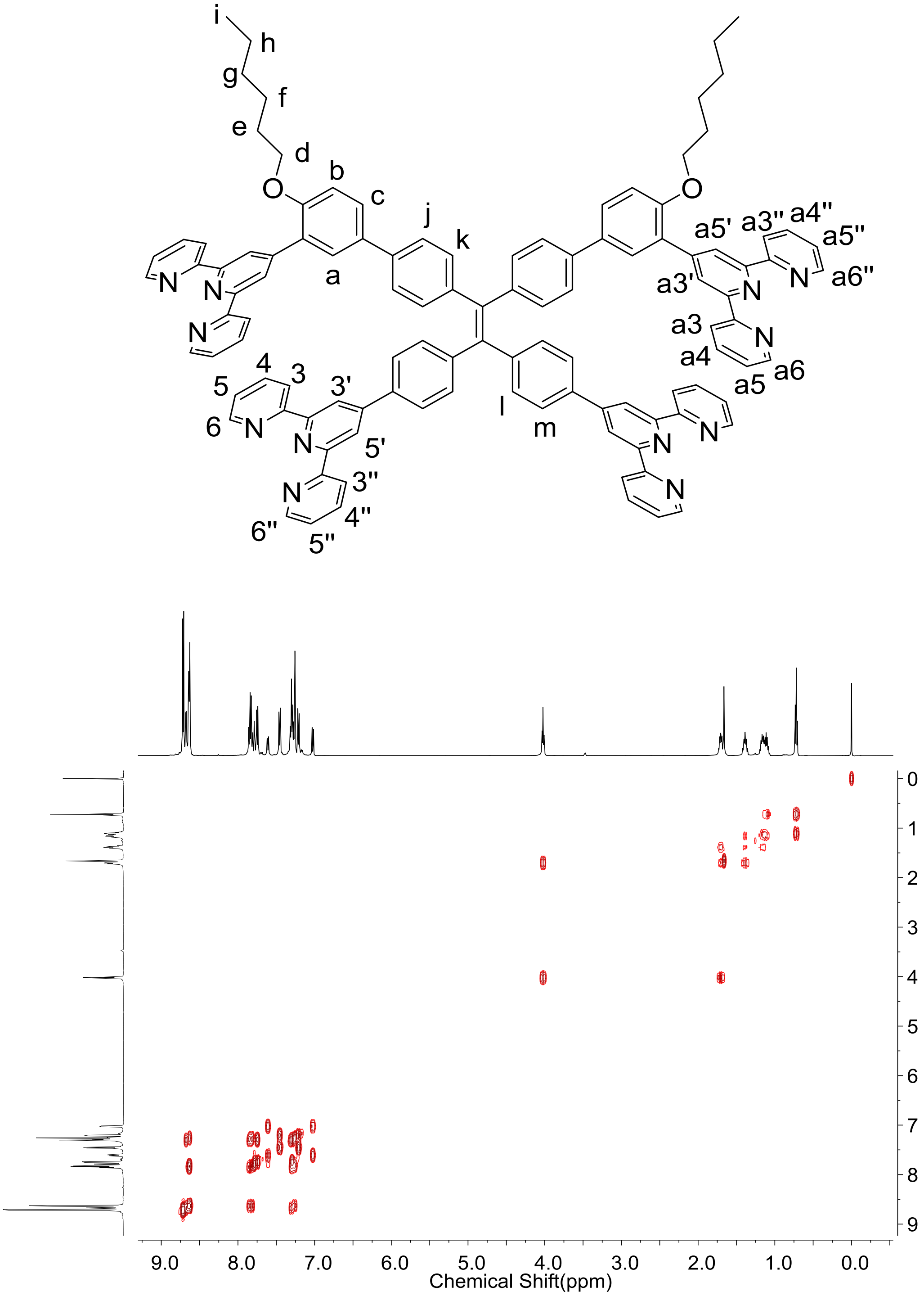

Supplementary Figure 24: 2D COSY NMR (500 MHz, $CDCl_3$, 300 K) spectrum of ligand **L2.**

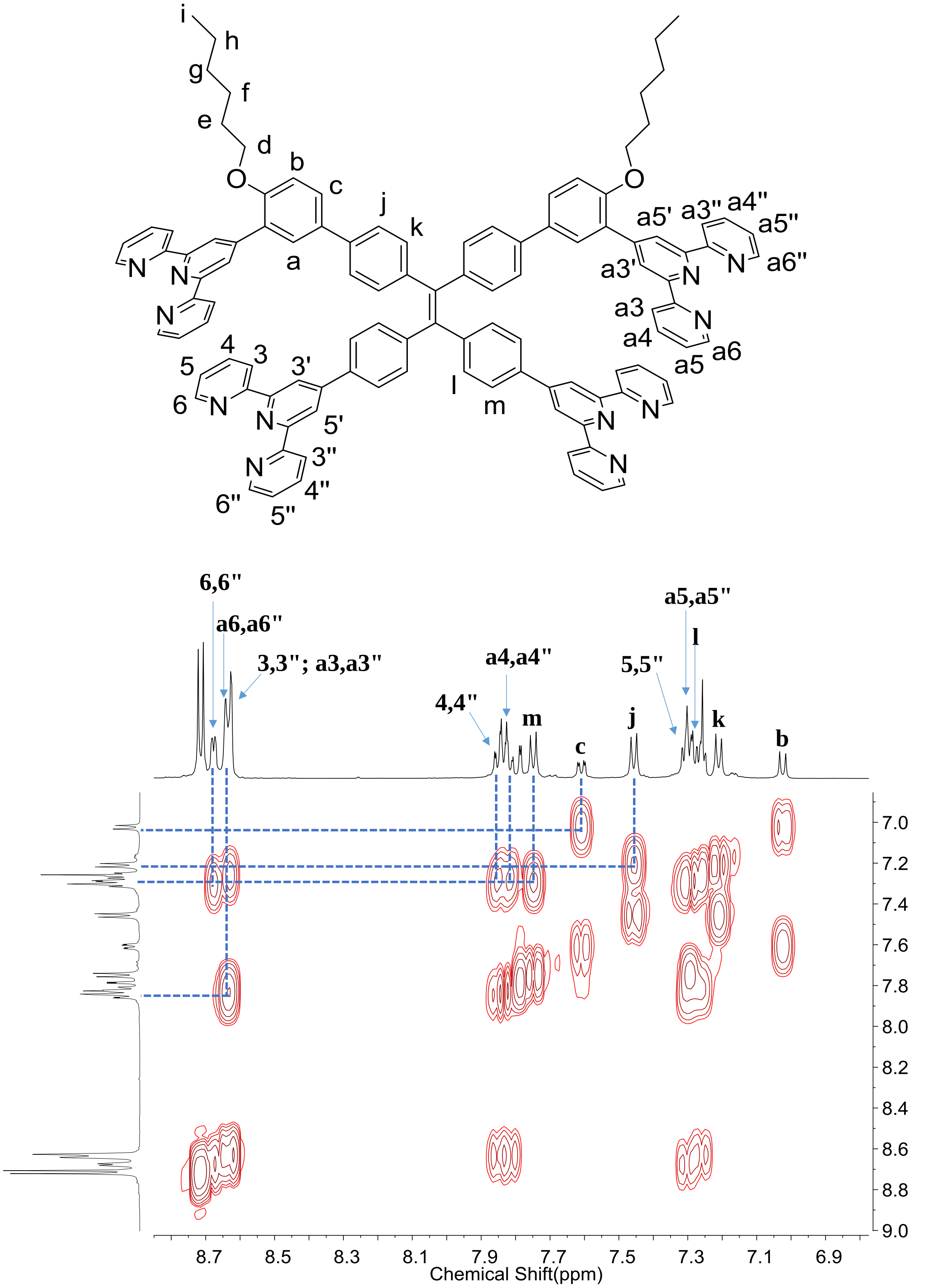

Supplementary Figure 25: 2D COSY NMR (500 MHz, $CDCl_3$, 300 K) spectrum of ligand **L2** (aromatic region).

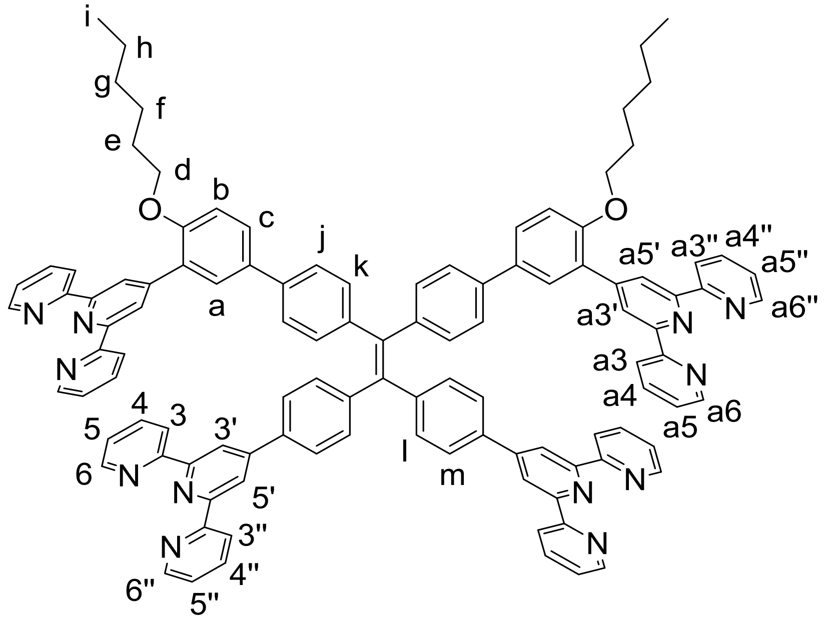

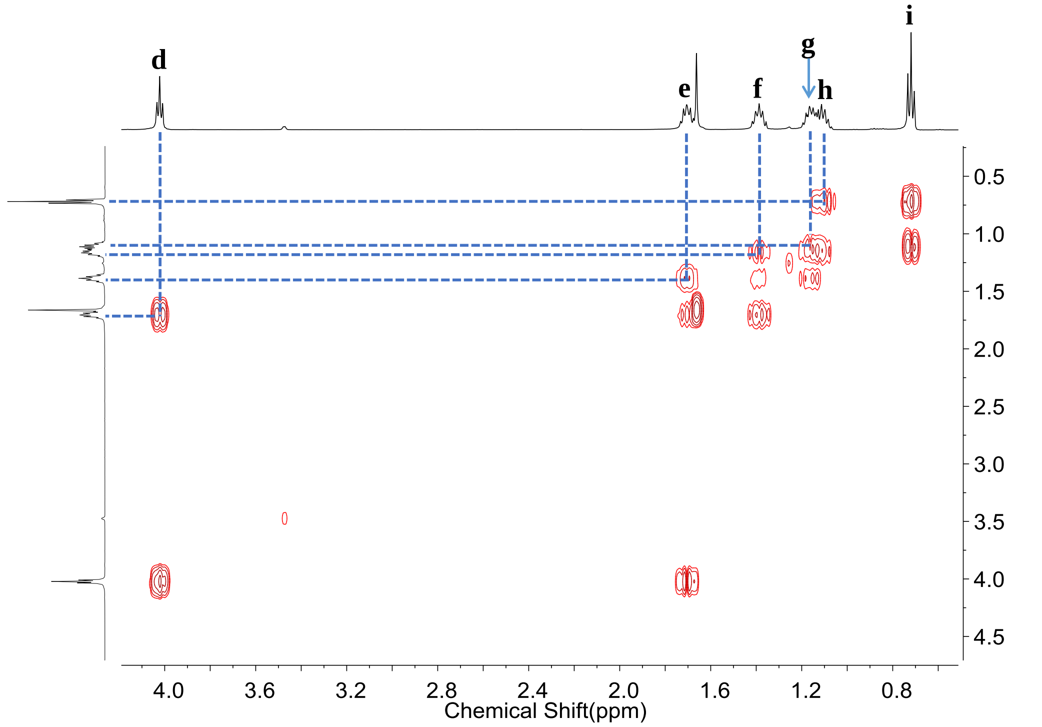

Supplementary Figure 26: 2D COSY NMR (500 MHz, $CDCl_3$, 300 K) spectrum of ligand **L2** (aliphatic region).

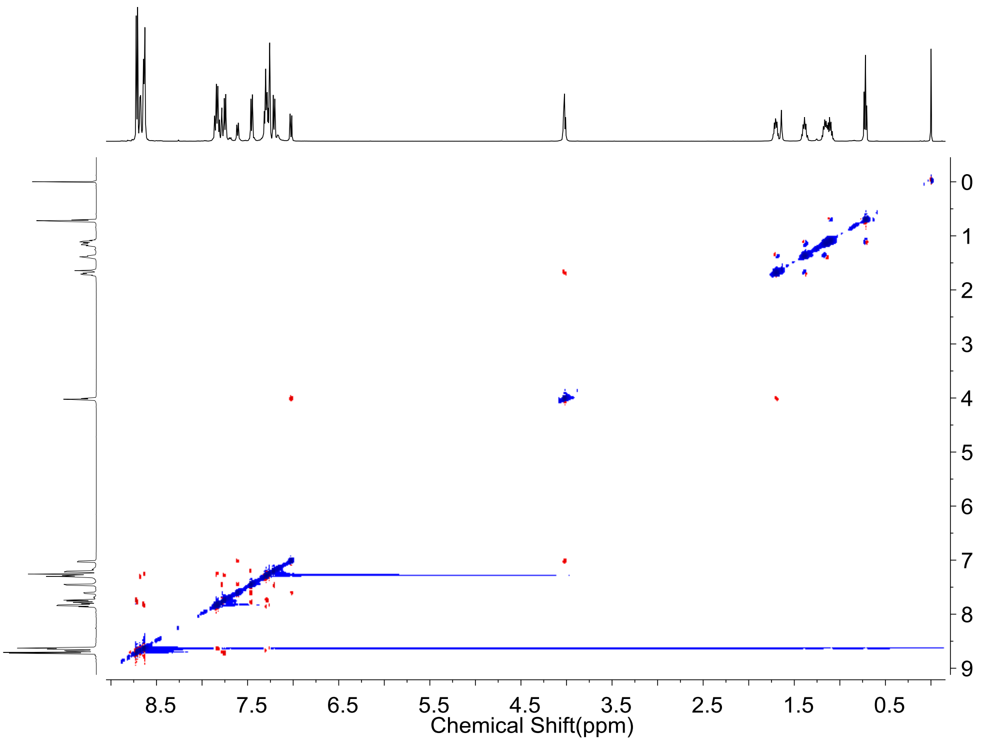

Supplementary Figure 27: 2D ROESY NMR (500 MHz, $CDCl_3$, 300 K) spectrum of ligand **L2.**

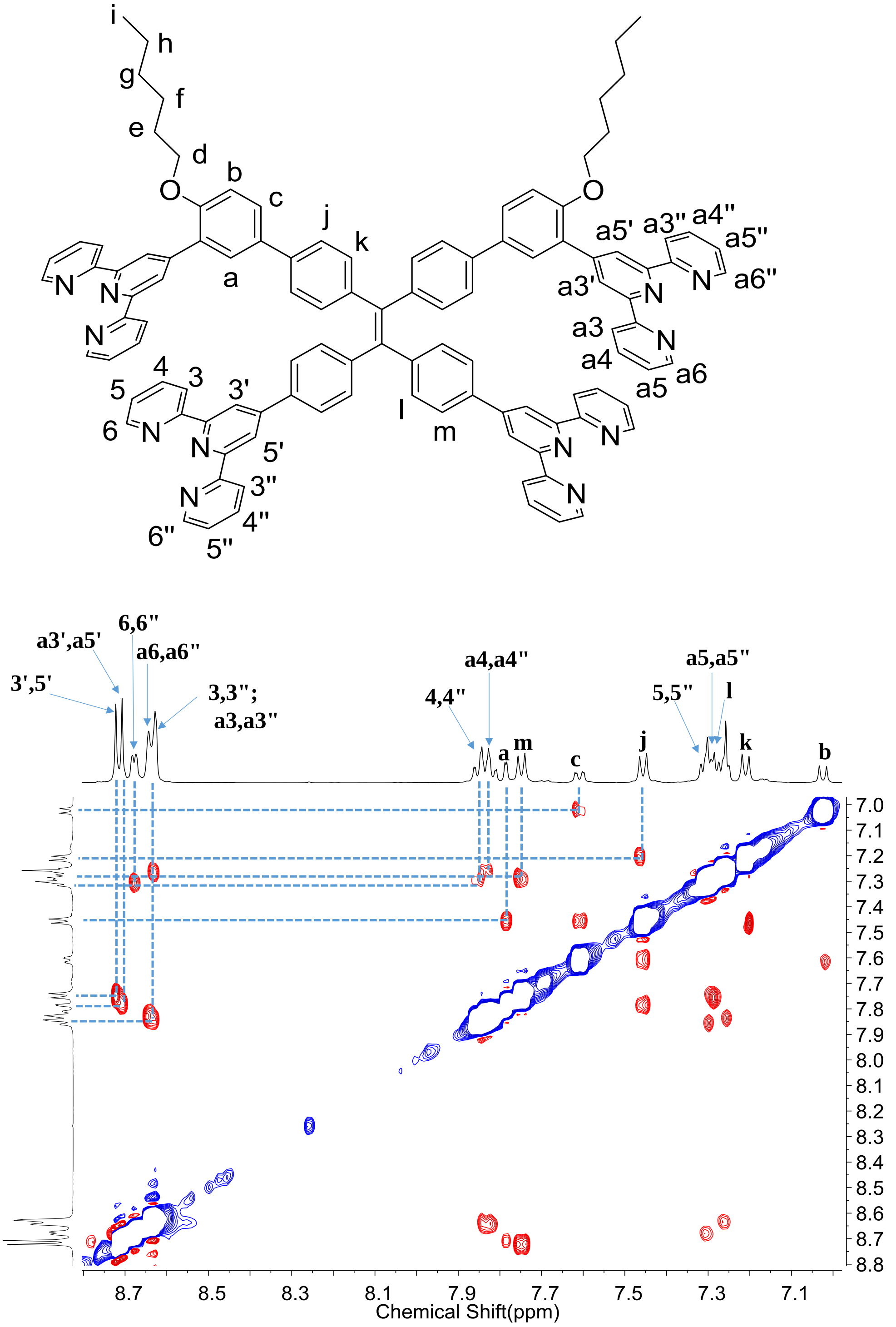

Supplementary Figure 28: 2D ROESY NMR (500 MHz, $CDCl_3$, 300 K) spectrum of ligand **L2** (aromatic region).

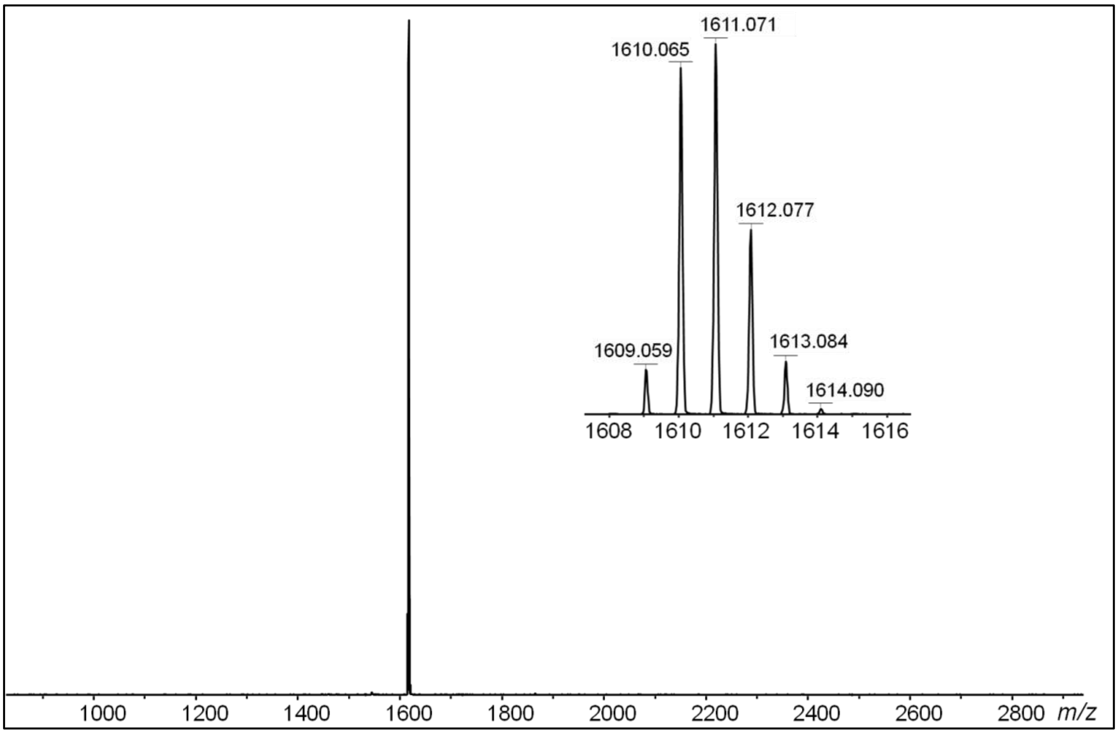

Supplementary Figure 29: MALDI-TOF mass spectrum of ligand **L2**.

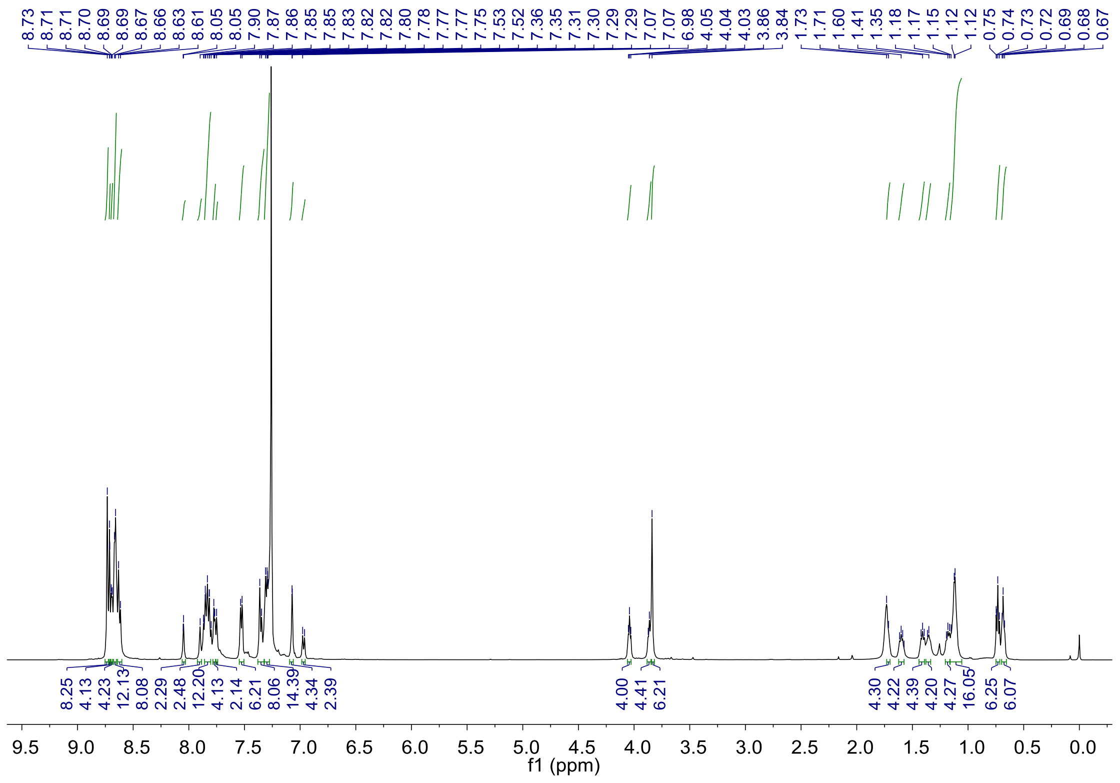

Supplementary Figure 30: ^{1}H NMR (500 MHz, $CDCl_3$, 300 K) spectrum of ligand **L3.**

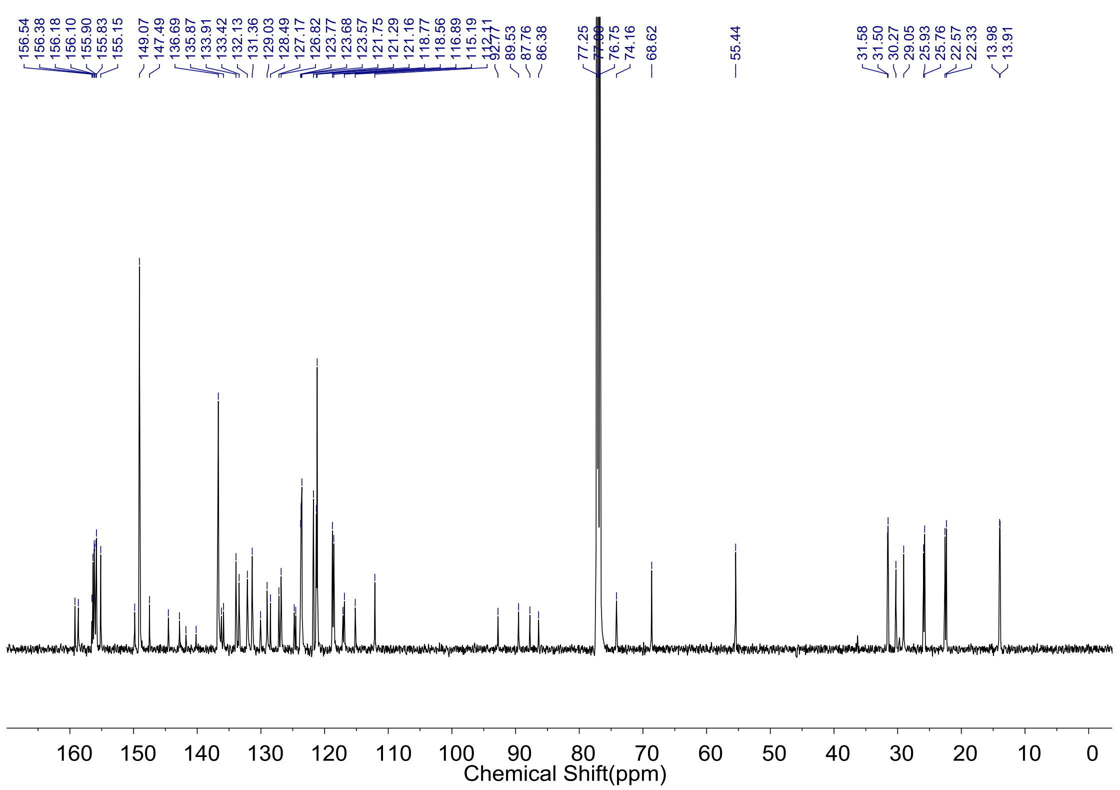

Supplementary Figure 31: ^{13}C NMR (125 MHz, $CDCl_3$, 300 K) spectrum of ligand **L3.**

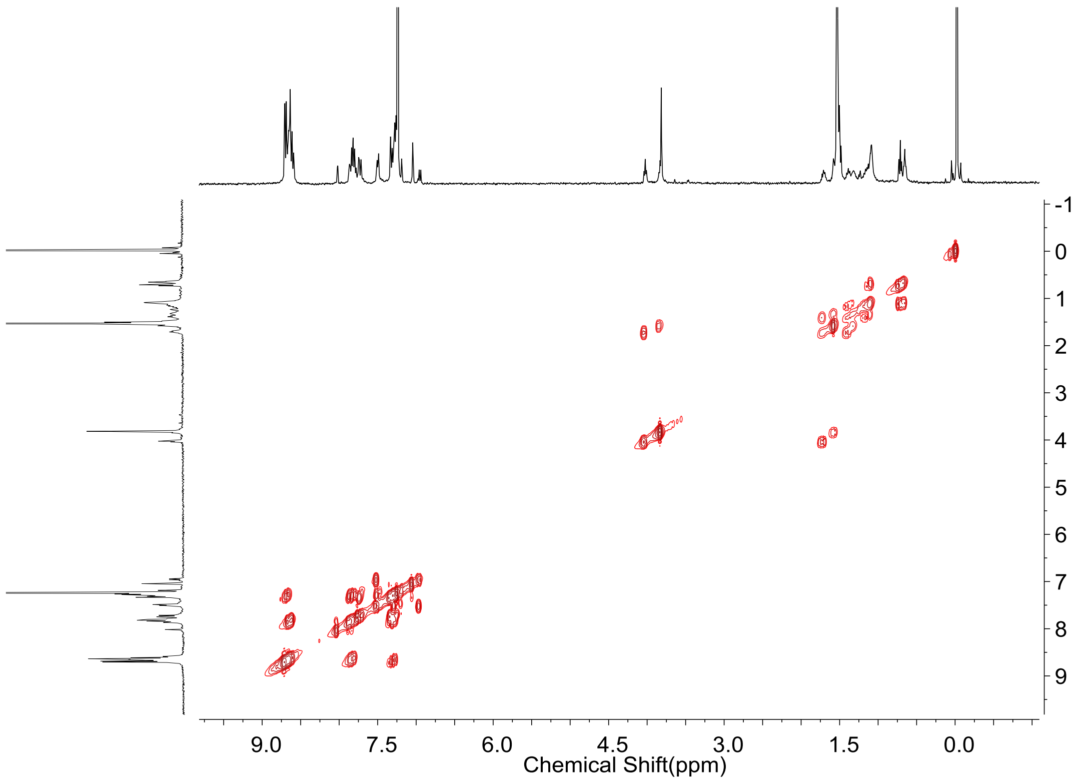

Supplementary Figure 32: 2D COSY NMR (500 MHz, $CDCl_3$, 300 K) spectrum of ligand **L3.**

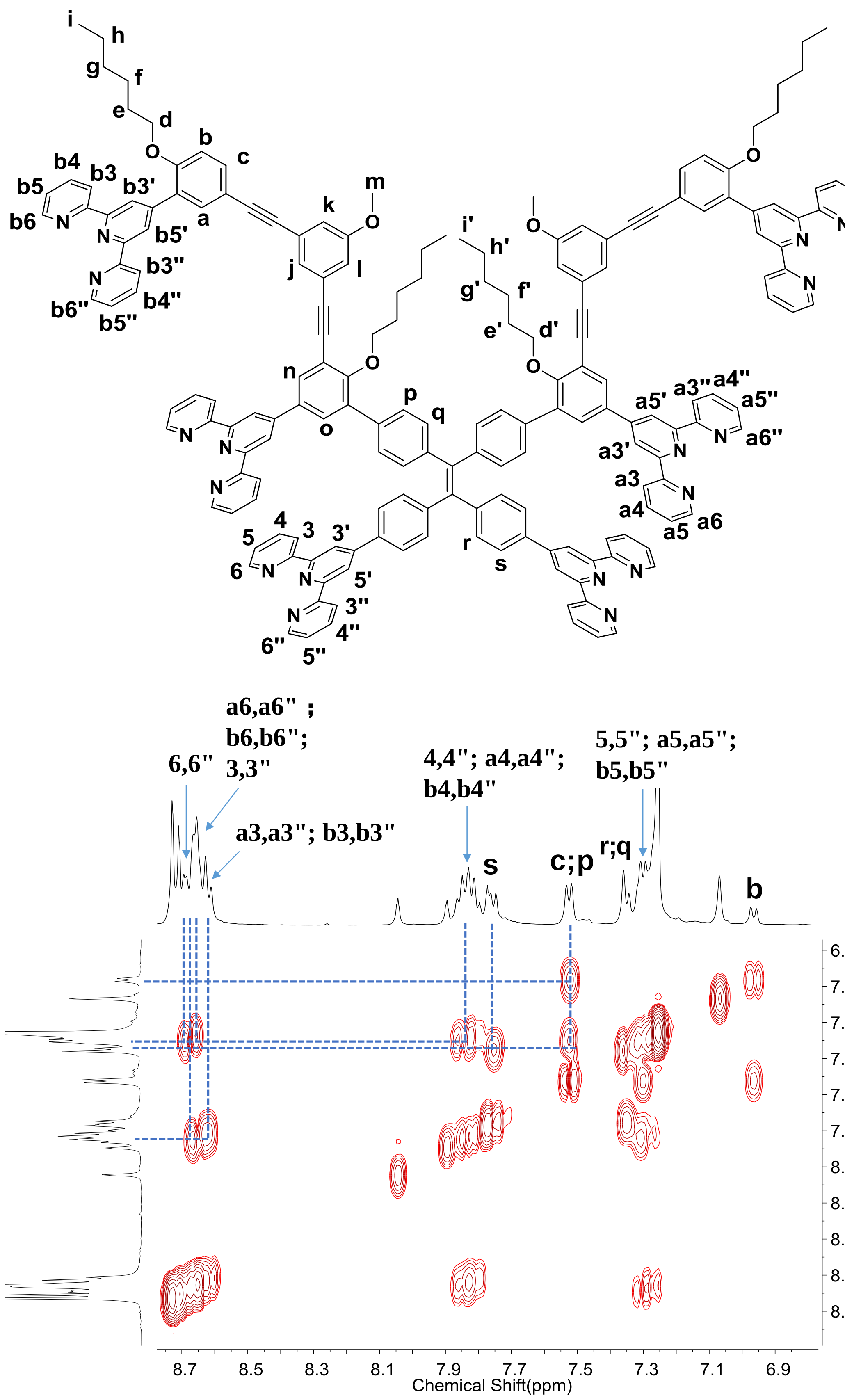

Supplementary Figure 33: 2D COSY NMR (500 MHz, $CDCl_3$, 300 K) spectrum of ligand **L3** (aromatic region).

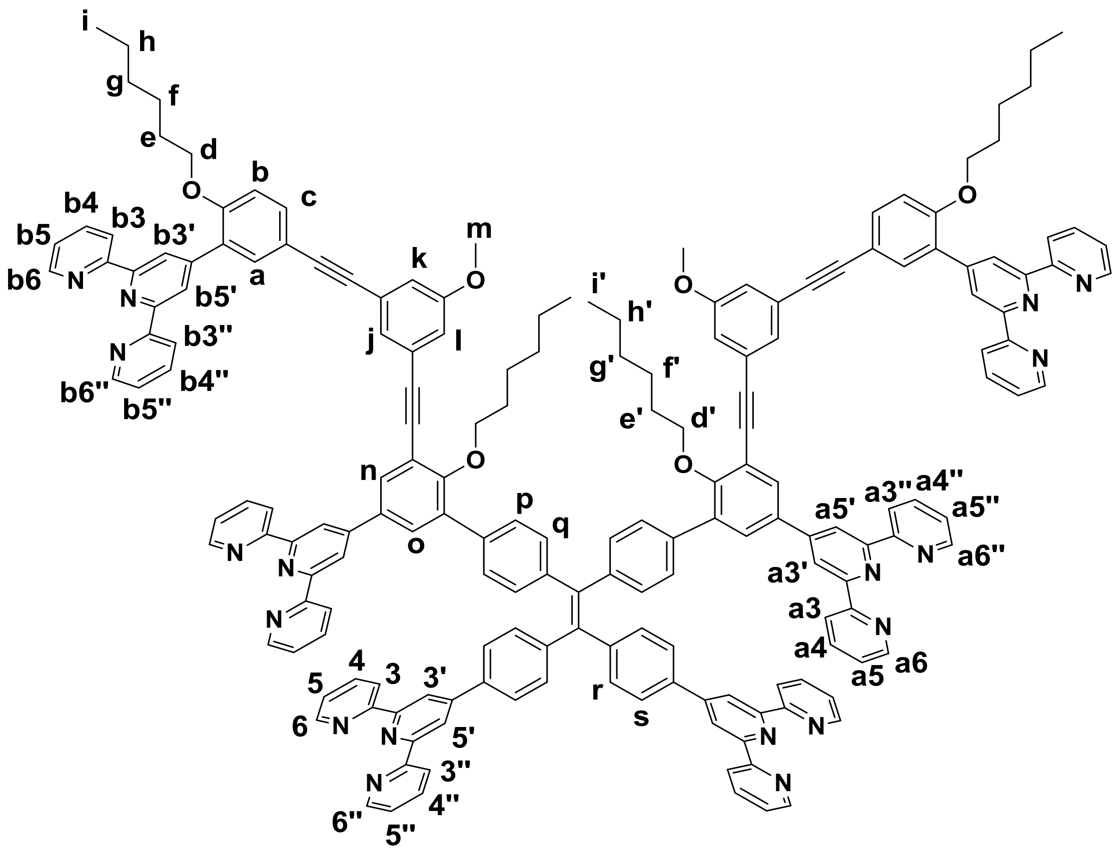

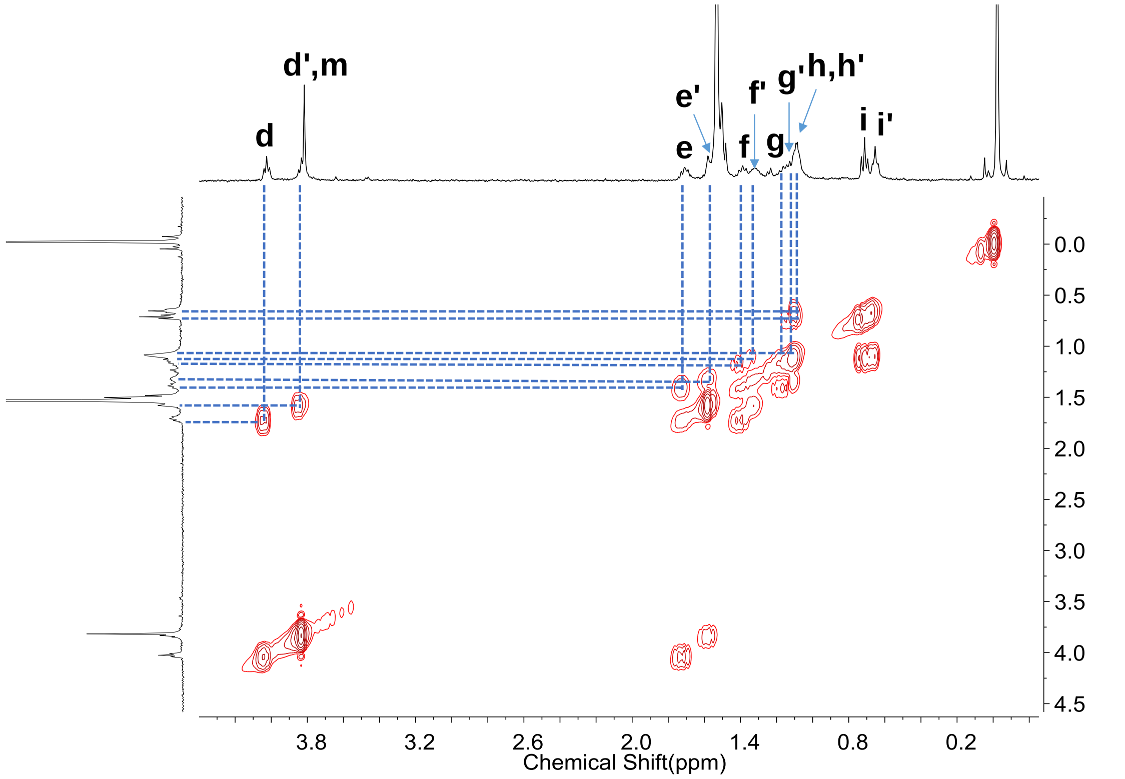

Supplementary Figure 34: 2D COSY NMR (500 MHz, $CDCl_3$, 300 K) spectrum of ligand **L3** (aliphatic region).

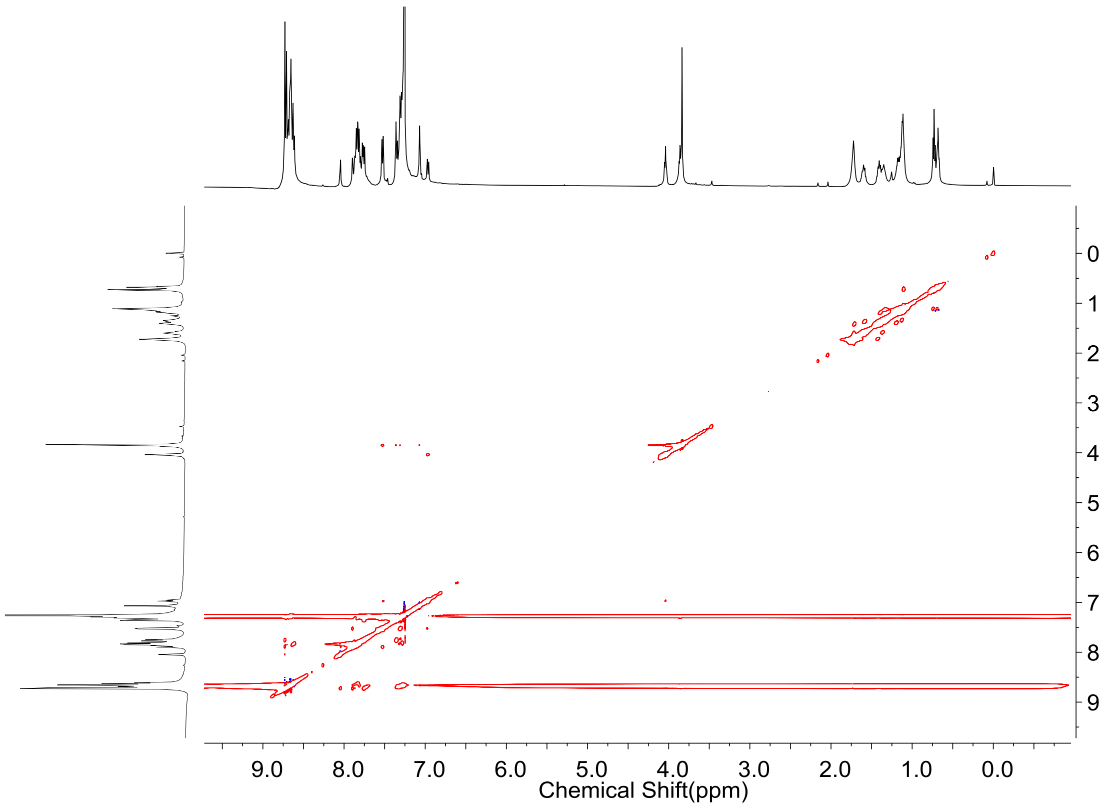

Supplementary Figure 35: 2D NOESY NMR (500 MHz, $CDCl_3$, 300 K) spectrum of **L3**.

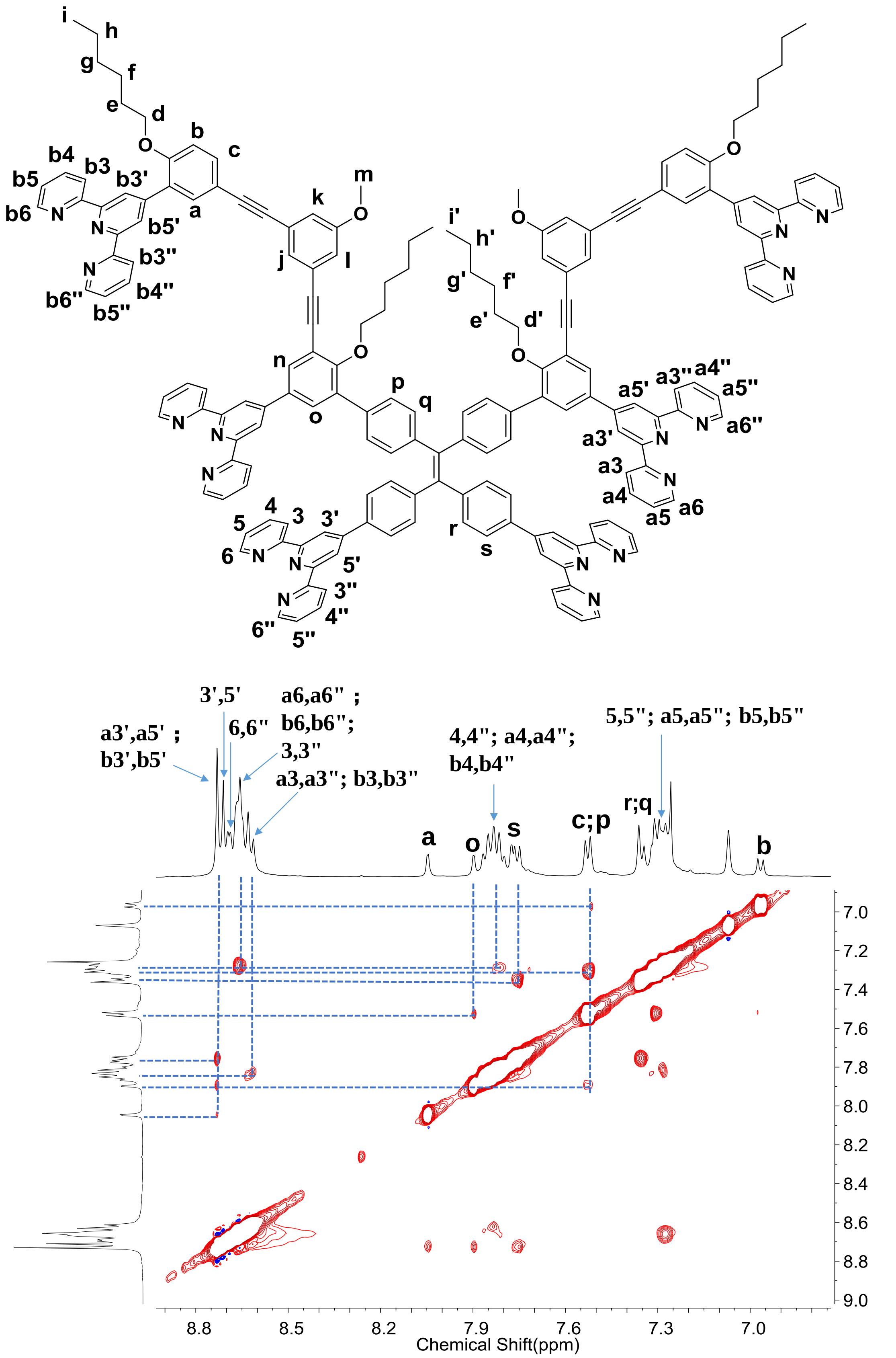

Supplementary Figure 36: 2D NOESY NMR (500 MHz, $CDCl_3$, 300 K) spectrum of **L3** (aromatic region).

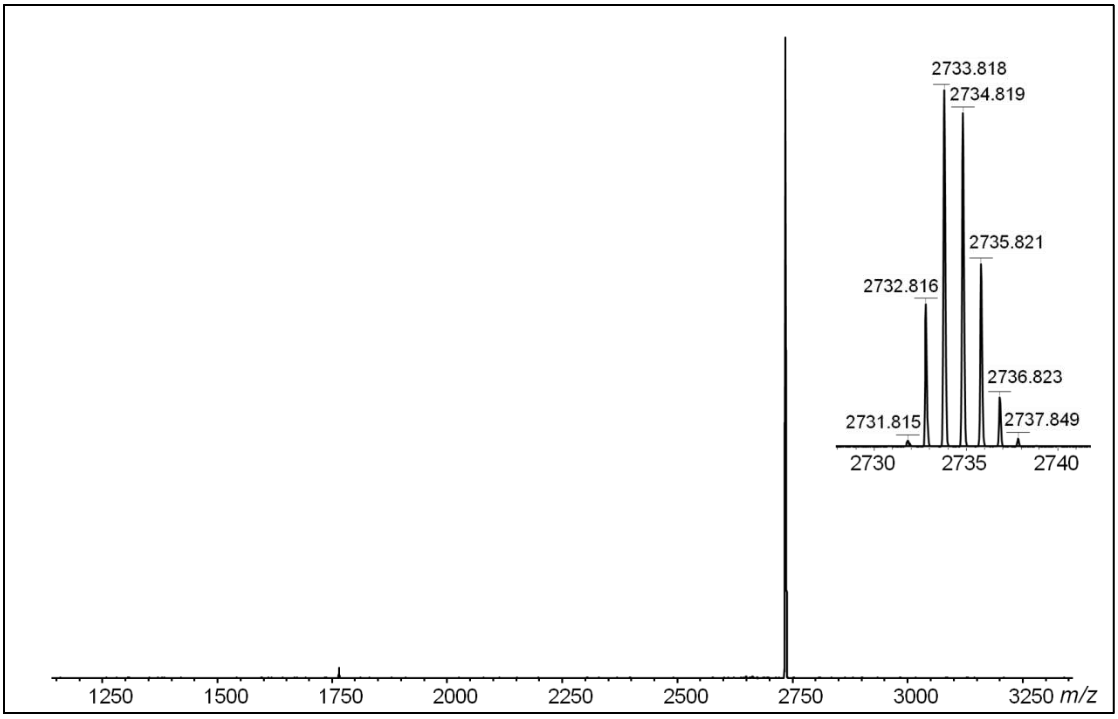

Supplementary Figure 37: MALDI-TOF mass spectrum of ligand **L3**.

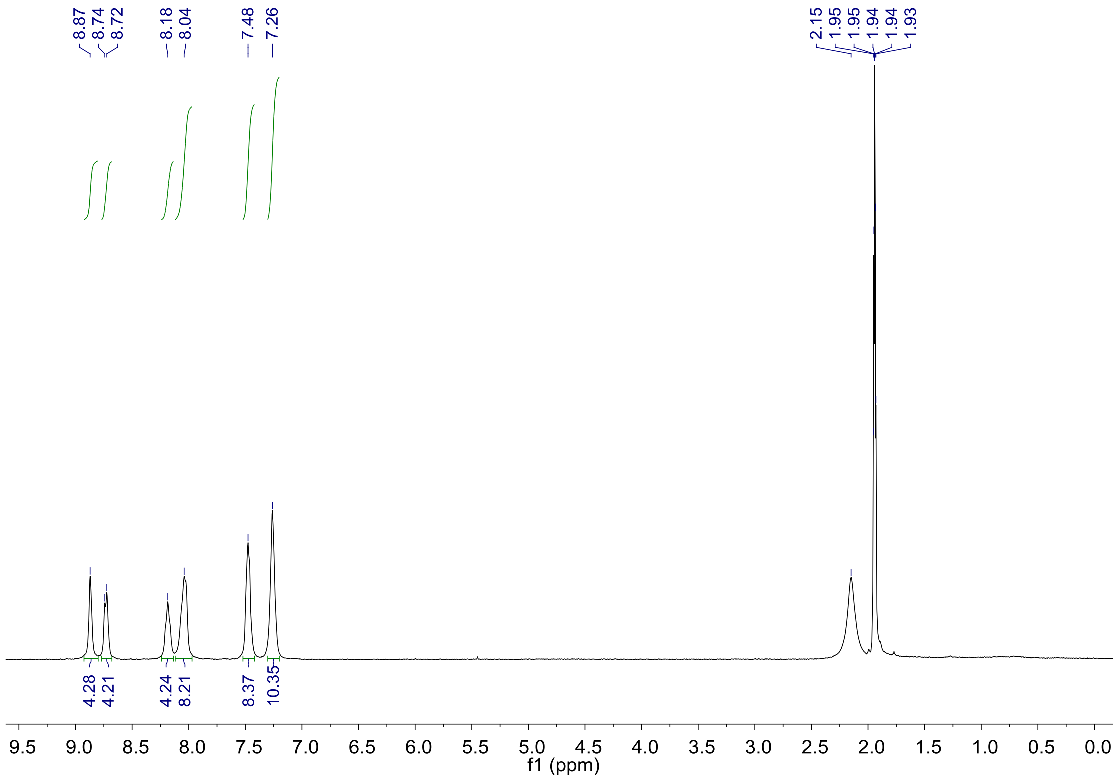

Supplementary Figure 38: ^{1}H NMR (400 MHz, CD_3CN, 300 K) spectrum of **G1.**

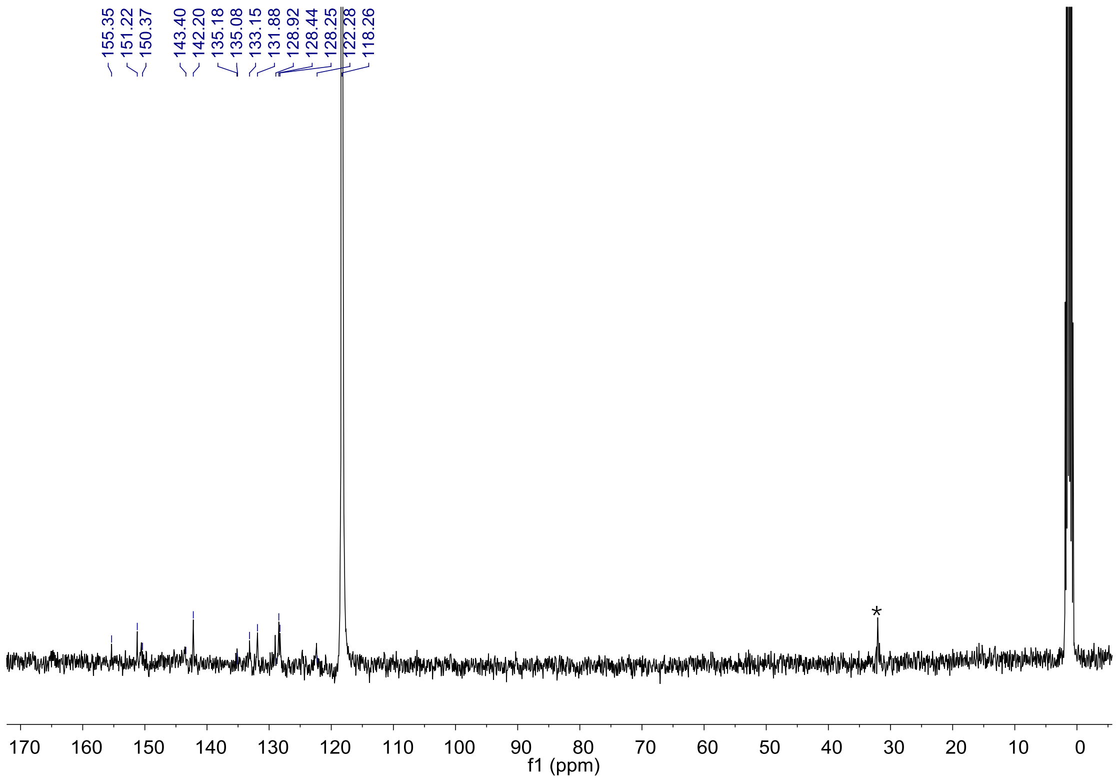

Supplementary Figure 39: ^{13}C NMR (100 MHz, CD_3CN, 300 K) spectrum of **G1.** The * peak is the acetone peak.

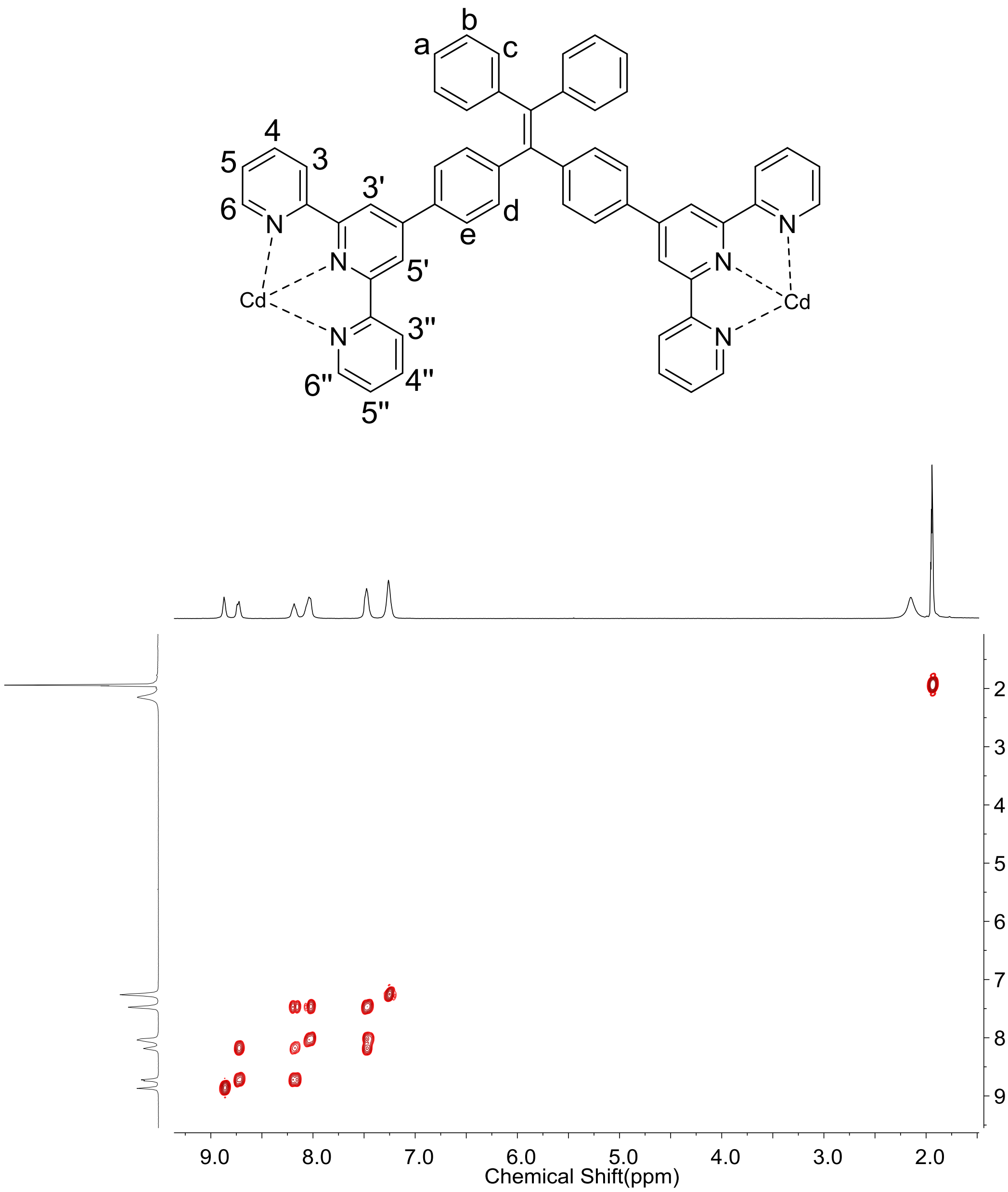

Supplementary Figure 40: 2D COSY NMR (400 MHz, CD_3CN, 300 K) spectrum of **G1**.

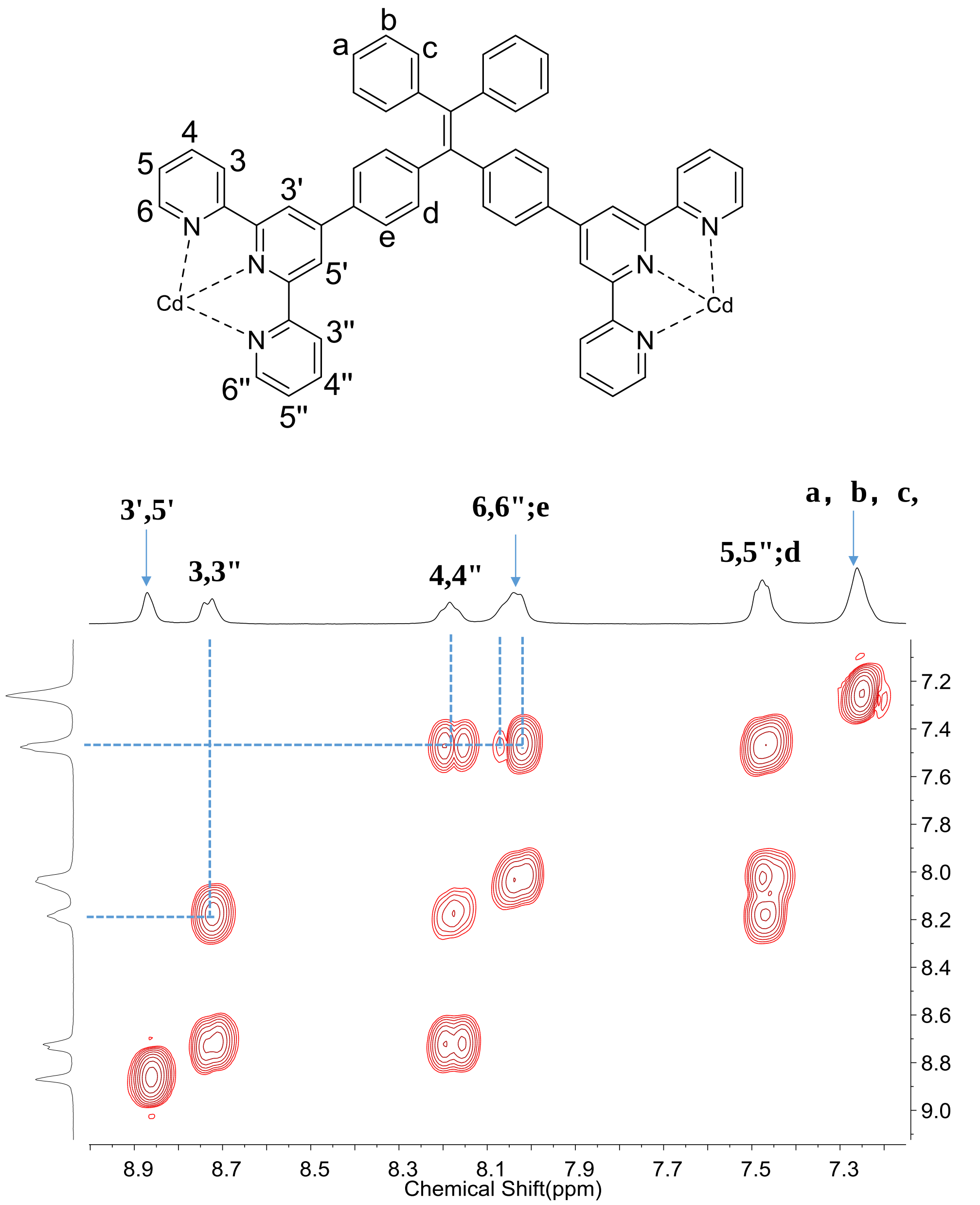

Supplementary Figure 41: 2D COSY NMR (400 MHz, CD_3CN, 300 K) spectrum of **G1** (aromatic region).

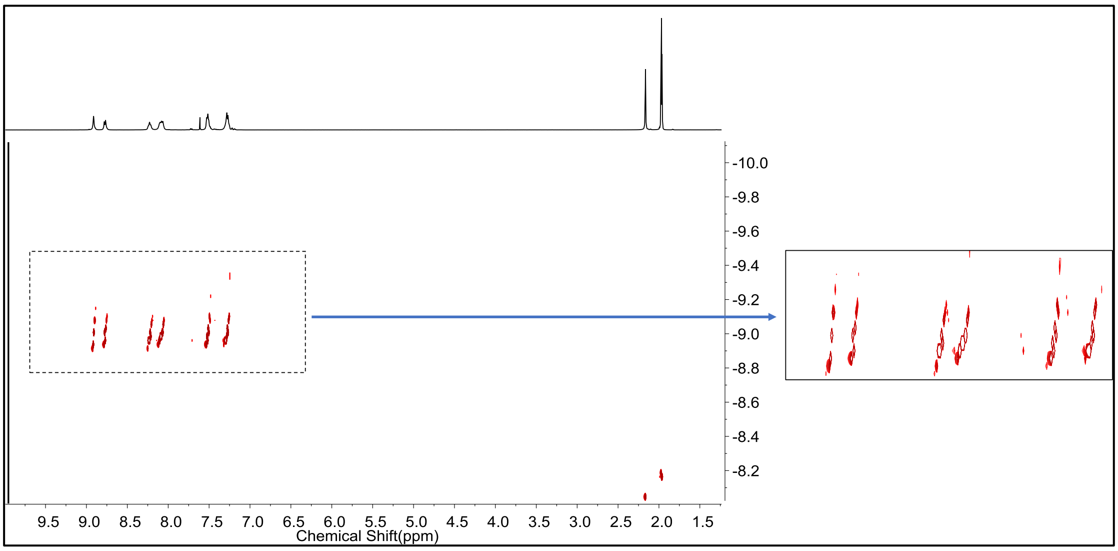

Supplementary Figure 42: 2D DOSY (500 MHz, CD_3CN, 300 K) spectrum of **G1**.

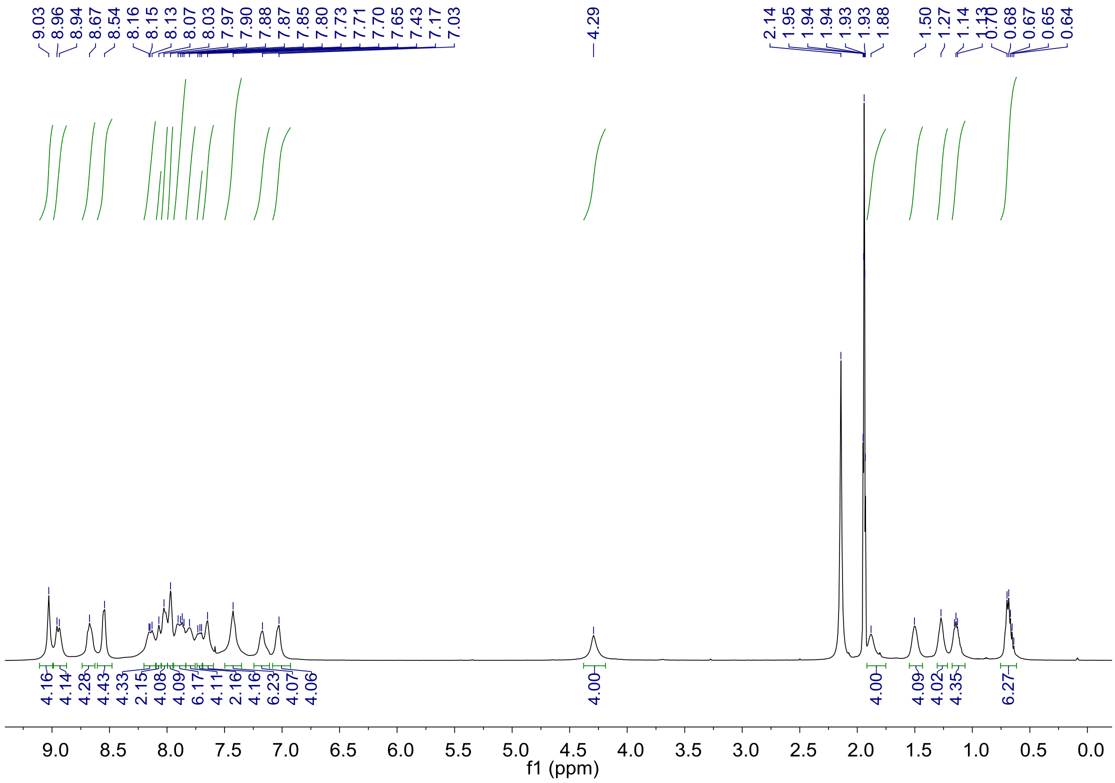

Supplementary Figure 43: ^{1}H NMR (500 MHz, CD_3CN, 300 K) spectrum of **G2.**

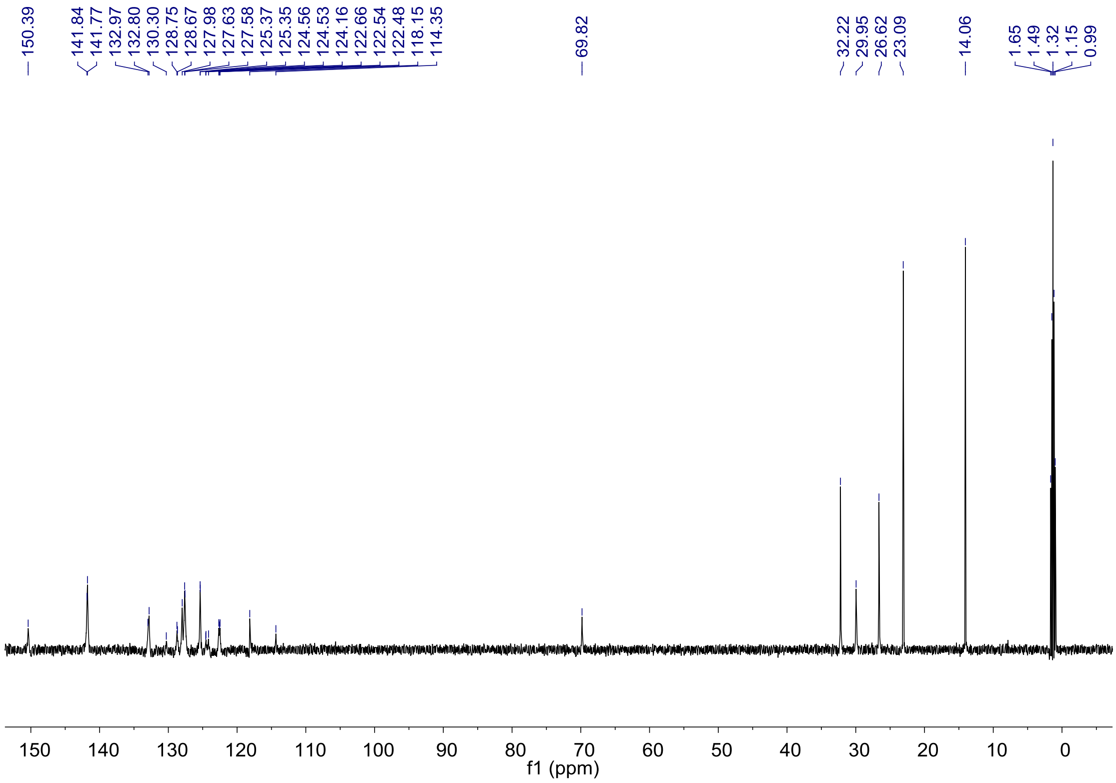

Supplementary Figure 44: ^{13}C DEPT 45° NMR (125 MHz, CD_3CN, 300 K) spectrum of **G2.**

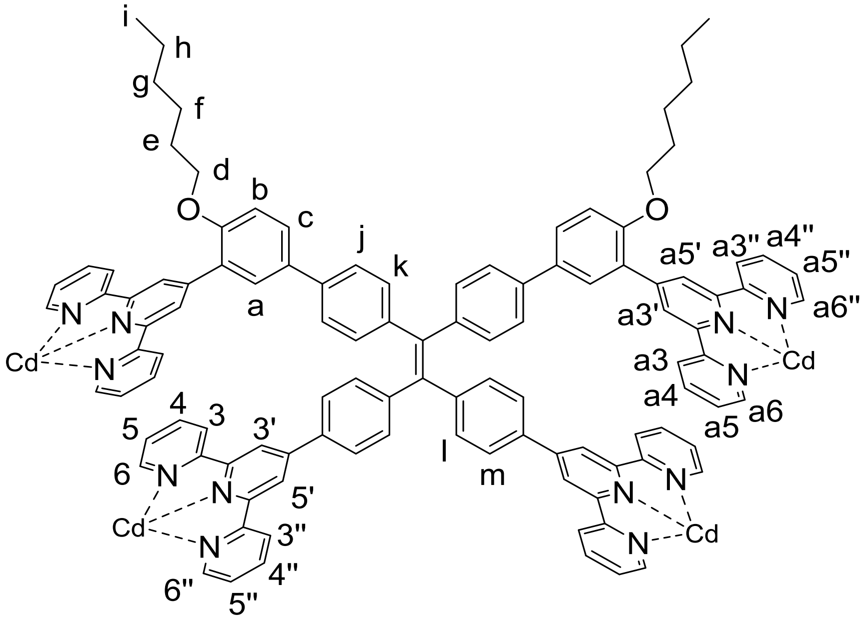

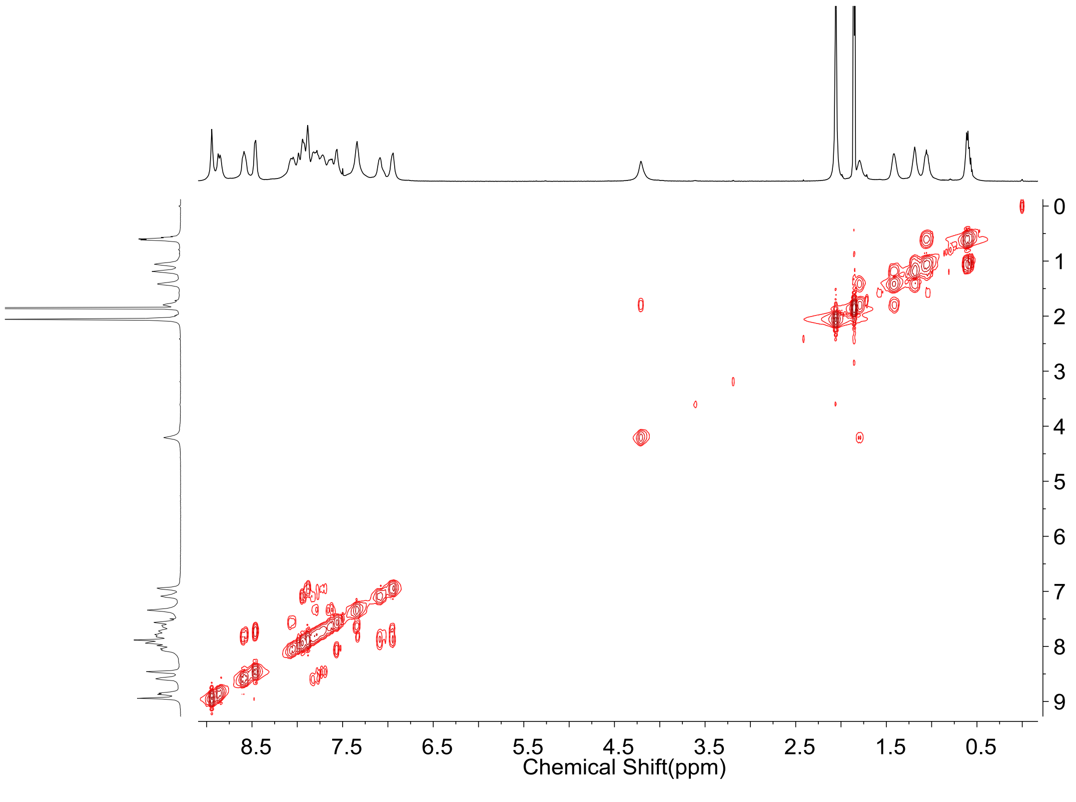

Supplementary Figure 45: 2D COSY NMR (500 MHz, CD_3CN, 300 K) spectrum of **G2**.

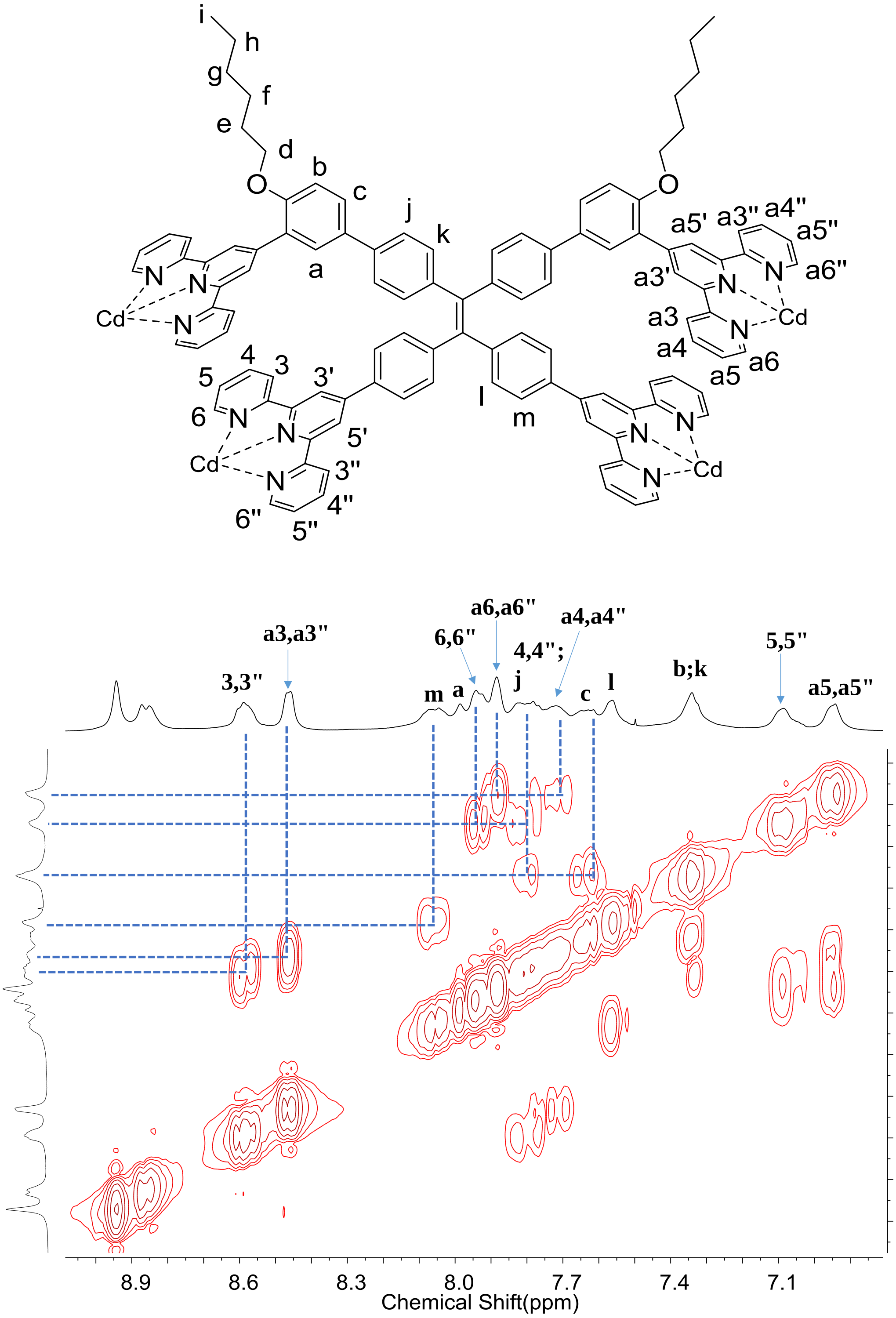

Supplementary Figure 46: 2D COSY NMR (500 MHz, CD_3CN, 300 K) spectrum of **G2** (aromatic region).

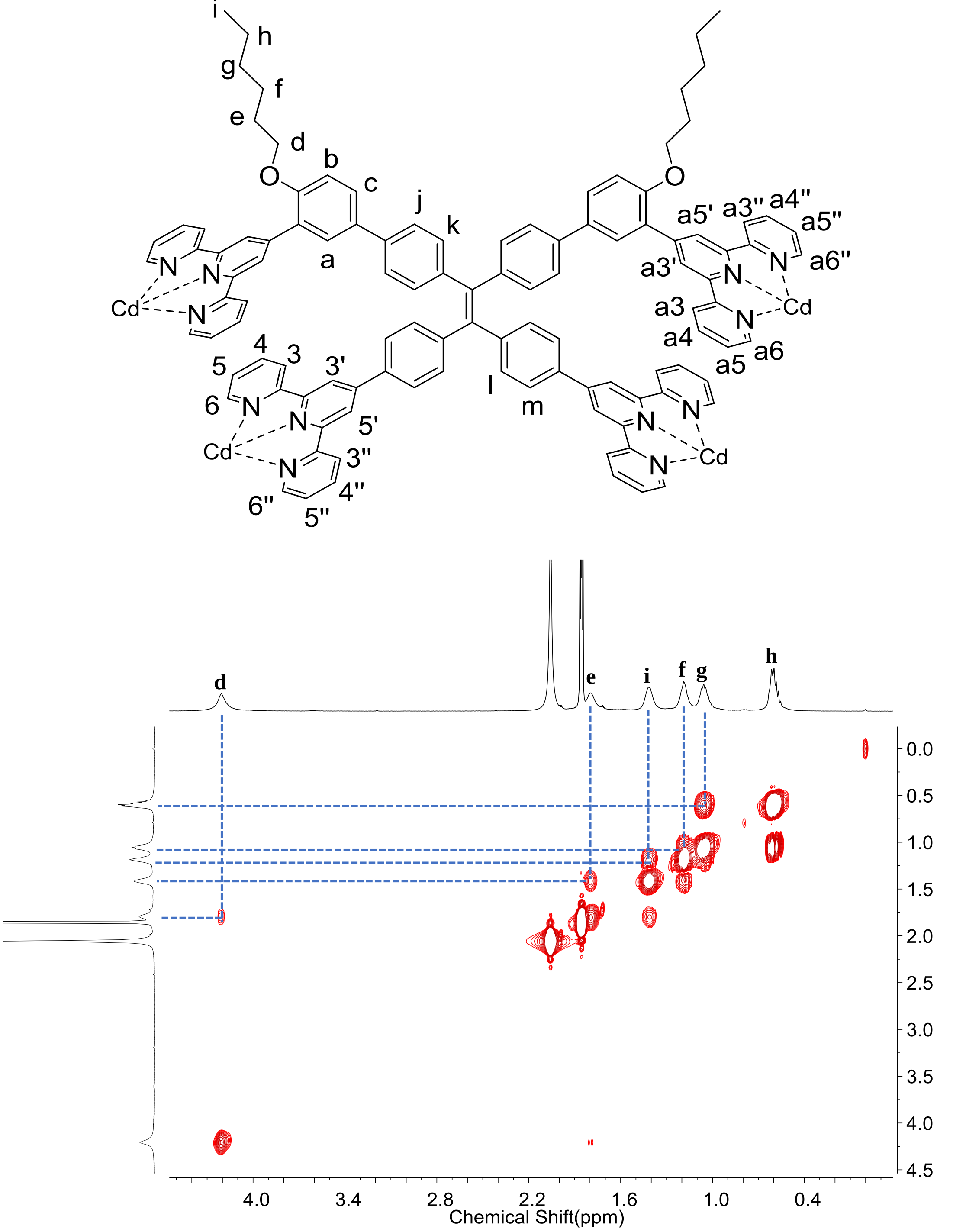

Supplementary Figure 47: 2D COSY NMR (500 MHz, CD_3CN, 300 K) spectrum of **G2** (aliphatic region).

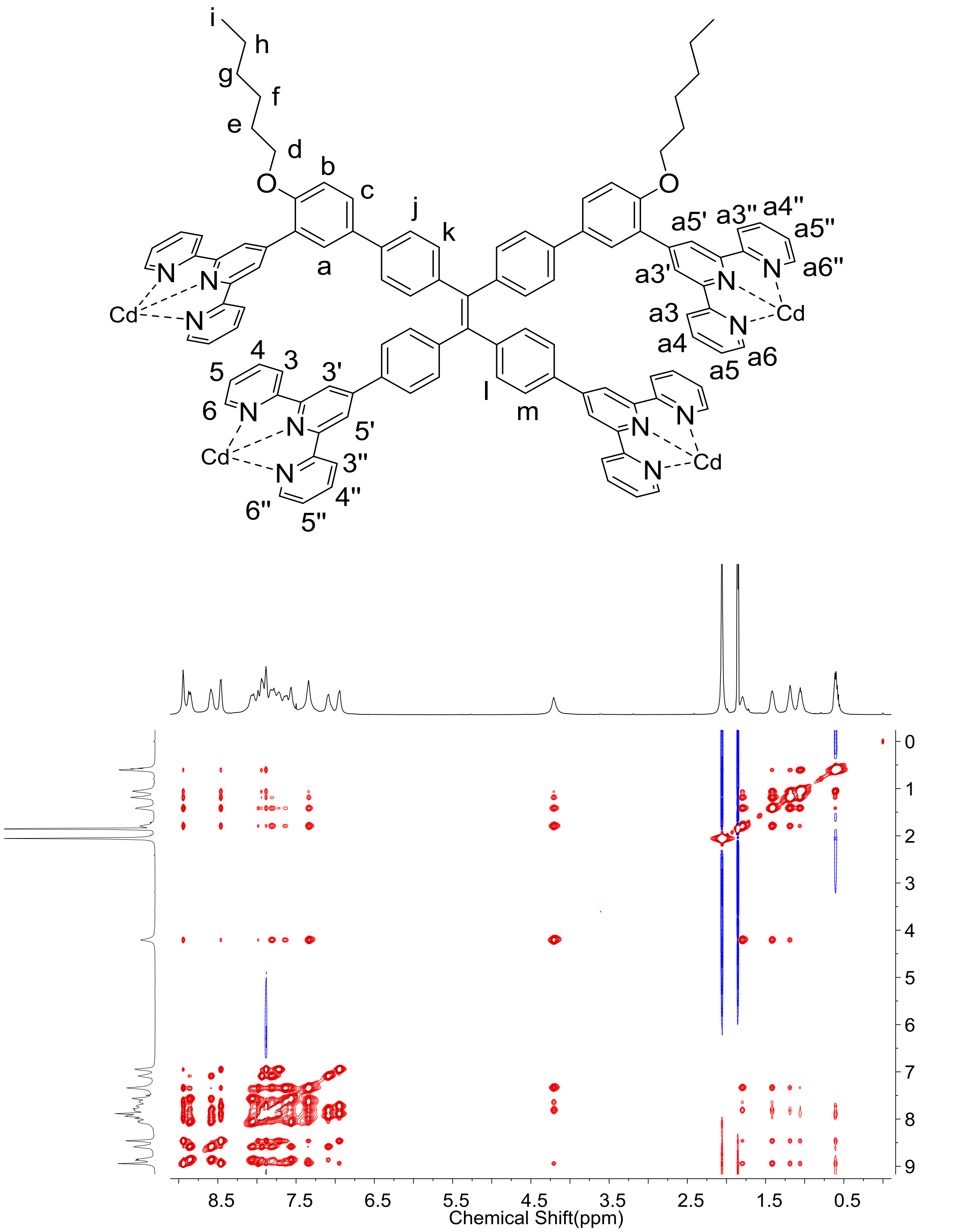

Supplementary Figure 48: 2D NOESY NMR (500 MHz, CD_3CN, 300 K) spectrum of **G2**.

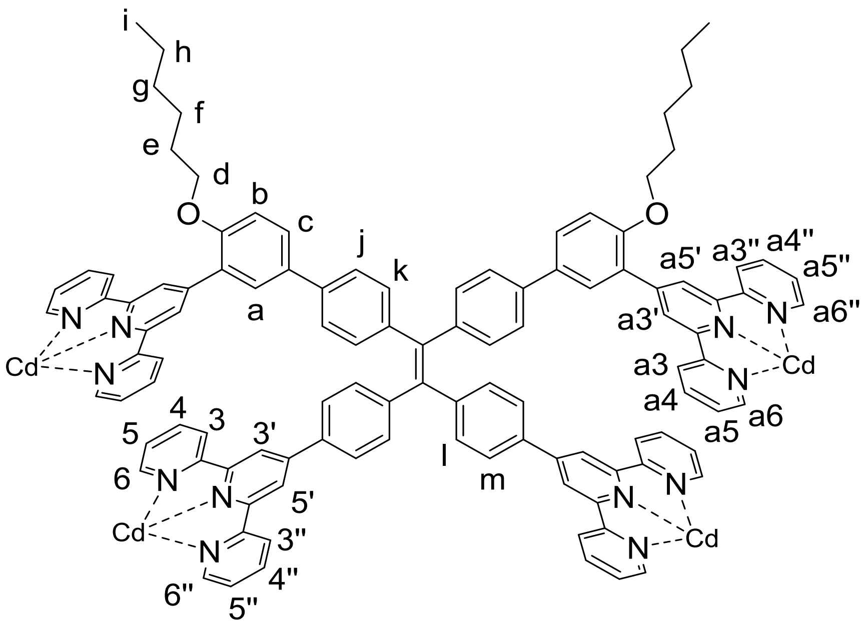

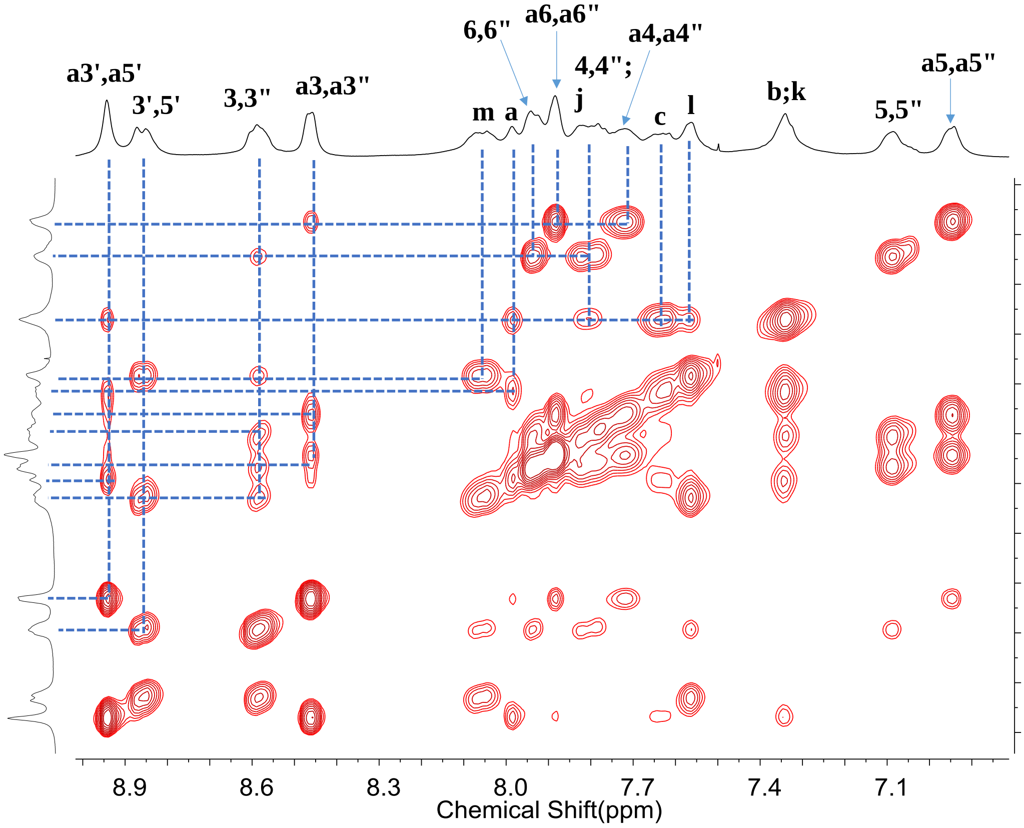

Supplementary Figure 49: 2D NOESY NMR (500 MHz, CD_3CN, 300 K) spectrum of **G2** (aromatic region).

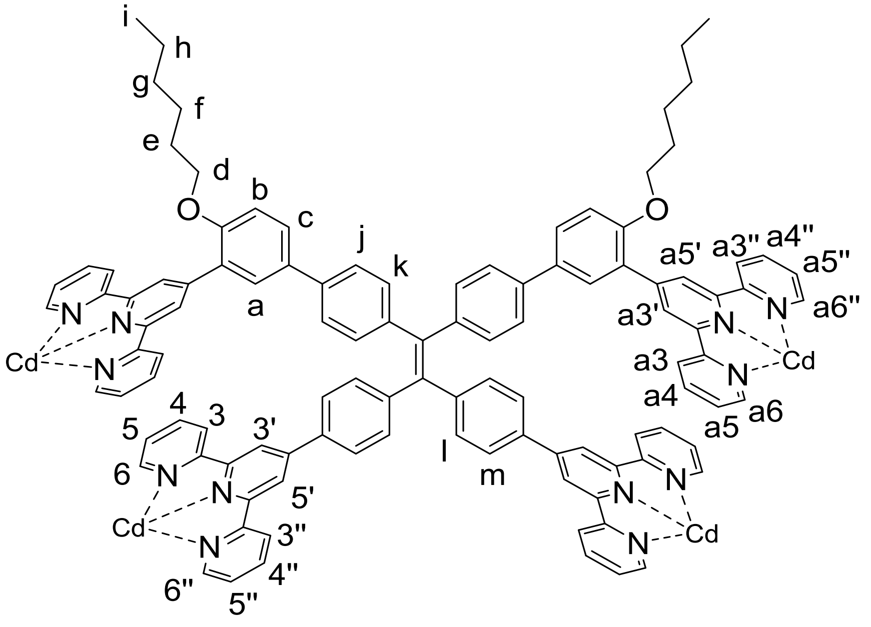

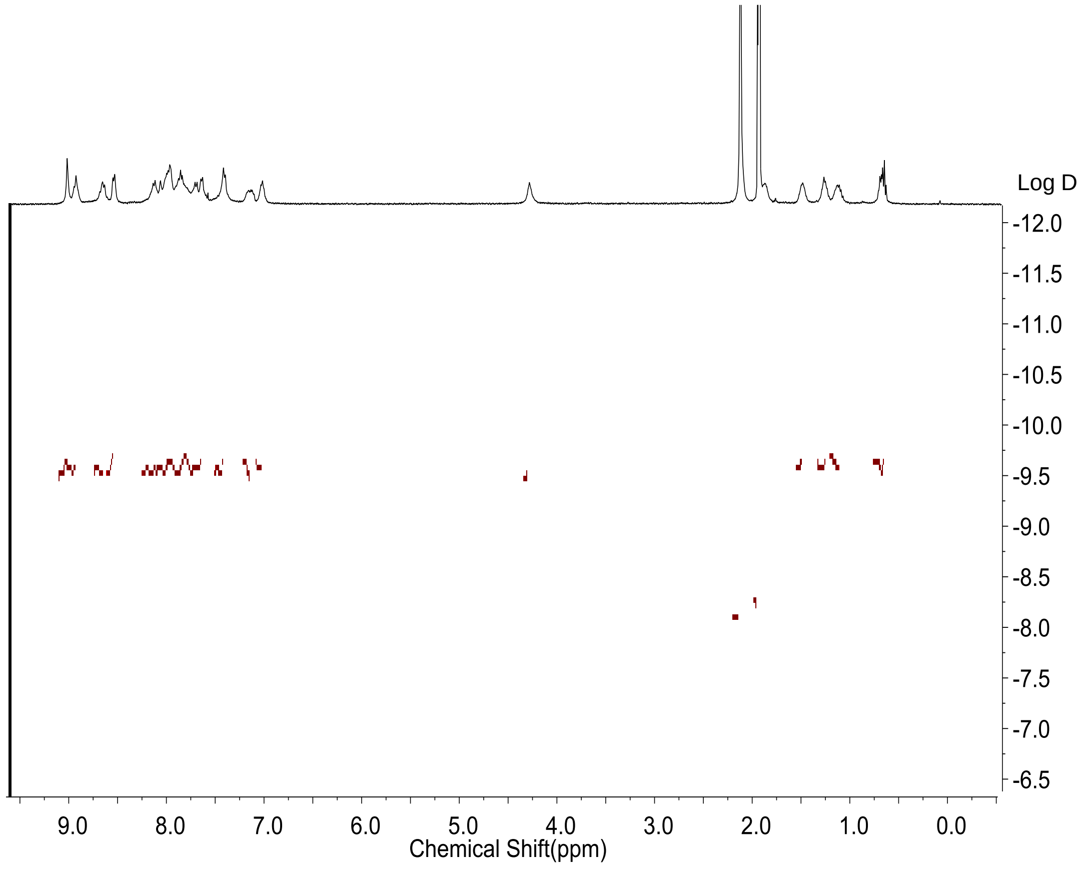

Supplementary Figure 50: 2D DOSY (500 MHz, CD_3CN, 300 K) spectrum of **G2**.

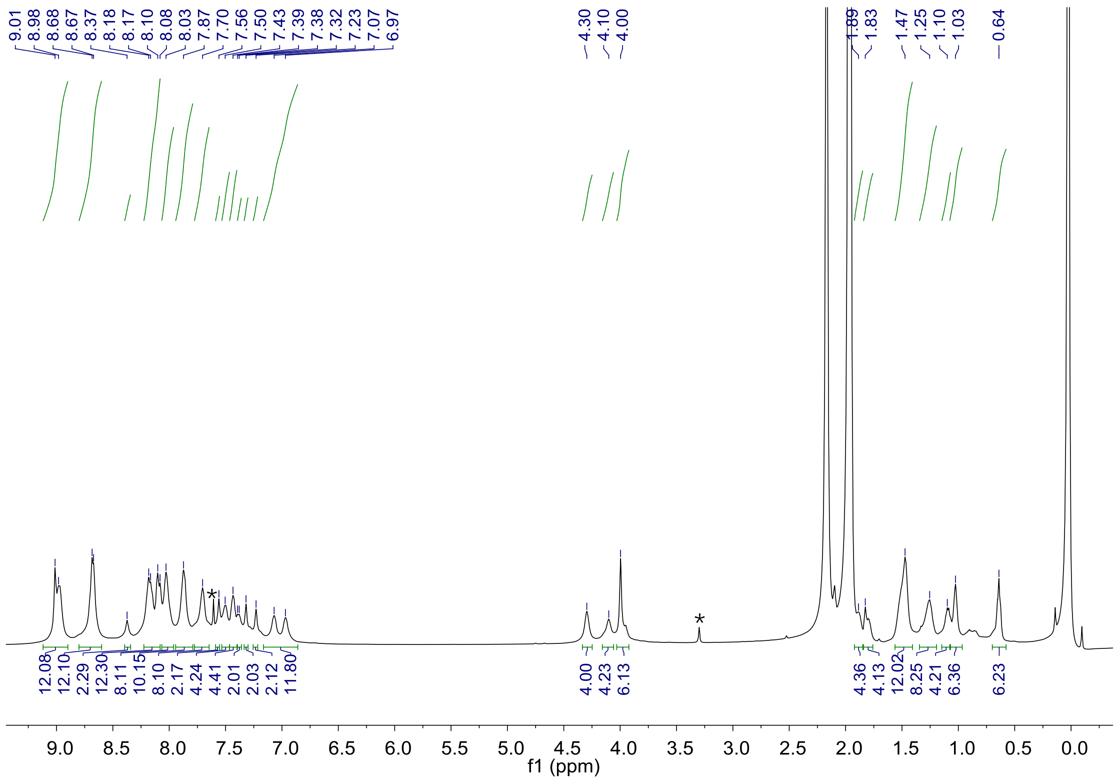

Supplementary Figure 51: ^{1}H NMR (500 MHz, CD_3CN, 300 K) spectrum of **G3.** The * peaks are the chloroform and methanol peaks.

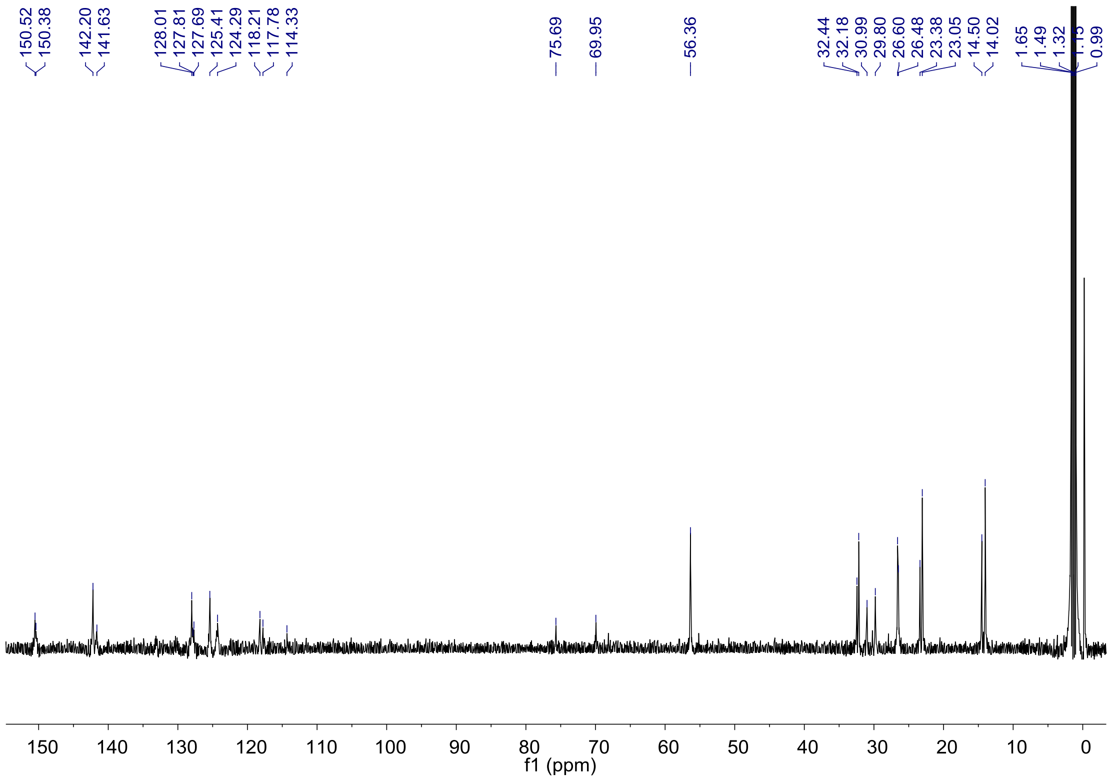

Supplementary Figure 52: ^{13}C DEPT 45° NMR (125 MHz, CD_3CN, 300 K) spectrum of **G3.**

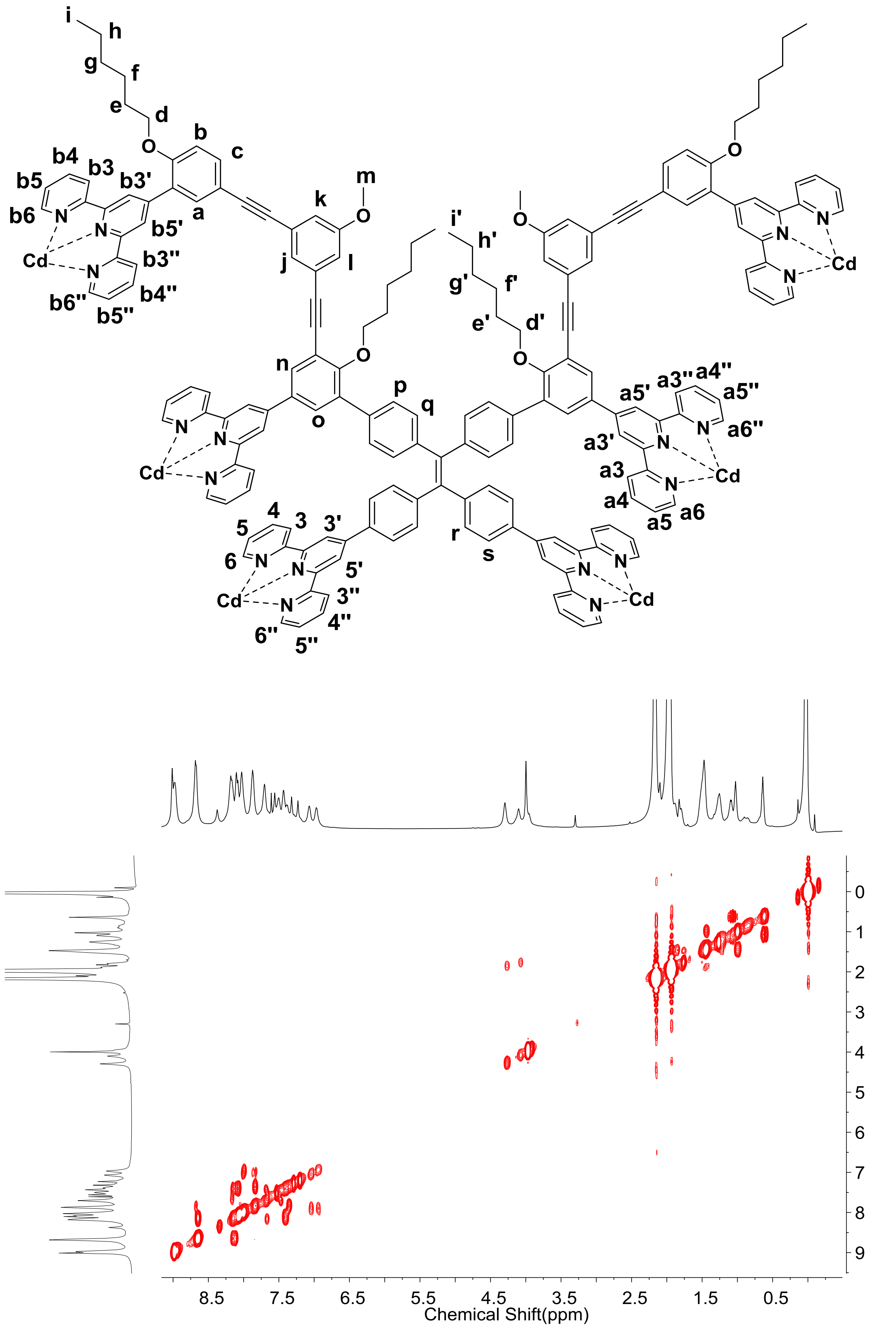

Supplementary Figure 53: 2D COSY NMR (500 MHz, CD_3CN, 300 K) spectrum of **G3.**

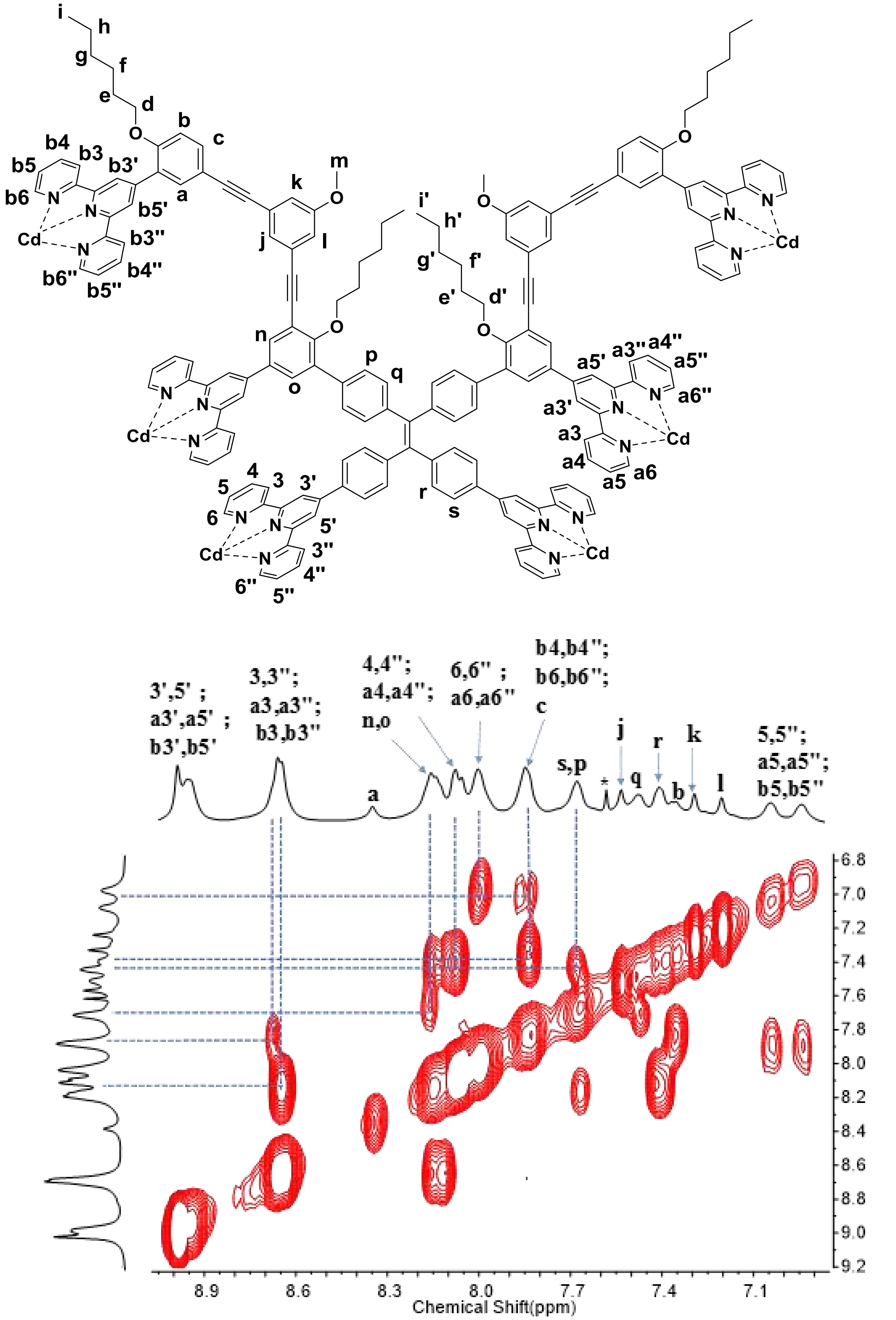

Supplementary Figure 54: 2D COSY NMR (500 MHz, CD_3CN_3, 300 K) spectrum of **G3** (aromatic region).

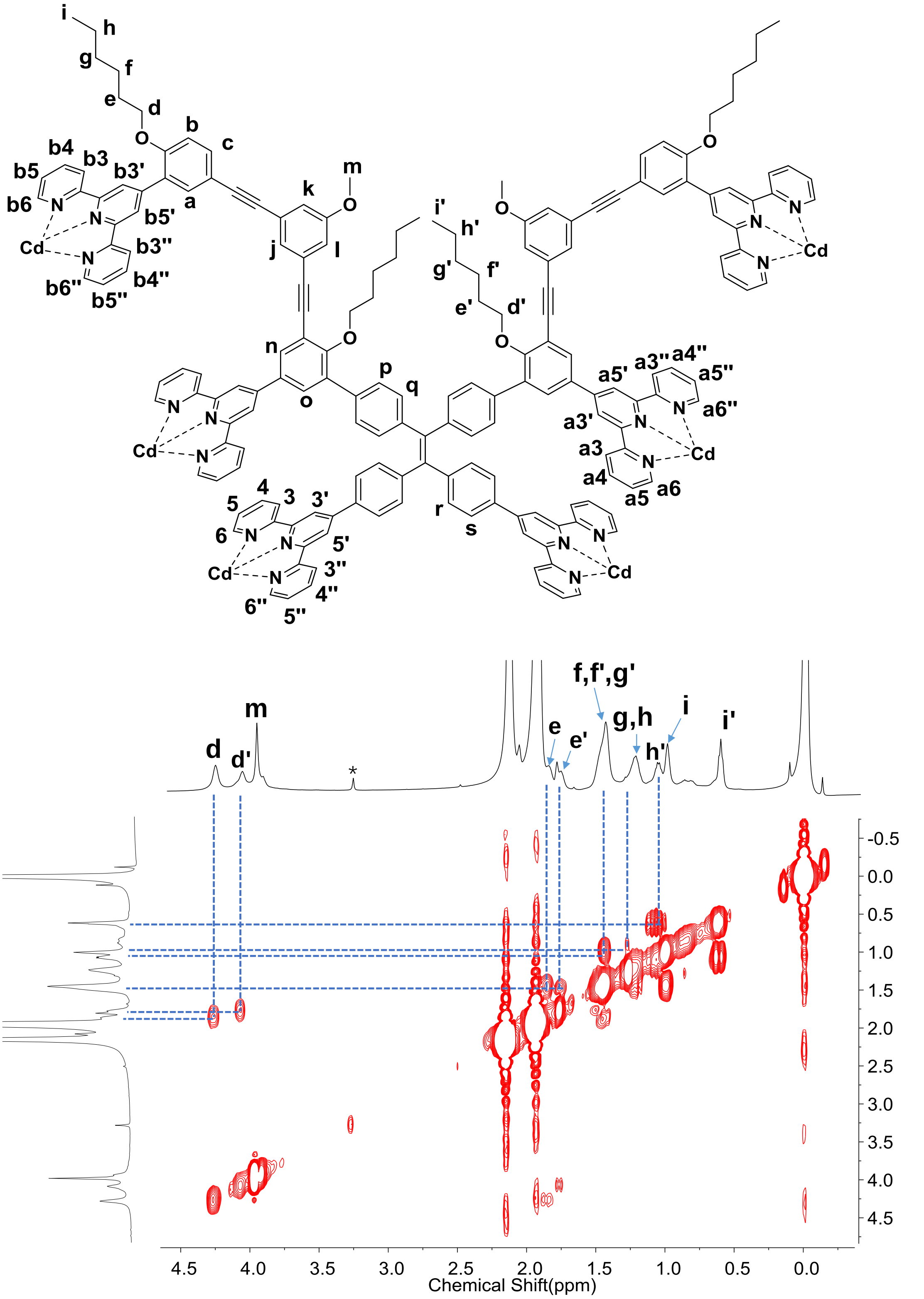

Supplementary Figure 55: 2D COSY NMR (500 MHz, CD_3CN, 300 K) spectrum of **G3** (aliphatic region).

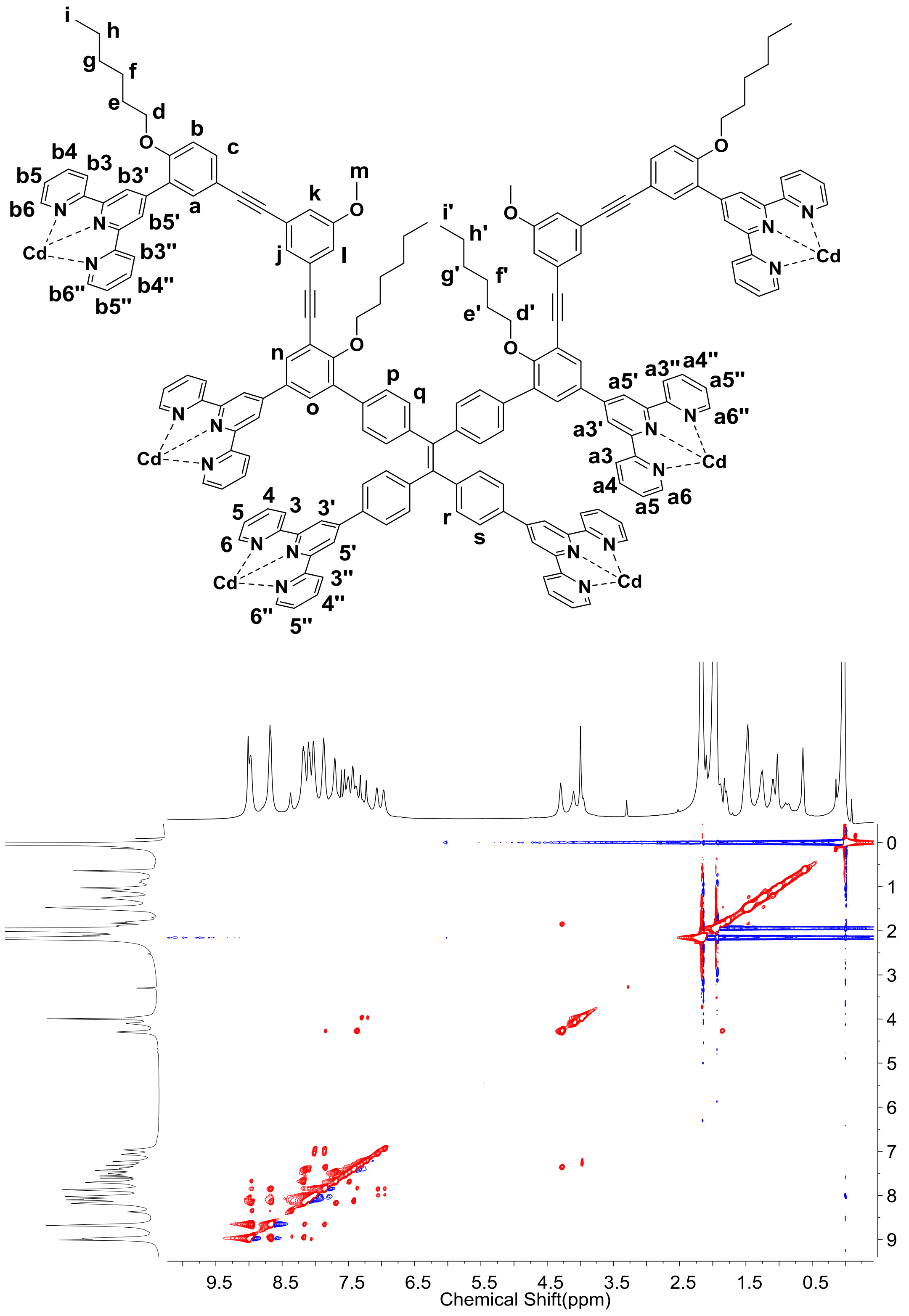

Supplementary Figure 56: 2D NOESY NMR (500 MHz, CD_3CN, 300 K) spectrum of **G3**.

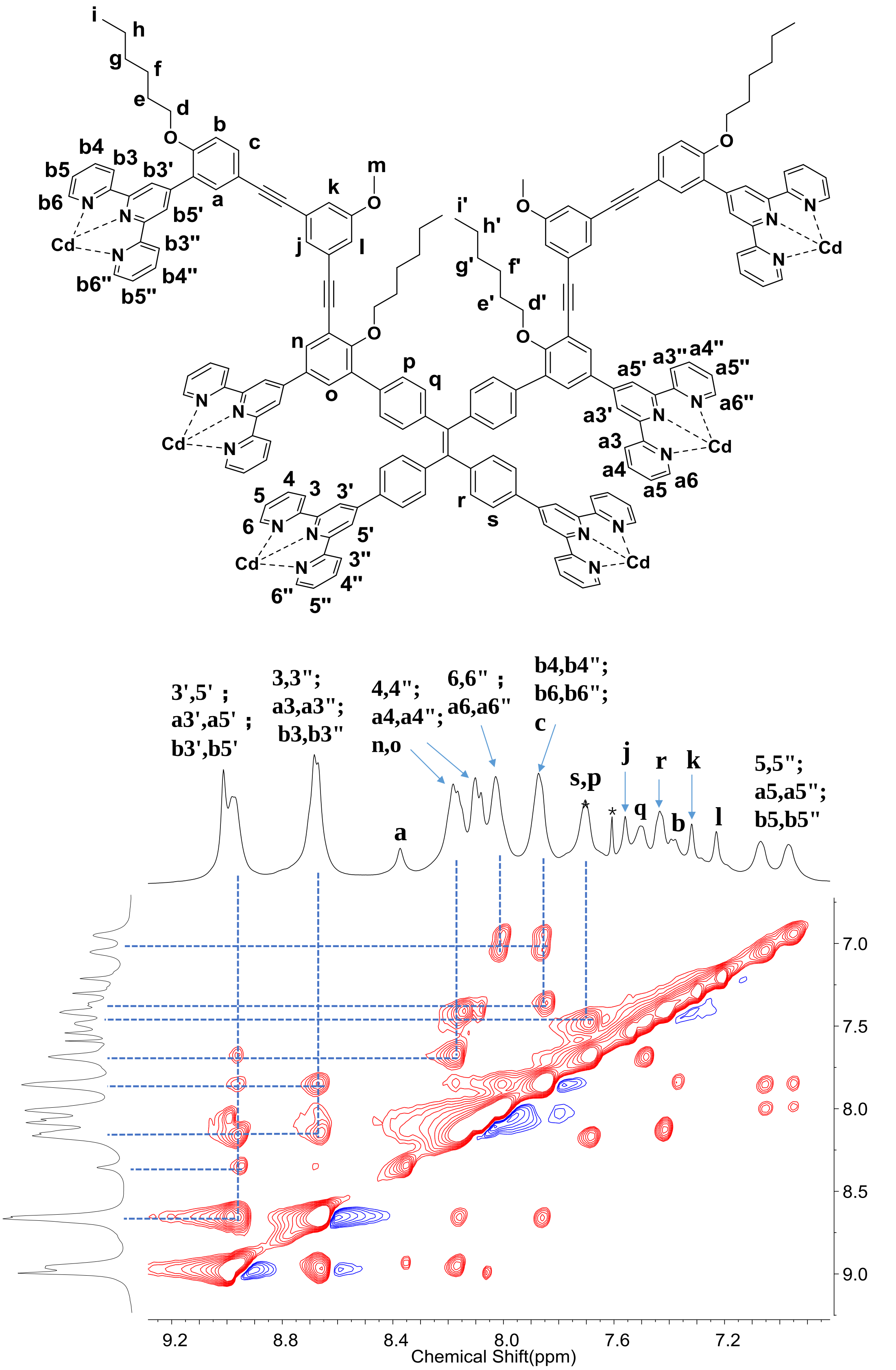

Supplementary Figure 57: 2D NOESY NMR (500 MHz, CD_3CN, 300 K) spectrum of **G3** (aromatic region).

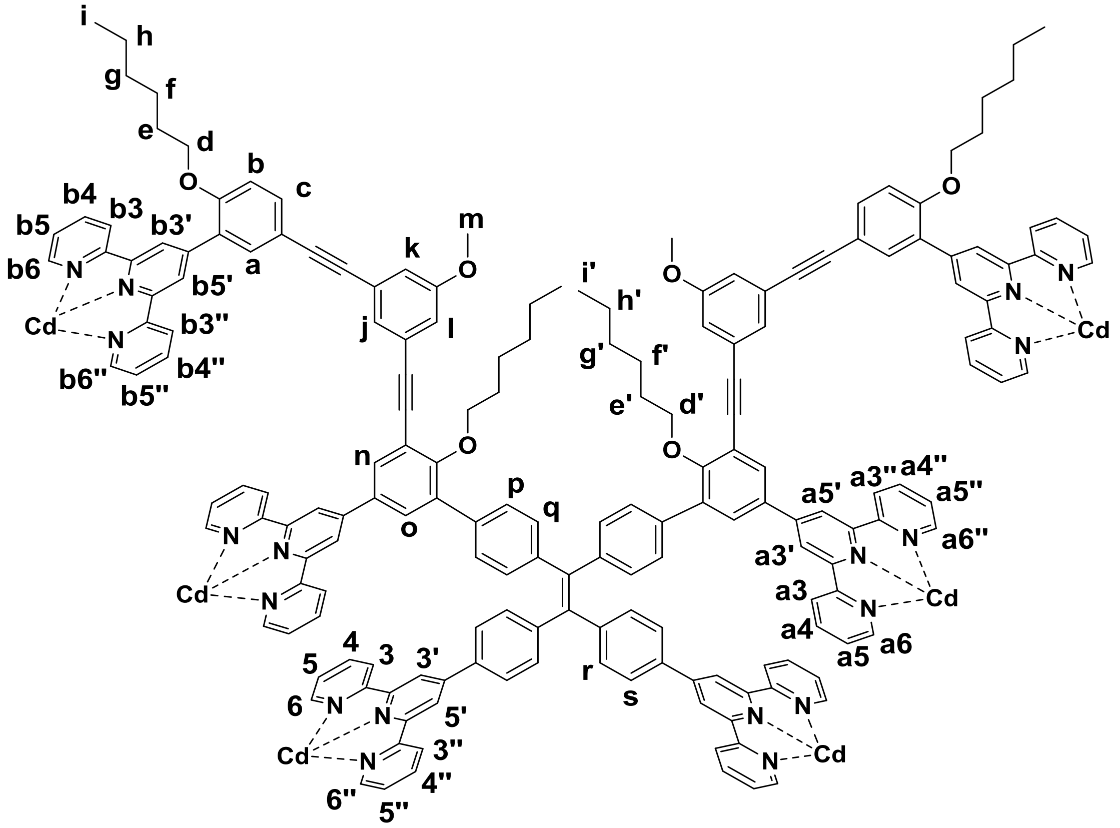

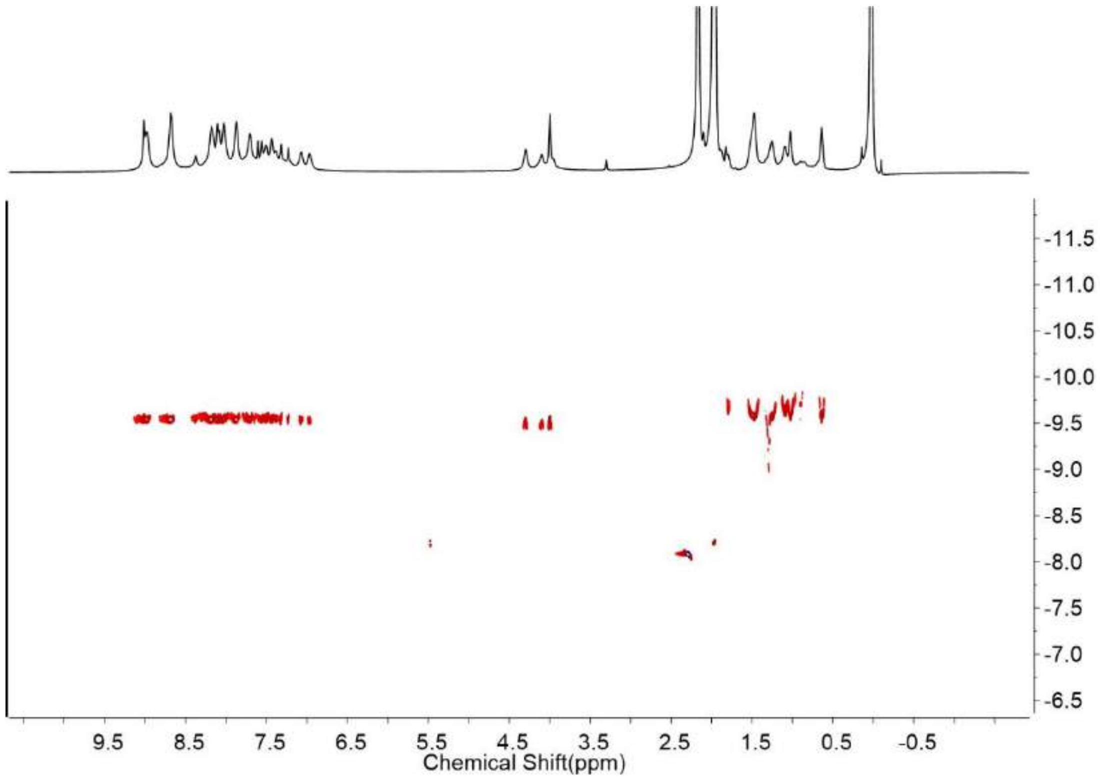

Supplementary Figure 58: 2D DOSY (500 MHz, CD_3CN, 300 K) spectrum of **G3**.

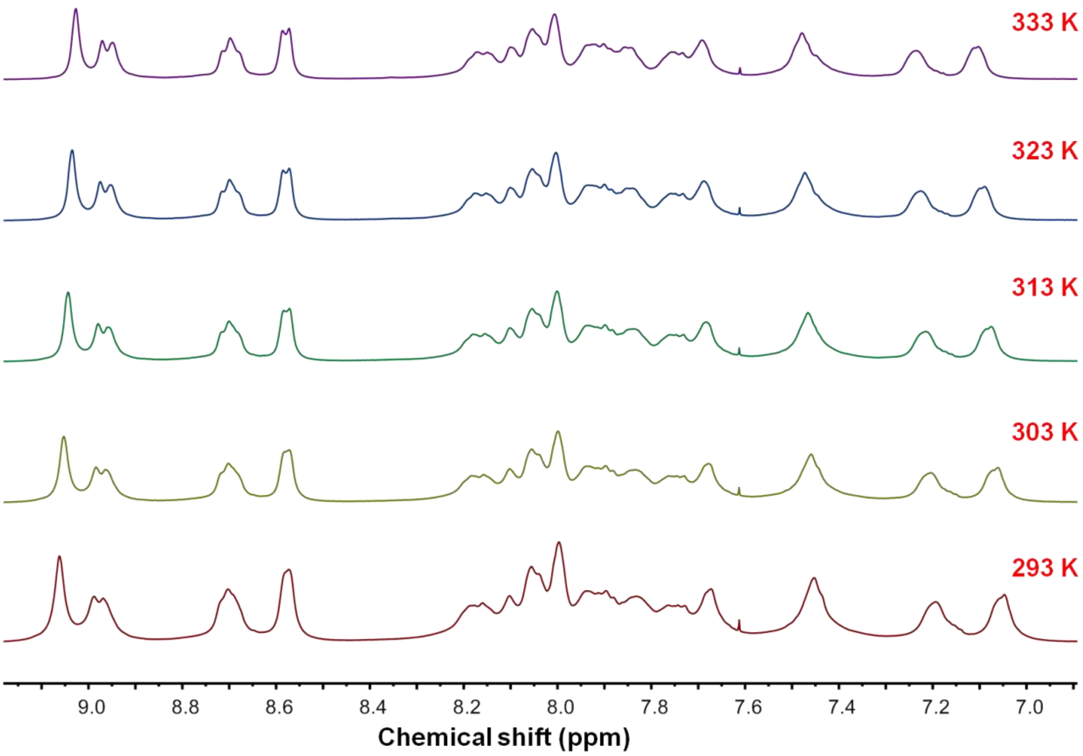

Supplementary Figure 59: Variable temperature ^{1}H NMR spectra (500 MHz) of **G2** in CD_3CN (from 293 K to 333 K).

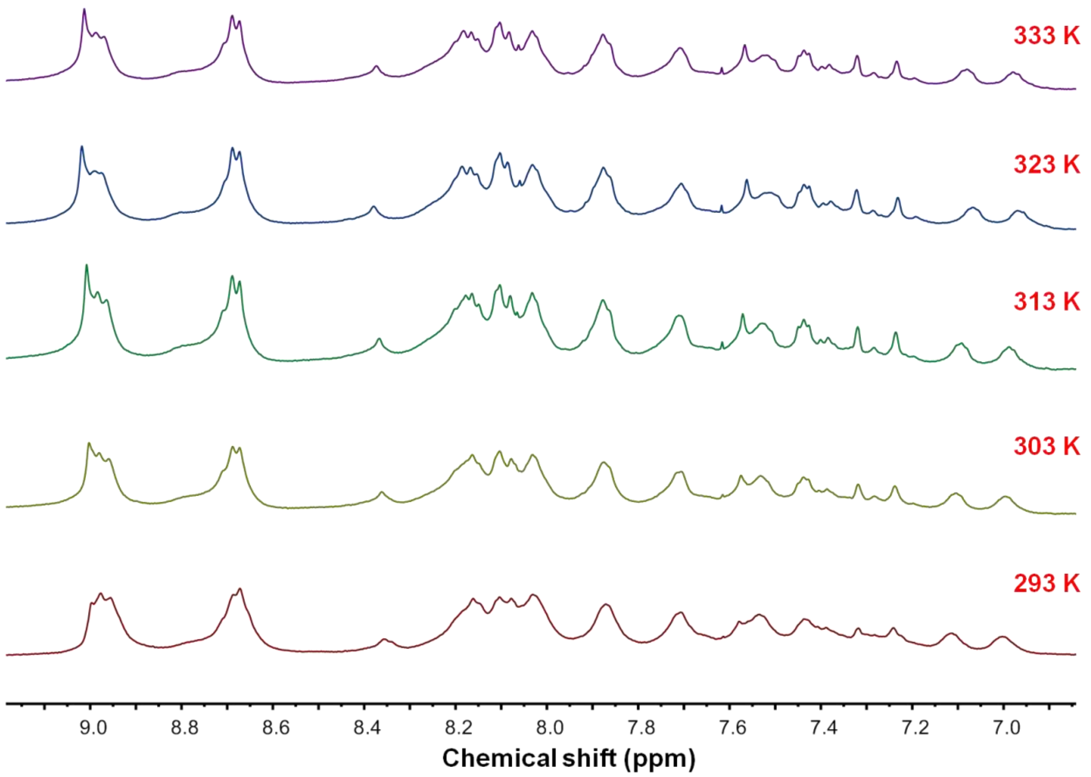

Supplementary Figure 60: Variable temperature ^{1}H NMR spectra (500 MHz) of **G3** in CD_3CN (from 293 K to 333 K).

Absorption spectra of ligands and supramolecular rosettes

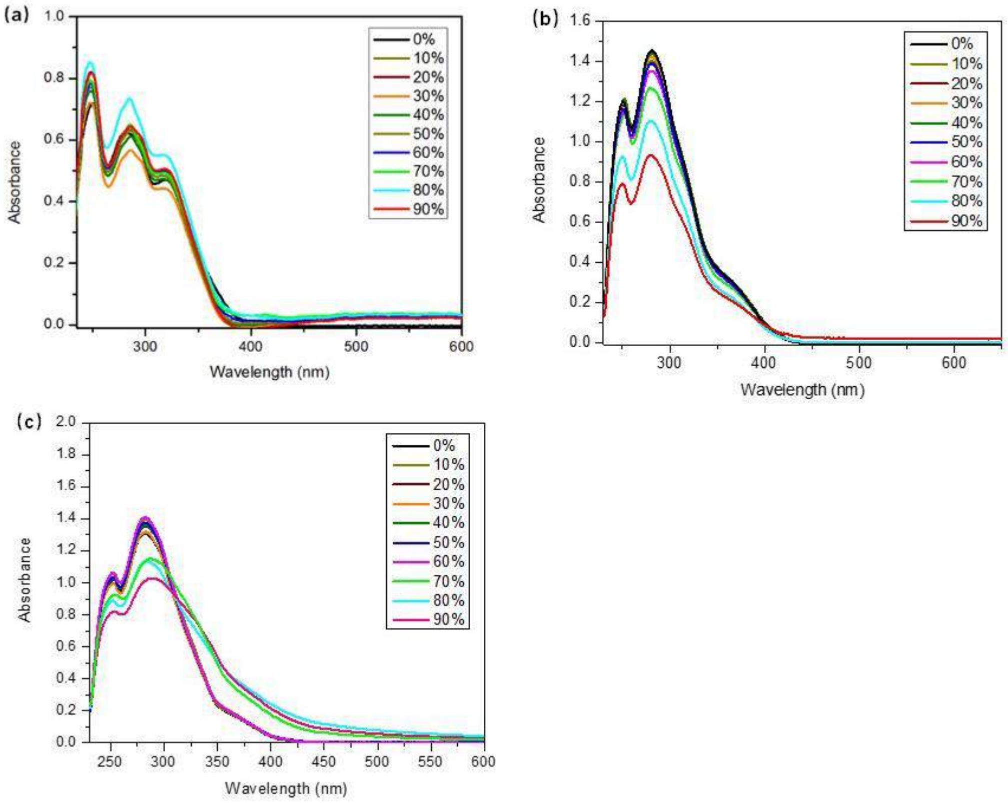

Supplementary Figure 61: Absorption spectra of (a) **L1**, (b) **L2** and (c) **L3** in CH_2Cl_2/methanol mixtures with different methanol contents (c = 10.0 μM).

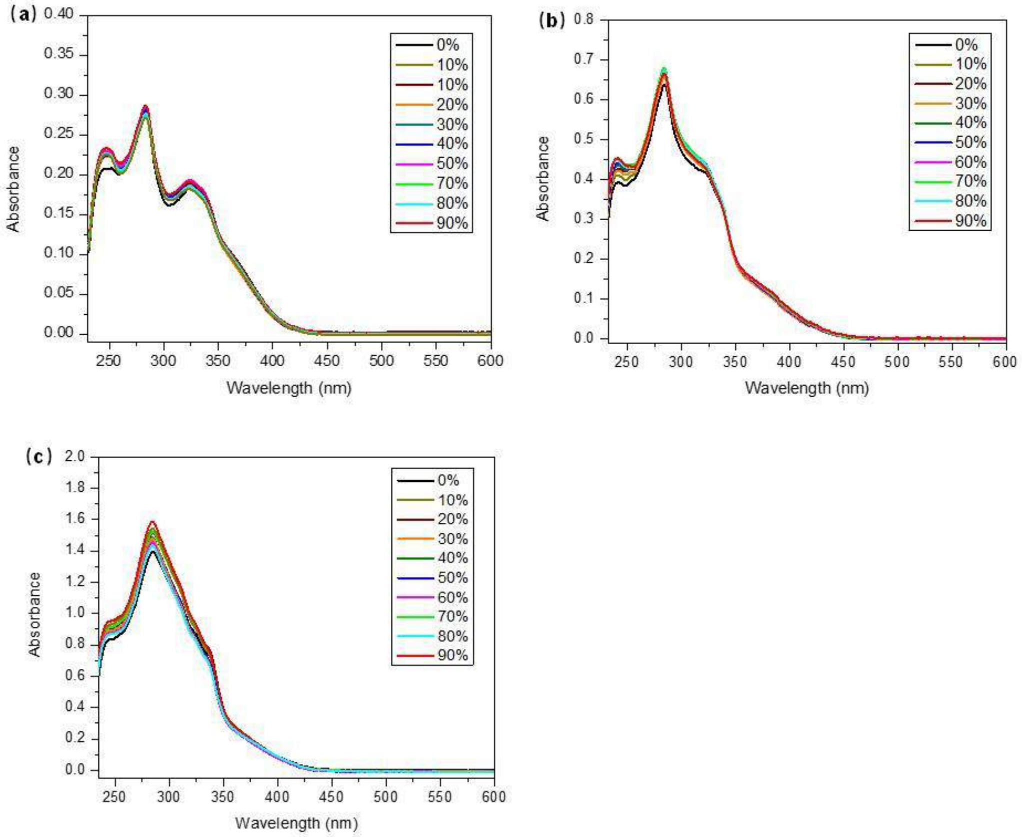

Supplementary Figure 62: Absorption spectra of (a) **G1**, (b) **G2** and (c) **G3** in CH_3CN/methanol mixtures with different methanol contents (c = 1.0 μM).

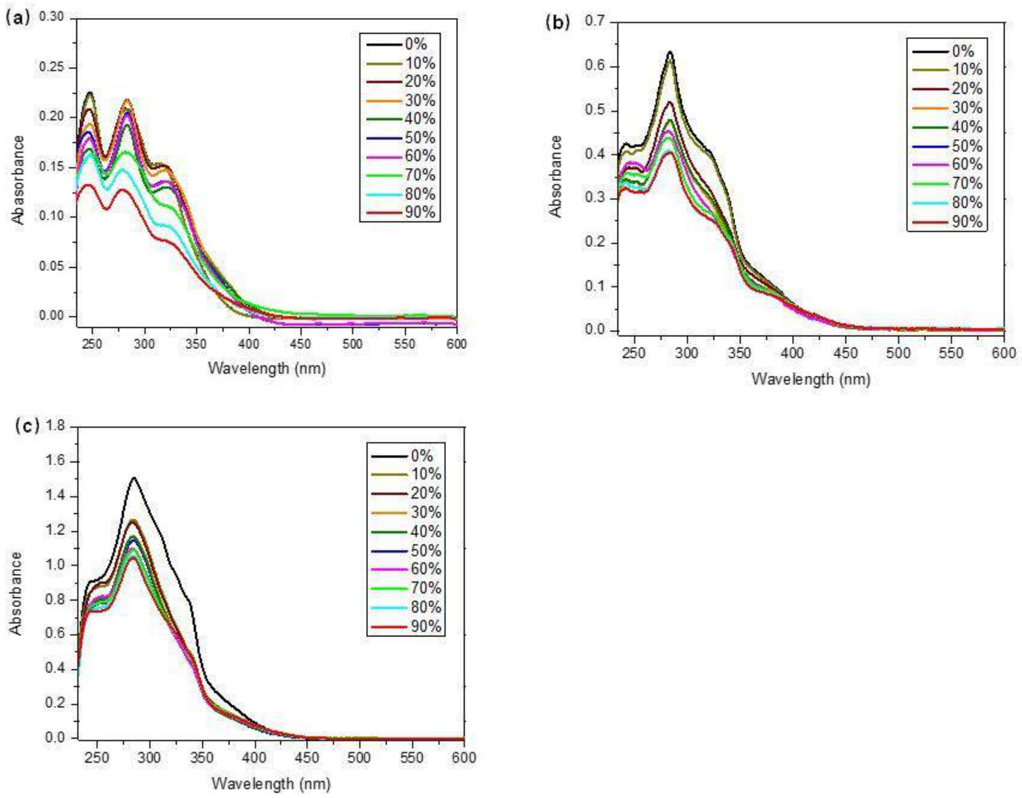

Supplementary Figure 63: Absorption spectra of (a) **G1**, (b) **G2** and (c) **G3** in CH_3CN/ water mixtures with different water contents (c = 1.0 μM).

The determination of association constants in different solvents.

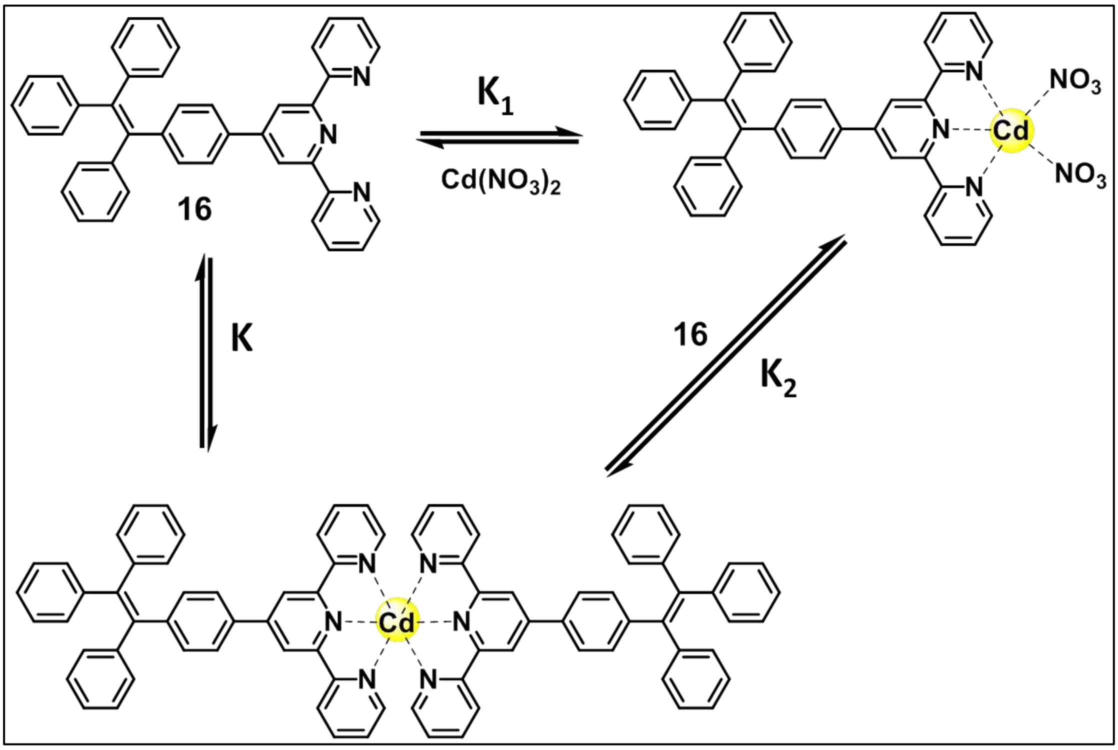

Supplementary Figure 64: Equilibrium for binding Cd^{2+} to compound **16**, where K_1, K_2 are the first and the second binding association constants, respectively, K is the overall association constant ($K=K_1*K_2$).

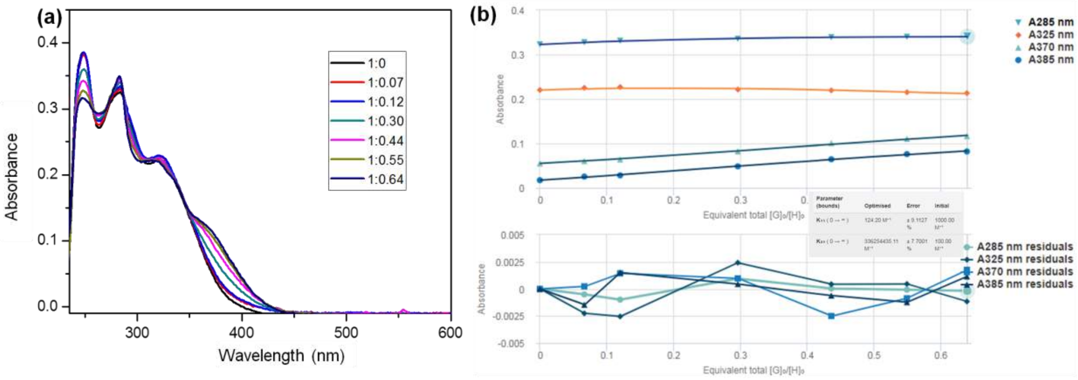

Supplementary Figure 65: (a) UV/Vis spectra for the titration experiment, molar ratio of compound **16**/Cd^{2+} is changed from 1:0 to 1:0.64 in a mixed solvent of $CHCl_3$/MeOH (1/2). The concentration of compound **16** is 10 μM; (b) Binding isotherms (2:1 model) fitted to the absorbance shift *vs.* the equivalents of Cd^{2+} added to determine the association constant (top), the calculated association constant K is 4.17×10^{10} M^{-2}; and the residual plot from the fit (bottom).

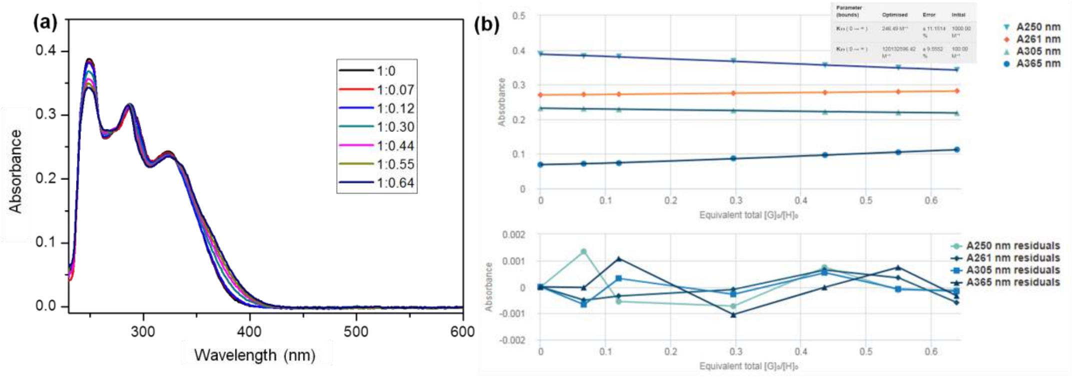

Supplementary Figure 66: (a) UV/Vis spectra for the titration experiment, molar ratio of compound **16**/Cd^{2+} is changed from 1:0 to 1:0.64 in THF. The concentration of compound **16** is 10 μM; (b) Binding isotherms (2:1 model) fitted to the absorbance shift *vs.* the equivalents of Cd^{2+} added to determine the association constant (top), the calculated association constant K is 2.96×10^{10} M^{-2}; and the residual plot from the fit (bottom).

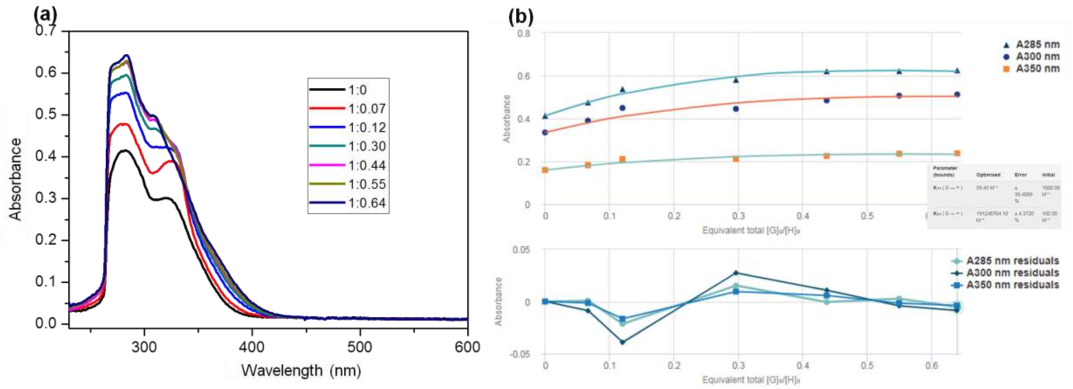

Supplementary Figure 67: (a) UV/Vis spectra for the titration experiment, molar ratio of compound **16**/Cd^{2+} is changed from 1:0 to 1:0.64 in DMF. The concentration of compound **16** is 10 μM; (b) Binding isotherms (2:1 model) fitted to the absorbance shift *vs*. the equivalents of Cd^{2+} added to determine the association constant (top), the calculated association constant K is 1.13 $\times 10^{10}$ M^{-2}; and the residual plot from the fit (bottom).

Fluorescence emission spectra of ligands and supramolecular rosettes

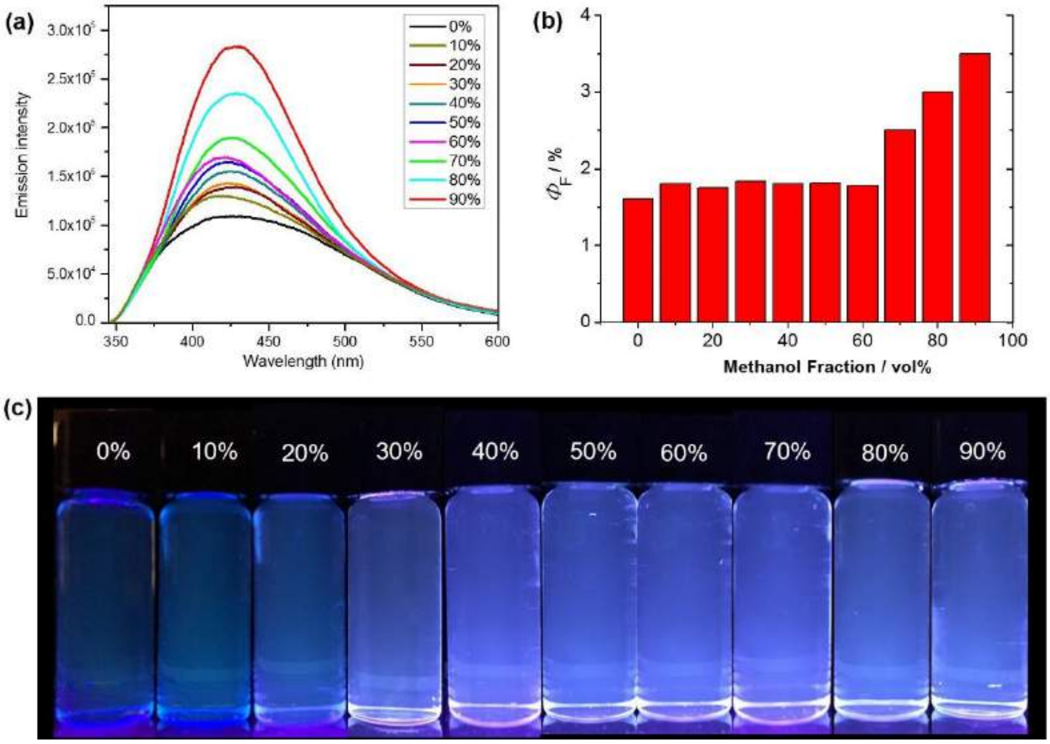

Supplementary Figure 68: (a) Fluorescence spectra, (b) quantum yields of **L1** in CH_2Cl_2/methanol mixtures with different methanol contents (λ_{ex} = 320 nm, c = 1.0 μM), and (c) the photographs of **L1** in CH_2Cl_2/methanol mixtures with different fractions of methanol on excitation at 365 nm at 298 K (c = 10 μM).

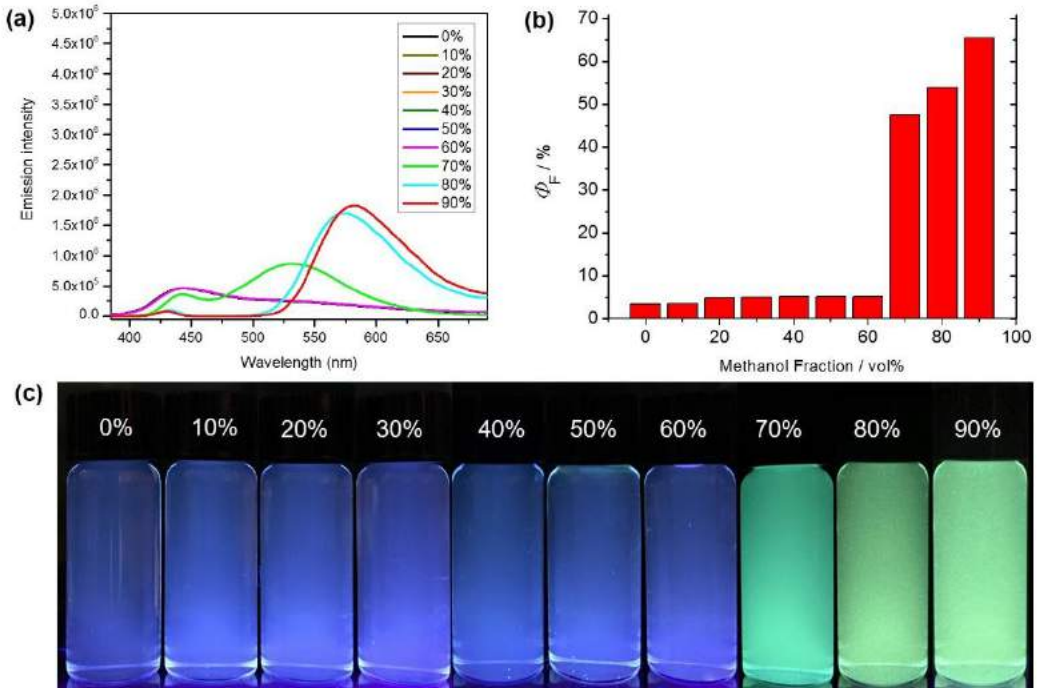

Supplementary Figure 69: (a) Fluorescence spectra, (b) quantum yields of **L2** in CH_2Cl_2/methanol mixtures with different methanol contents (λ_{ex} = 320 nm, c = 1.0 μM), and (c) the photographs of **L2** in CH_2Cl_2/methanol mixtures with different fractions of methanol on excitation at 365 nm at 298 K (c = 10 μM).

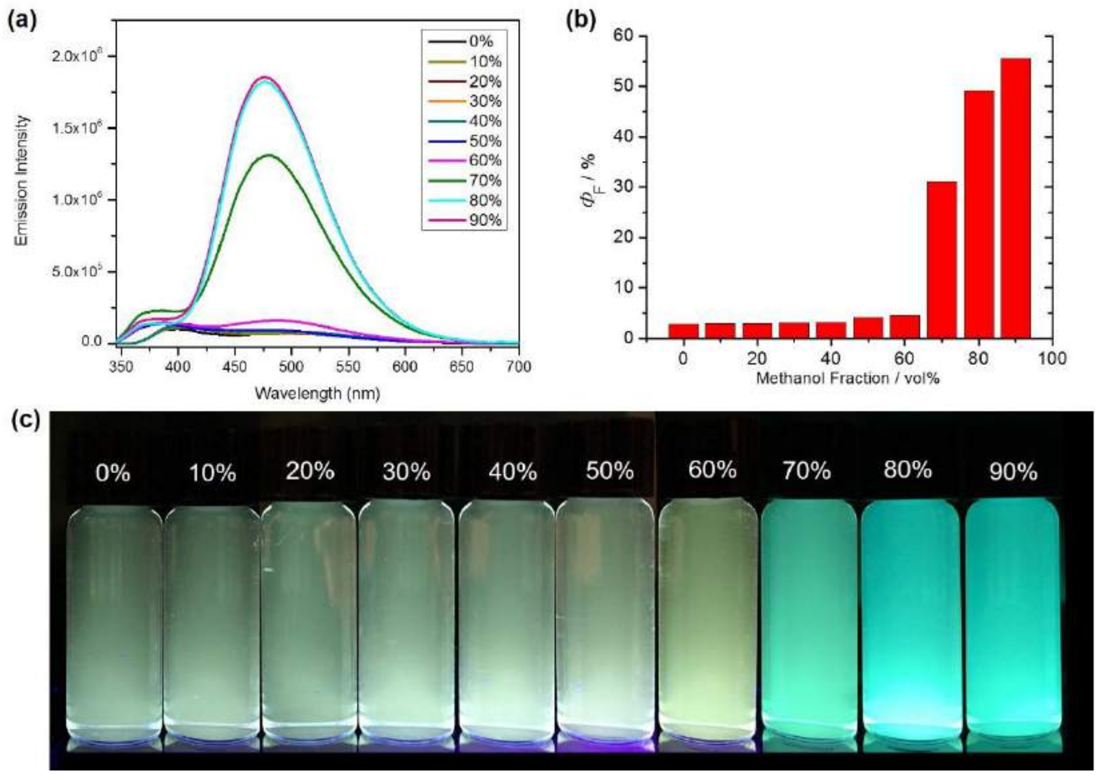

Supplementary Figure 70: (a) Fluorescence spectra, (b) quantum yields of **L3** in CH_2Cl_2/methanol mixtures with different methanol contents (λ_{ex} = 320 nm, *c* = 1.0 μM), and (c) the photographs of **L3** in CH_2Cl_2/methanol mixtures with different fractions of methanol on excitation at 365 nm at 298 K (*c* = 10 μM).

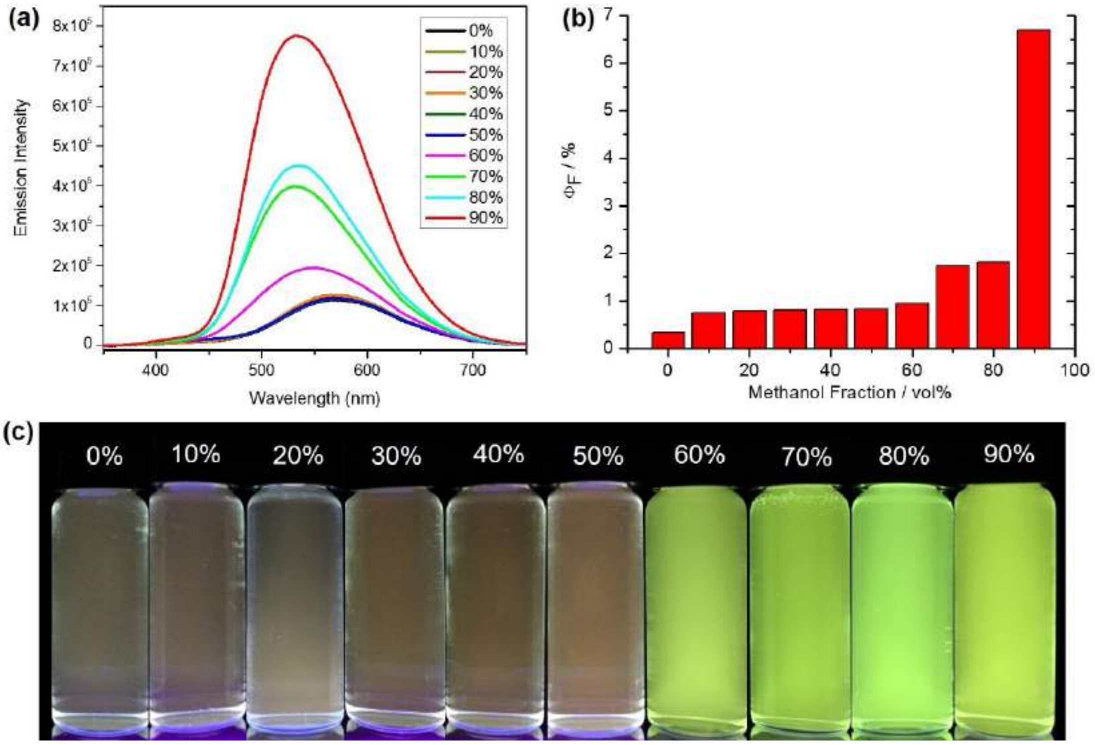

Supplementary Figure 71: (a) Fluorescence spectra, (b) quantum yields of **G1** in CH_3CN/methanol mixtures with different methanol contents (λ_{ex} = 320 nm, c = 1.0 μM), and (c) the photographs of **G1** in CH_3CN/methanol mixtures with different fractions of methanol on excitation at 365 nm at 298 K (c = 1.0 μM).

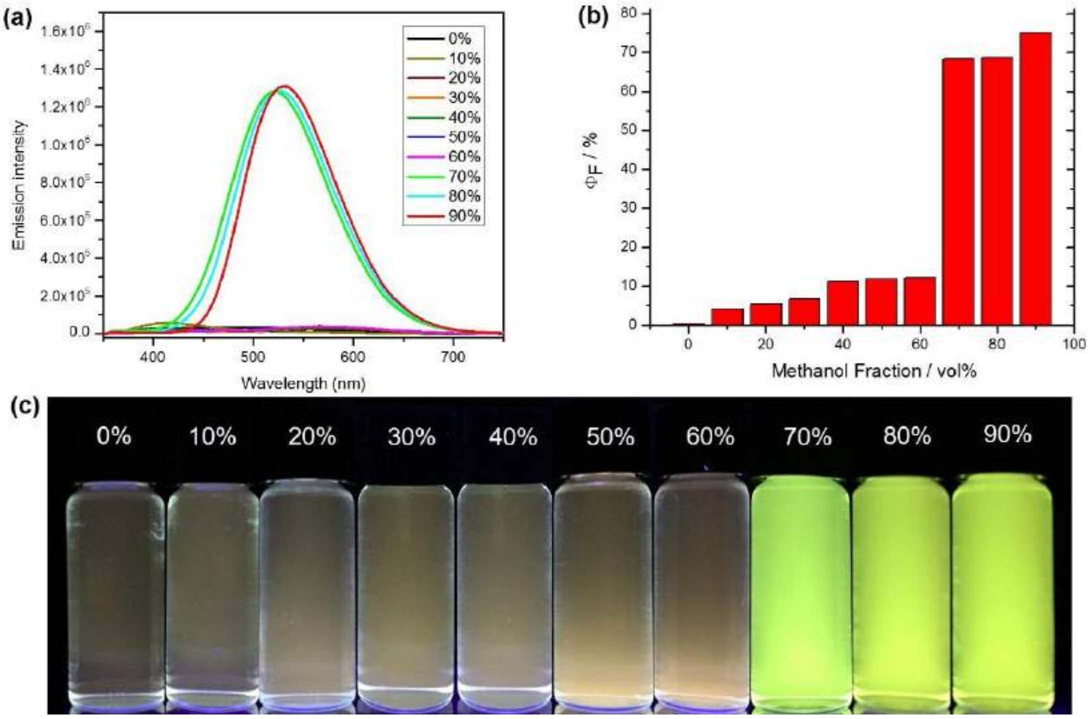

Supplementary Figure 72: (a) Fluorescence spectra, (b) quantum yields of **G1** in CH_3CN/H_2O mixtures with different water contents (λ_{ex} = 320 nm, c = 1.0 μM), and (c) the photographs of **G1** in CH_3CN/H_2O mixtures with different fractions of water on excitation at 365 nm at 298 K (c = 1.0 μM).

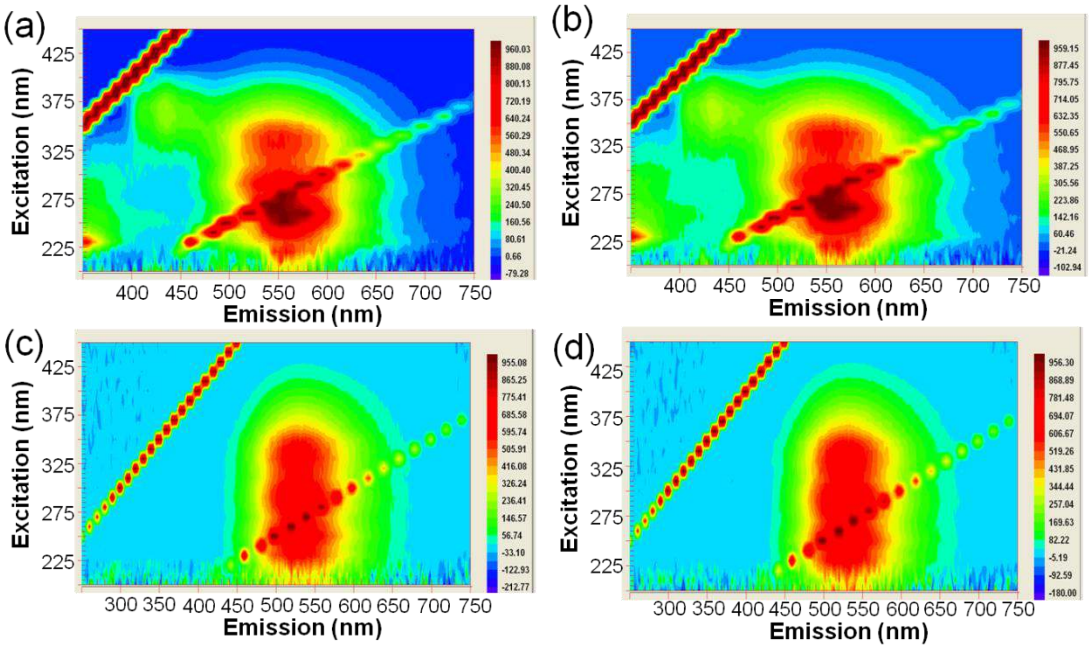

Supplementary Figure 73: Multi-wavelength (2D) fluorescence spectrum (excitation step width: 10 nm) of **G1** (c = 1.0 μM) in CH_3CN/methanol mixtures with different methanol contents (a) 0 %, (b) 30 %, (c) 60 %, (d) 90 %.

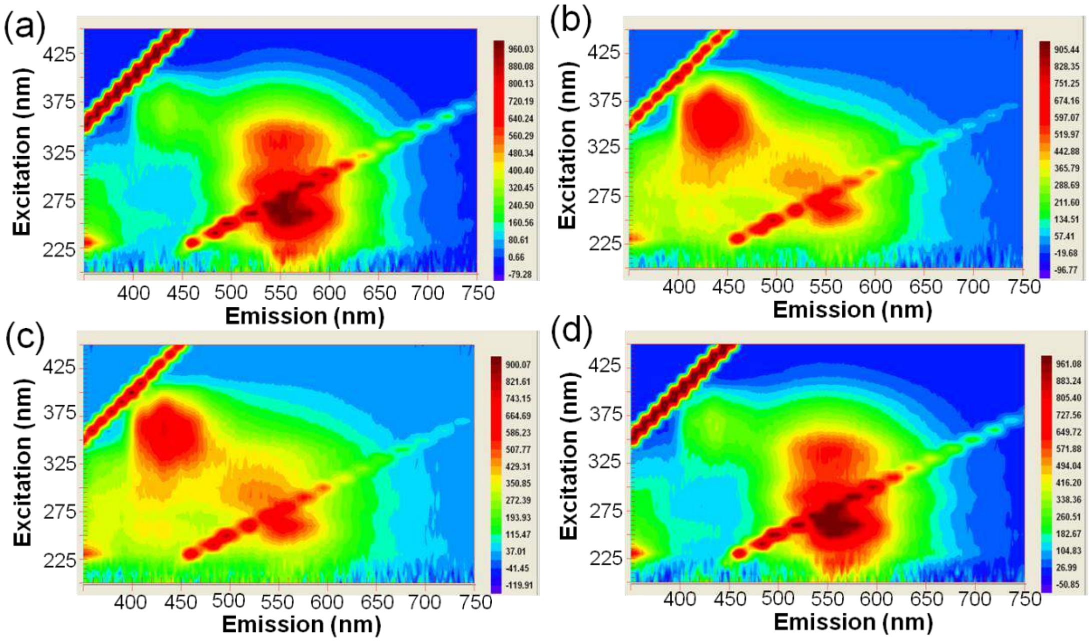

Supplementary Figure 74: Multi-wavelength (2D) fluorescence spectrum (excitation step width: 10 nm) of **G1** (c = 1.0 μM) in CH_3CN/H_2O mixtures with different water contents (a) 0 %, (b) 30 %, (c) 60 %, (d) 90 %.

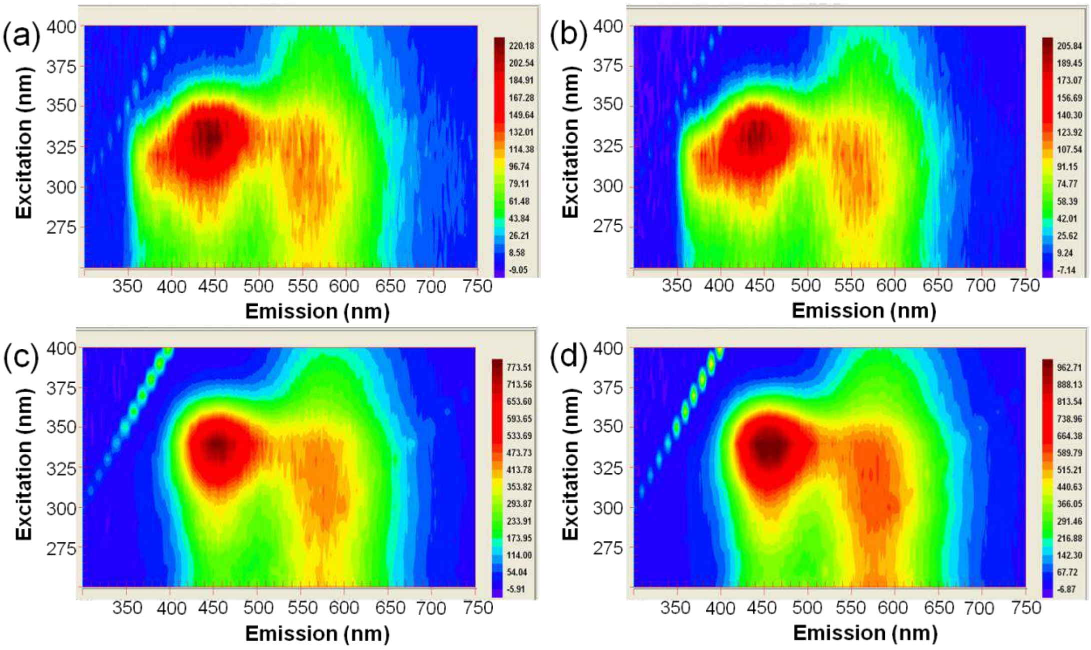

Supplementary Figure 75: Multi-wavelength (2D) fluorescence spectrum (excitation step width: 10 nm) of **G2** (c = 1.0 μM) in CH_3CN/methanol mixtures with different methanol contents (a) 0 %, (b) 30 %, (c) 60 %, (d) 90 %.

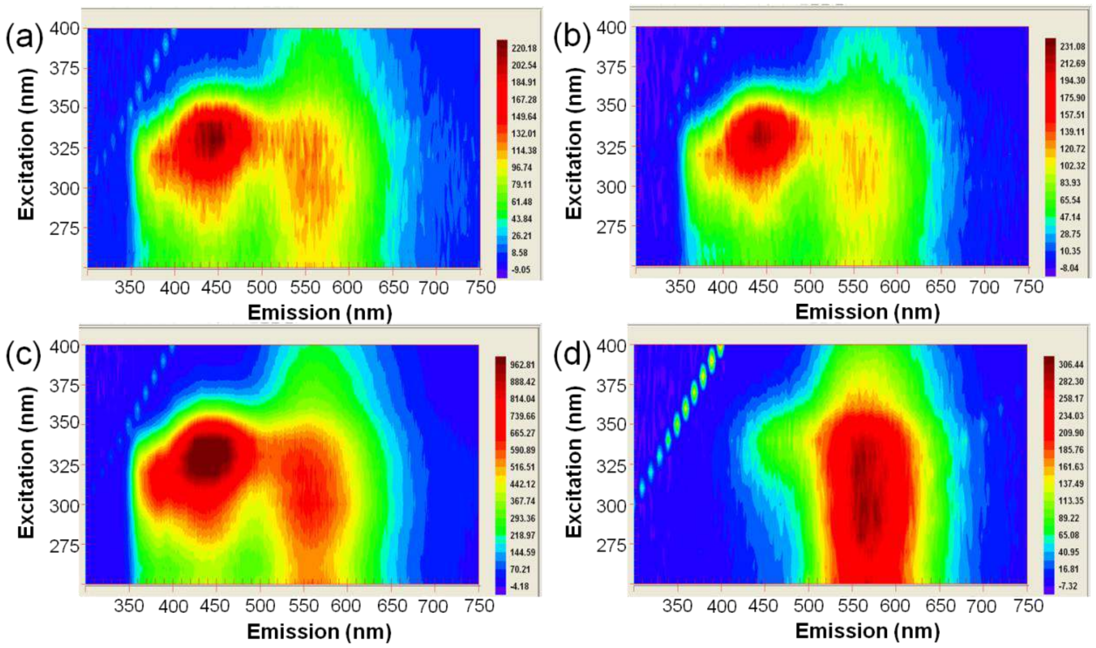

Supplementary Figure 76: Multi-wavelength (2D) fluorescence spectrum (excitation step width: 10 nm) of **G2** (c = 1.0 μM) in CH_3CN/H_2O mixtures with different water contents (a) 0 %, (b) 30 %, (c) 60 %, (d) 90 %.

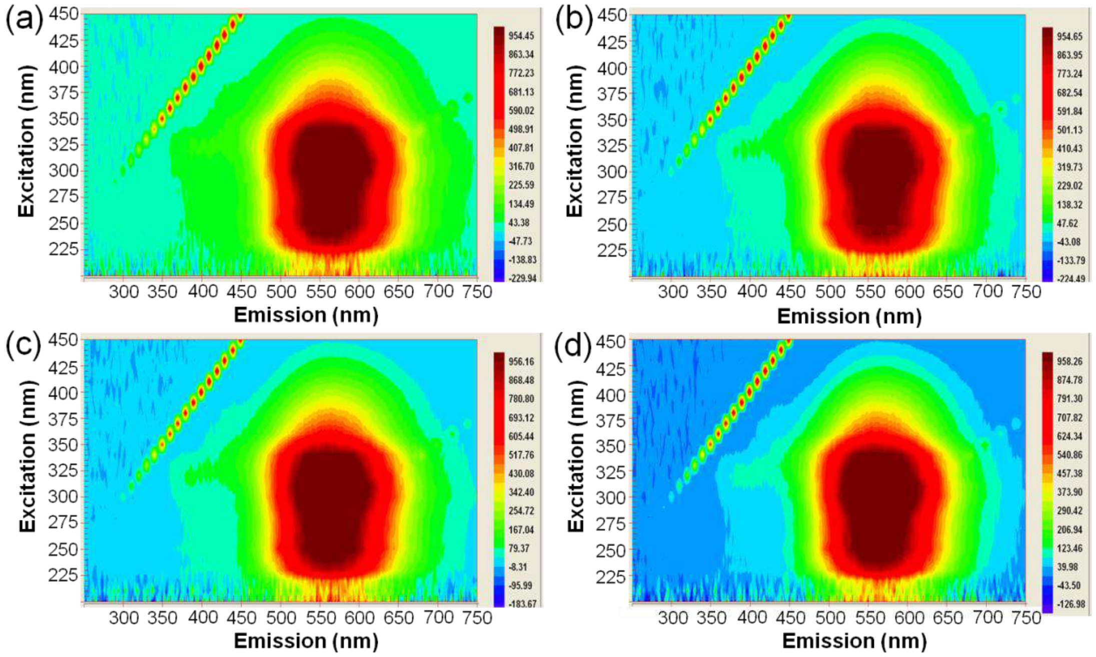

Supplementary Figure 77: Multi-wavelength (2D) fluorescence spectrum (excitation step width: 10 nm) of **G3** (c = 1.0 μM) in CH_3CN/methanol mixtures with different methanol contents (a) 0 %, (b) 30 %, (c) 60 %, (d) 90 %.

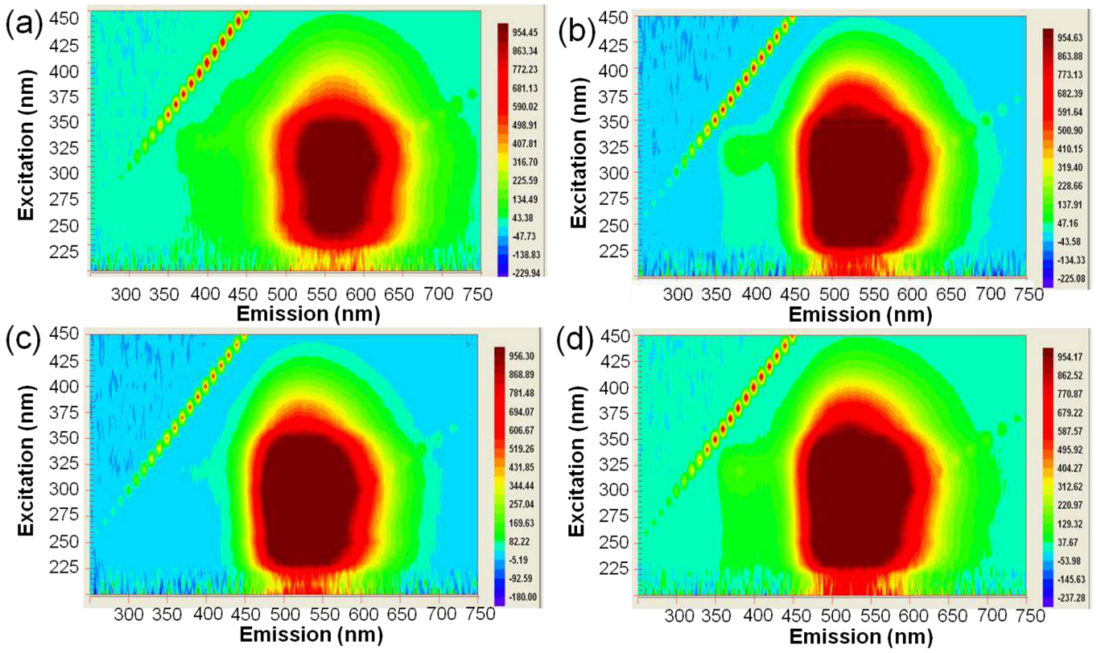

Supplementary Figure 78: Multi-wavelength (2D) fluorescence spectrum (excitation step width: 10 nm) of **G3** (c = 1.0 μM) in CH_3CN/H_2O mixtures with different water contents (a) 0 %, (b) 30 %, (c) 60 %, (d) 90 %.

Supplementary Table 2. CIE chromaticity coordinates of **G2** in CH_3CN/methanol mixtures with different fractions of methanol at 298 K (c = 1.0 μM).

Methanol fraction	CIE chromaticity coordinates
0 %	(0.280, 0.265)
10 %	(0.272, 0.252)
20 %	(0.268, 0.256)
30 %	(0.260, 0.237)
40 %	(0.255, 0.232)
50 %	(0.252, 0.230)
60 %	(0.254, 0.236)
70 %	(0.256, 0.238)
80 %	(0.264, 0.242)
90 %	(0.265, 0.244)

Supplementary Table 3. CIE chromaticity coordinates of **G2** in CH_3CN/H_2O mixtures with different fractions of H_2O at 298 K (c = 1.0 μM).

Water fraction	CIE chromaticity coordinates
0 %	(0.280, 0.265)
10 %	(0.220, 0.208)
20 %	(0.209, 0.196)
30 %	(0.212, 0.205)
40 %	(0.238, 0.233)
50 %	(0.265, 0.267)
60 %	(0.325, 0.355)
70 %	(0.390, 0.460)
80 %	(0.426, 0.525)
90 %	(0.430, 0.531)

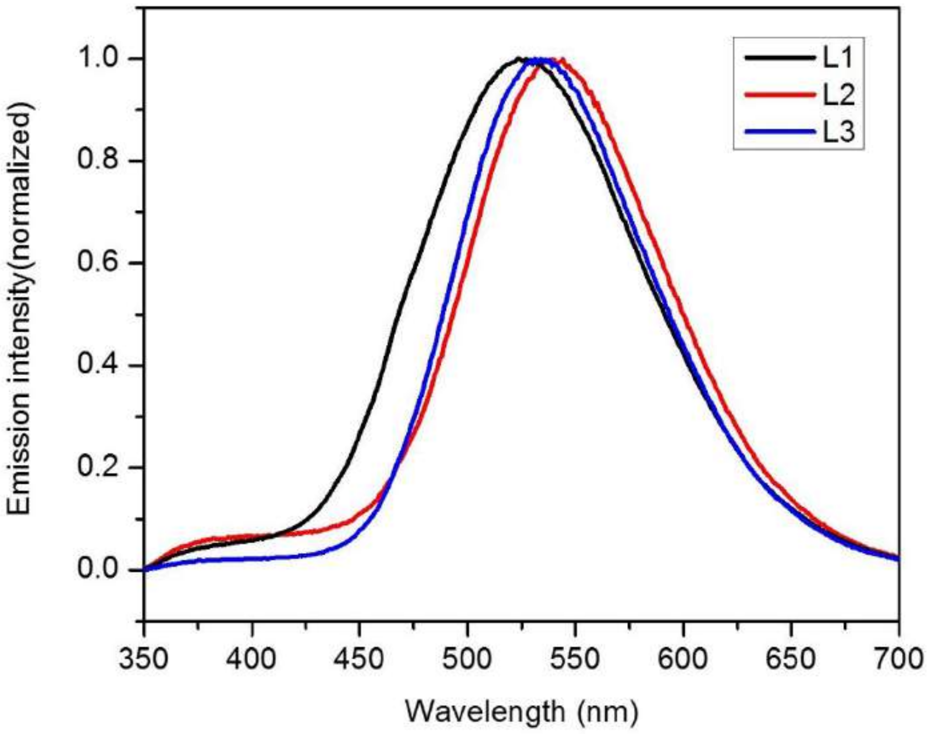

Supplementary Figure 79: Solid-state fluorescence spectra of ligands **L1**–**L3** (λ_{ex} = 320 nm).

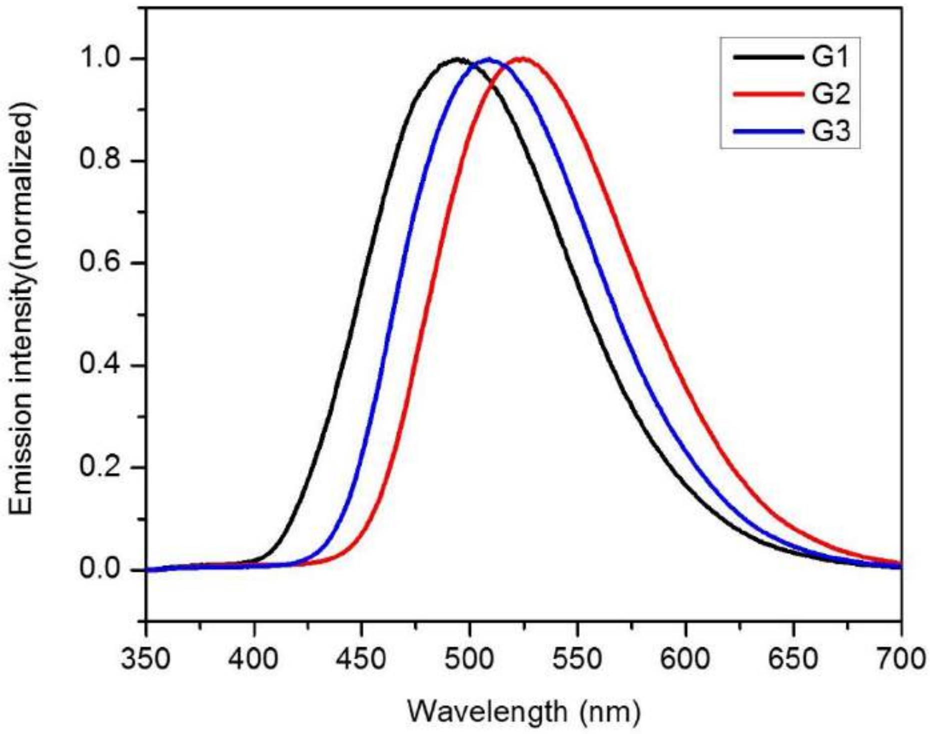

Supplementary Figure 80: Solid-state fluorescence spectra of supramolecular rosettes **G1**–**G3** (λ_{ex} = 320 nm).

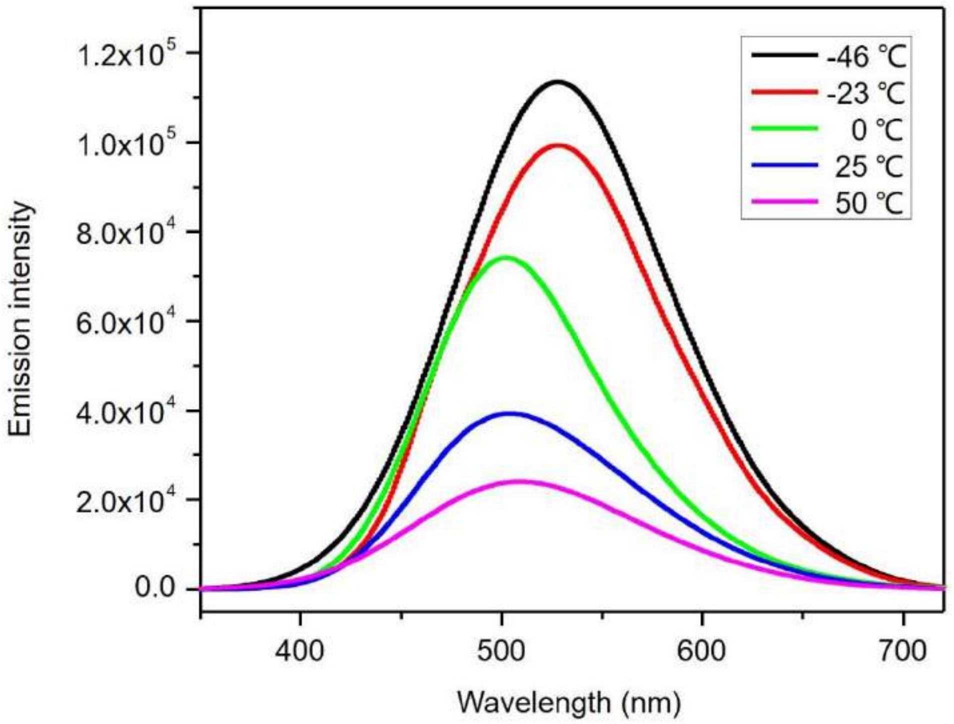

Supplementary Figure 81: Temperature dependence of fluorescence emission spectra of **G1** in CH_3CN (λ_{ex} = 320 nm, *c* = 1.0 μM)

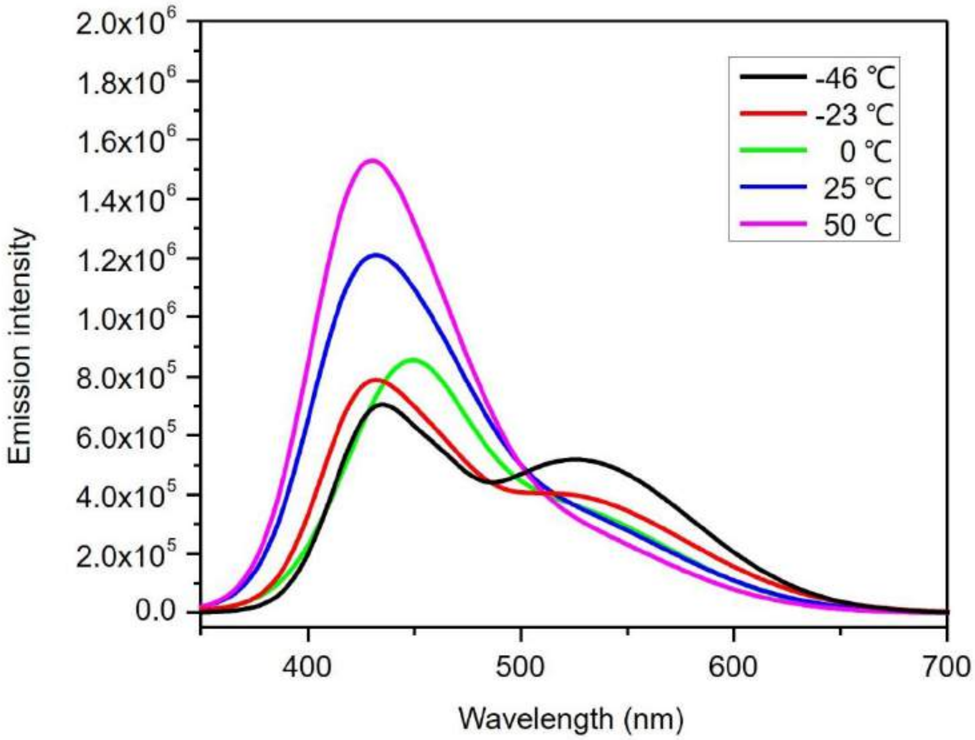

Supplementary Figure 82: Temperature dependence of fluorescence emission spectra of **G2** in CH_3CN (λ_{ex} = 320 nm, c = 1.0 μM).

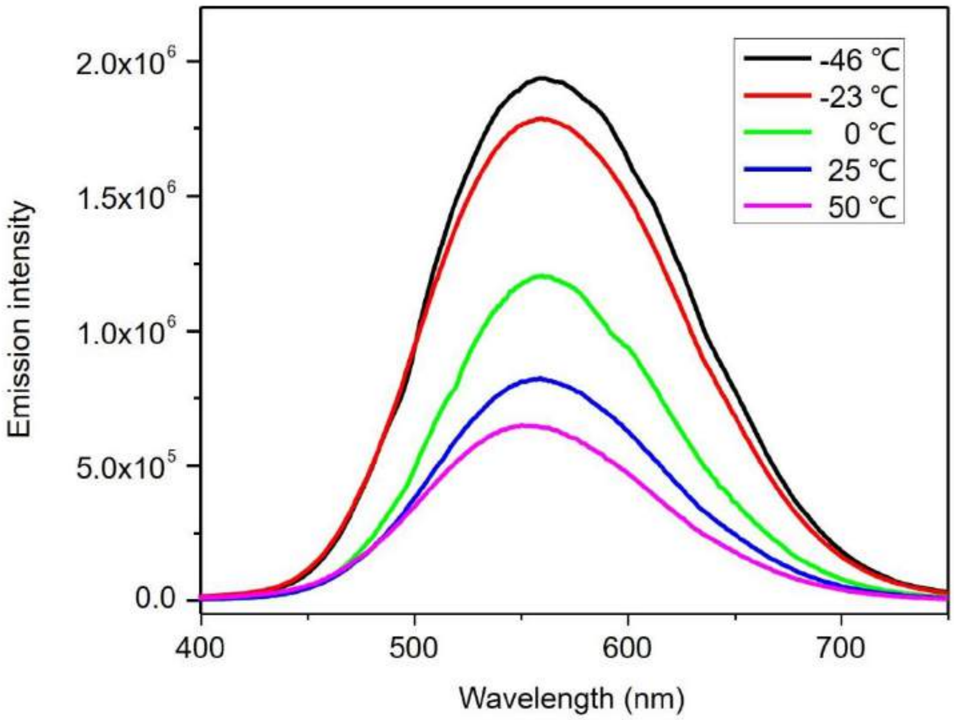

Supplementary Figure 83: Temperature dependence of fluorescence emission spectra of **G3** in CH_3CN (λ_{ex} = 320 nm, *c* = 1.0 μM).

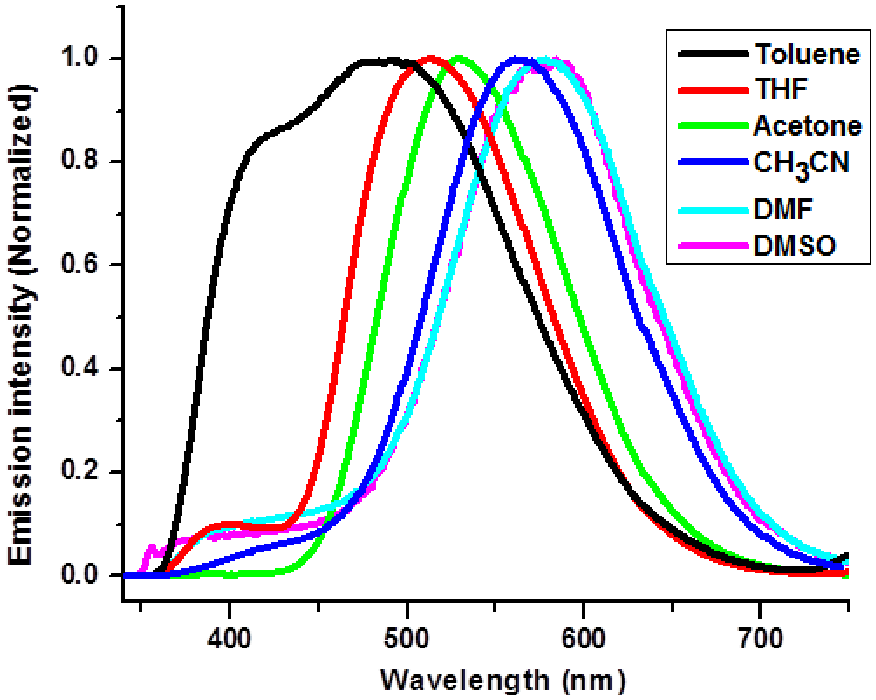

Supplementary Figure 84: The emission spectra of **G1** in different solvents (λ_{ex} = 320 nm, c = 1.0 μM)

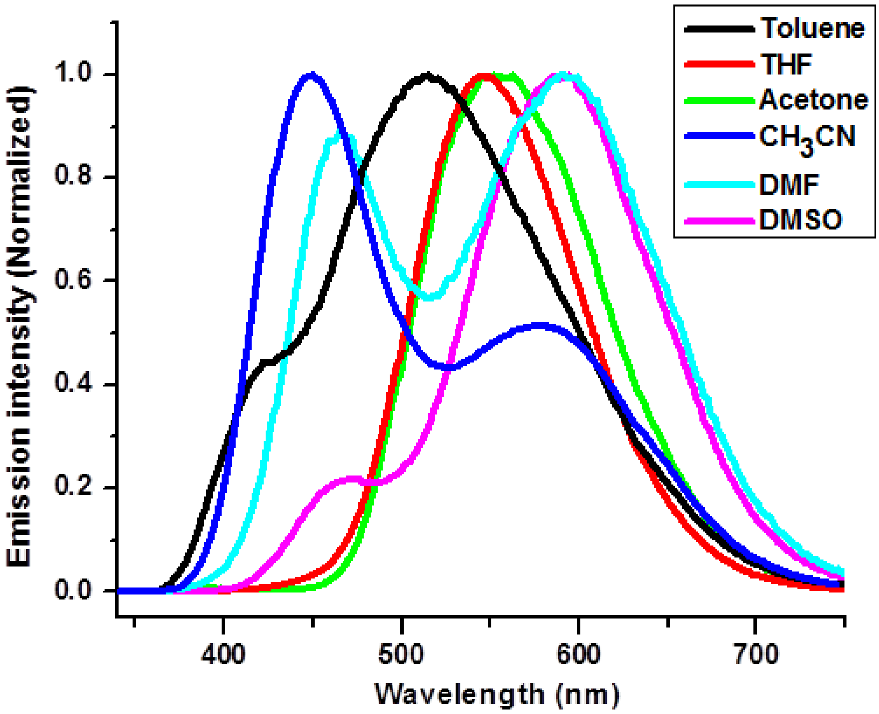

Supplementary Figure 85: The emission spectra of **G2** in different solvents (λ_{ex} = 320 nm, c = 1.0 μM)

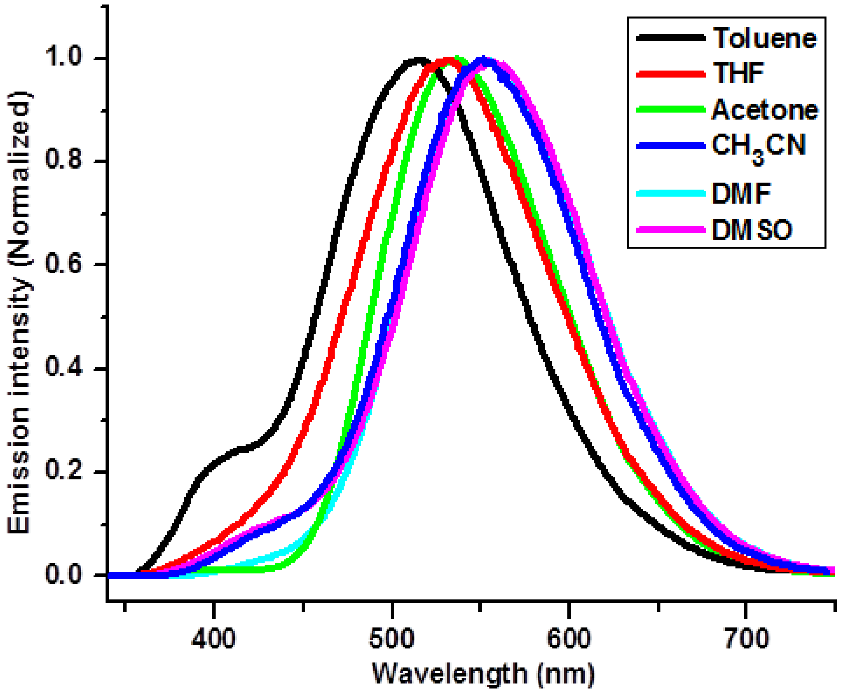

Supplementary Figure 86: The emission spectra of **G3** in different solvents (λ_{ex} = 320 nm, c = 1.0 μM)

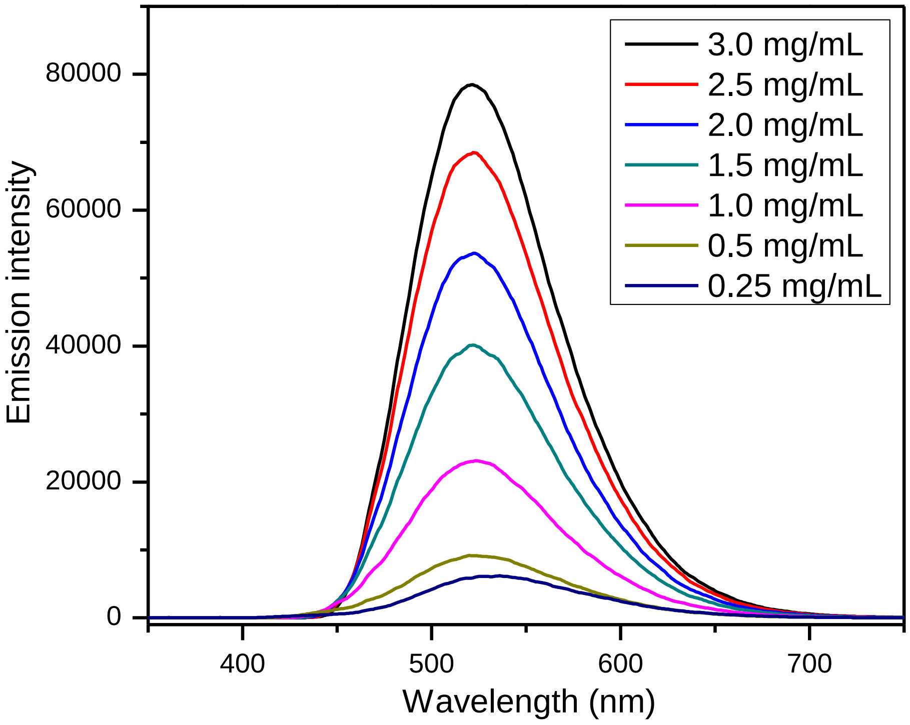

Supplementary Figure 87: The variant-concentration fluorescence of **G1** from 0.25 mg/mL to 3.0 mg/mL (λ_{ex} = 320 nm)

Time-resolved fluorescence decay profiles of G1–G3

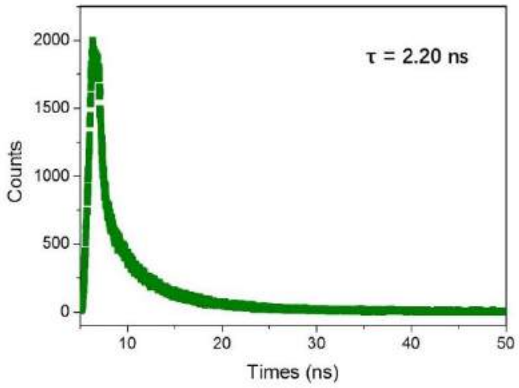

Supplementary Figure 88: Time-resolved fluorescence decay profiles of **G1** in CH_3CN (*c* = 1.0 μM).

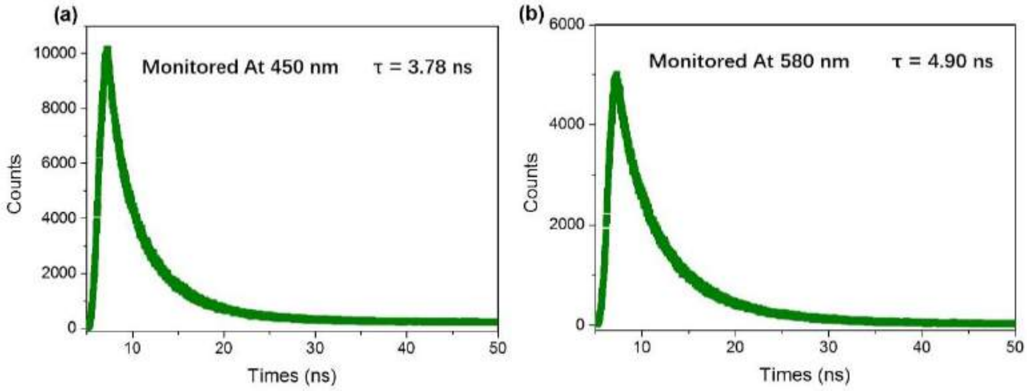

Supplementary Figure 89: Time-resolved fluorescence decay profiles of **G2** in CH_3CN (*c* = 1.0 μM) at the two wavelengths (a) at 450 nm, (b) at 580 nm.

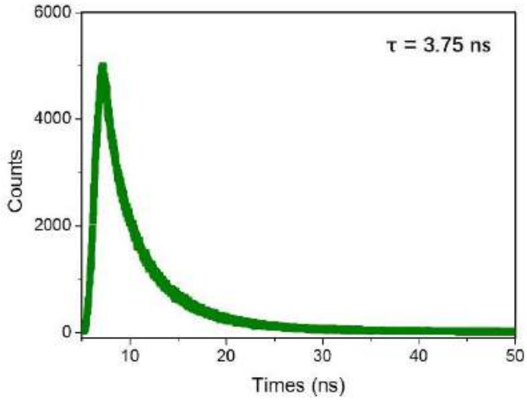

Supplementary Figure 90: Time-resolved fluorescence decay profiles of **G3** in CH_3CN (*c* = 1.0 μM).

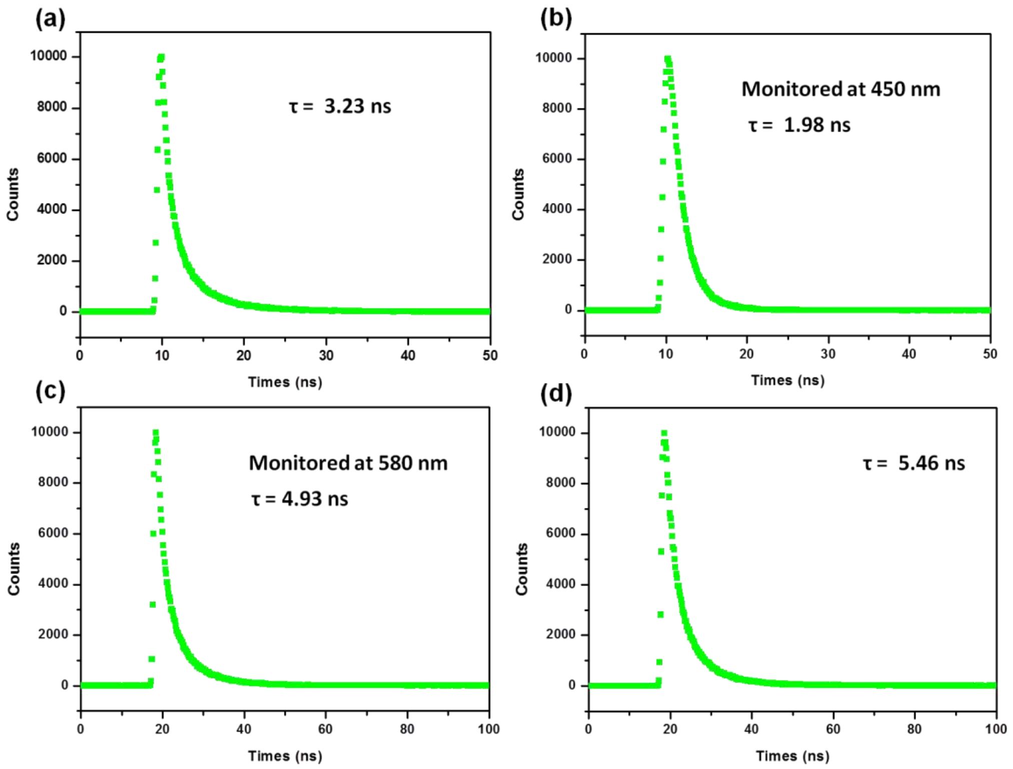

Supplementary Figure 91: Time-resolved fluorescence decay profiles of (a) **G1** and (d) **G3** in degassed CH_3CN (*c* = 1.0 μM). Time-resolved fluorescence decay profiles of **G2** in degassed CH_3CN (*c* = 1.0 μM) at the two wavelengths (b) at 450 nm, (c) at 580 nm.

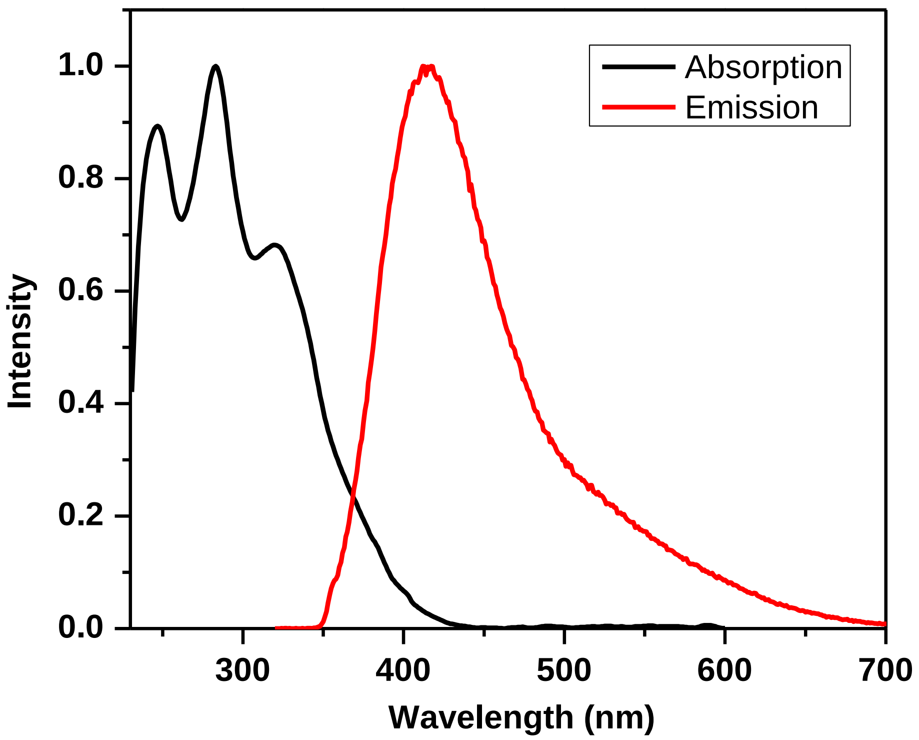

Supplementary Figure 92: Normalized absorption spectrum and fluorescence spectrum of **G1** in CH_3CN/H_2O mixtures with 10% water contents (λ_{ex} = 320 nm, c = 1.0 μM).

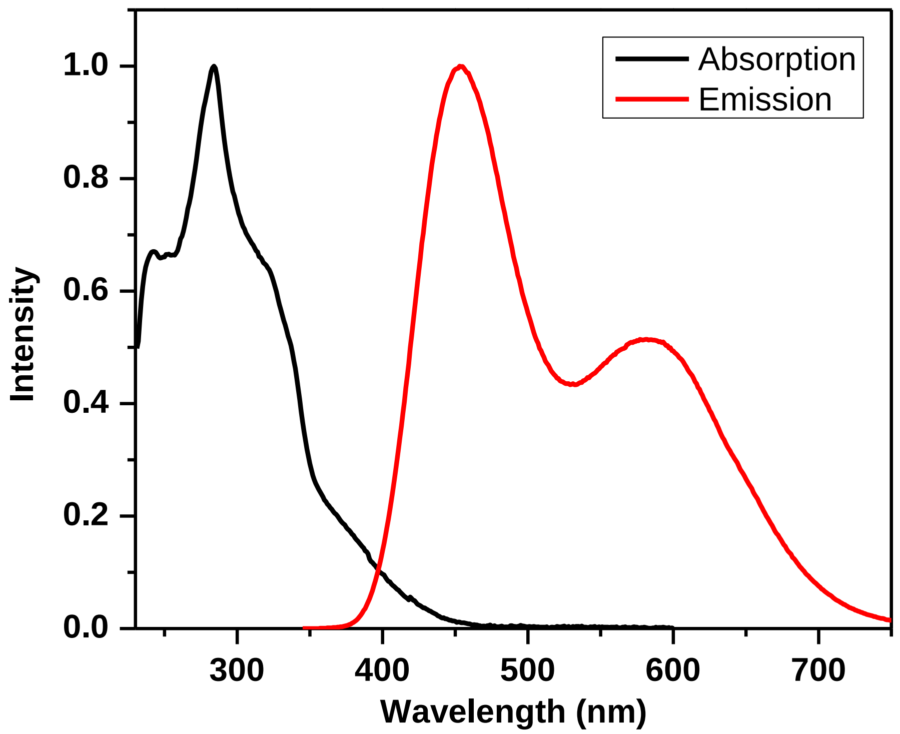

Supplementary Figure 93: Normalized absorption spectrum and fluorescence spectrum of **G2** in CH_3CN (λ_{ex} = 320 nm, c = 1.0 μM).

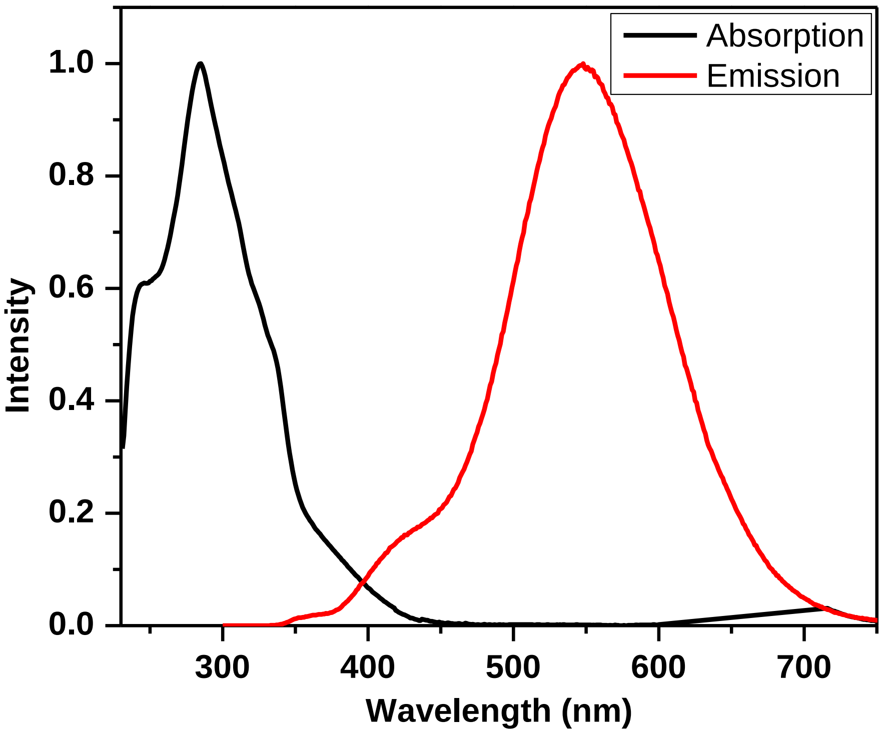

Supplementary Figure 94: Normalized absorption spectrum and fluorescence spectrum of **G3** in CH_3CN/MeOH mixtures with 90% methanol contents (λ_{ex} = 320 nm, c = 1.0 μM).

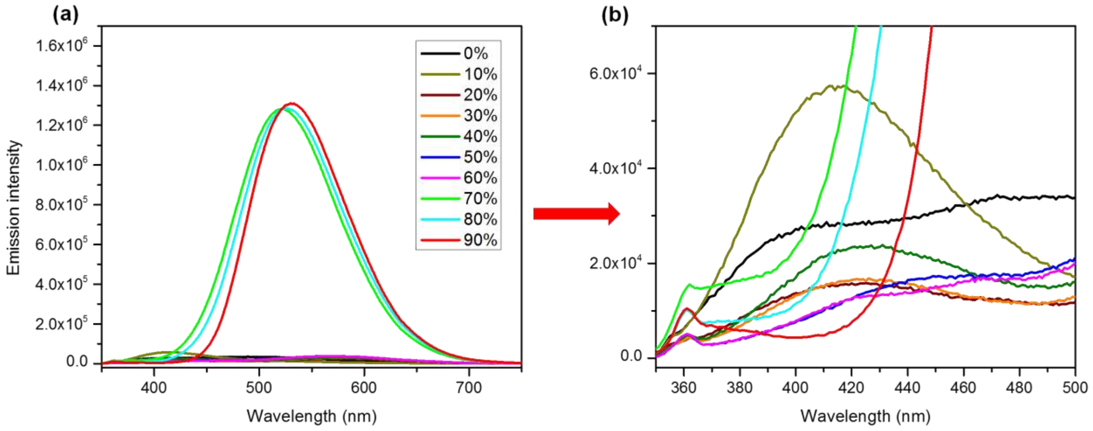

Supplementary Figure 95: (a) Fluorescence spectra of **G1** in CH_3CN/H_2O mixtures with different water contents (λ_{ex} = 320 nm, c = 1.0 μM), and (b) Zoom in 350 – 500 nm.

DLS data of the aggregates of G1–G3

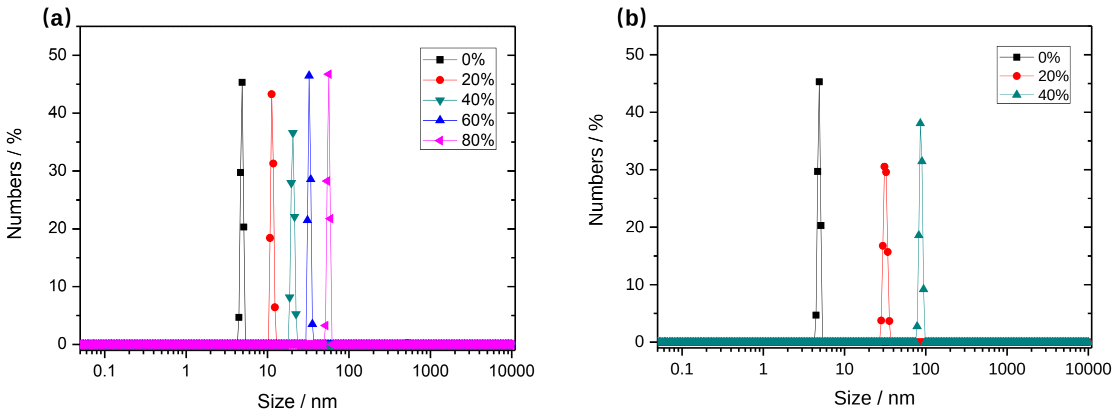

Supplementary Figure 96: Size distributions of **G1** (a) in CH_3CN/MeOH mixtures. The percentages in the graphs are the MeOH content. (b) in CH_3CN/H_2O mixtures. The percentages in the graphs are the H_2O content.

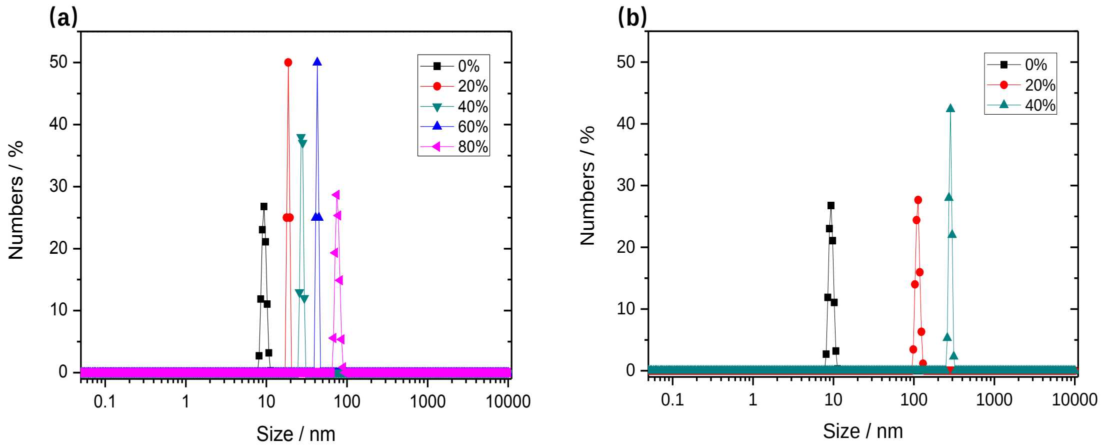

Supplementary Figure 97: Size distributions of **G2** (a) in CH_3CN/MeOH mixtures. The percentages in the graphs are the MeOH content. (b) in CH_3CN/H_2O mixtures. The percentages in the graphs are the H_2O content.

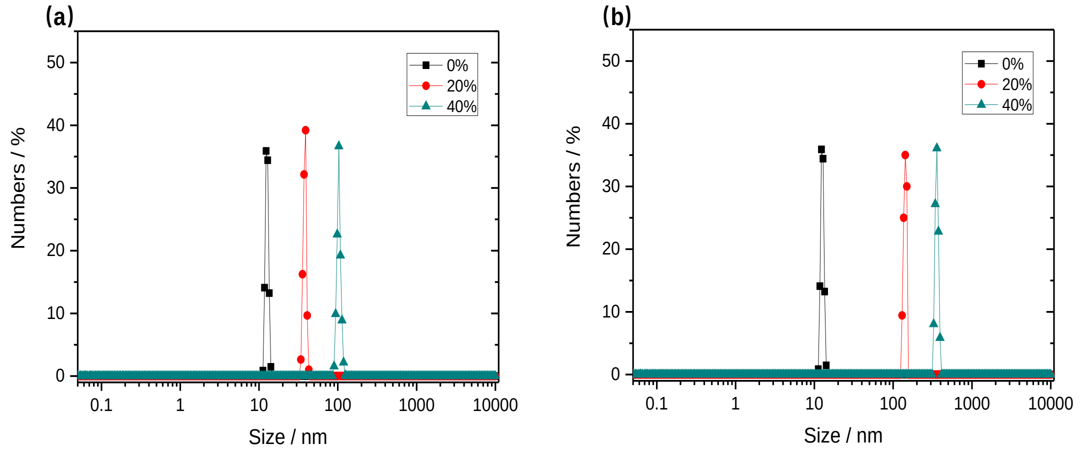

Supplementary Figure 98: Size distributions of **G3** (a) in CH_3CN/MeOH mixtures. The percentages in the graphs are the MeOH content. (b) in CH_3CN/H_2O mixtures. The percentages in the graphs are the H_2O content.

AFM statistical height histogram of G2 and G3

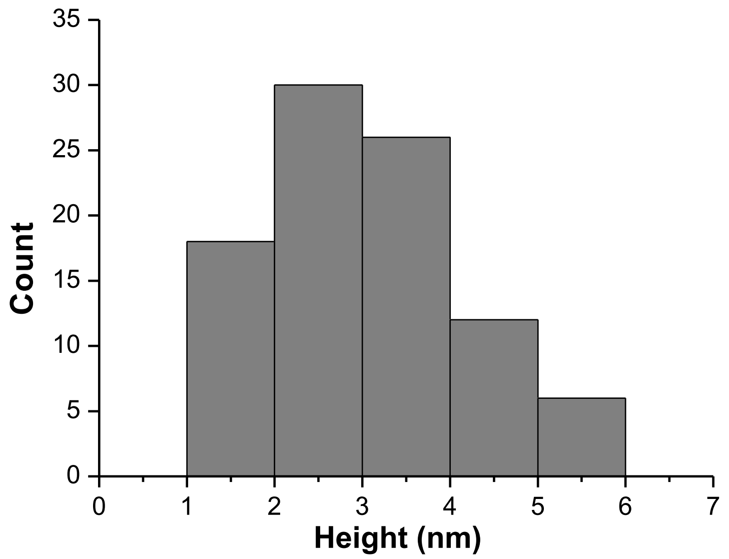

Supplementary Figure 99: Statistical height histogram of AFM for 75 dots of **G2**.

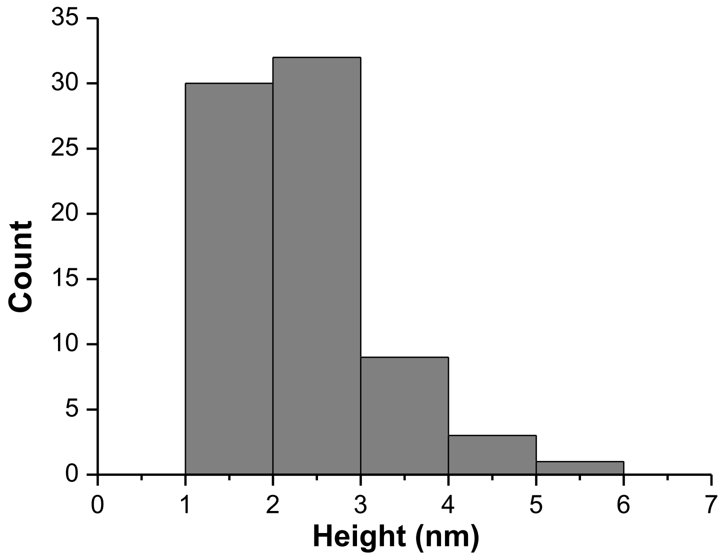

Supplementary Figure 100: AFM statistical height histogram for 90 dots of **G3**.

TEM images of nanotubes assembled by G2 and G3

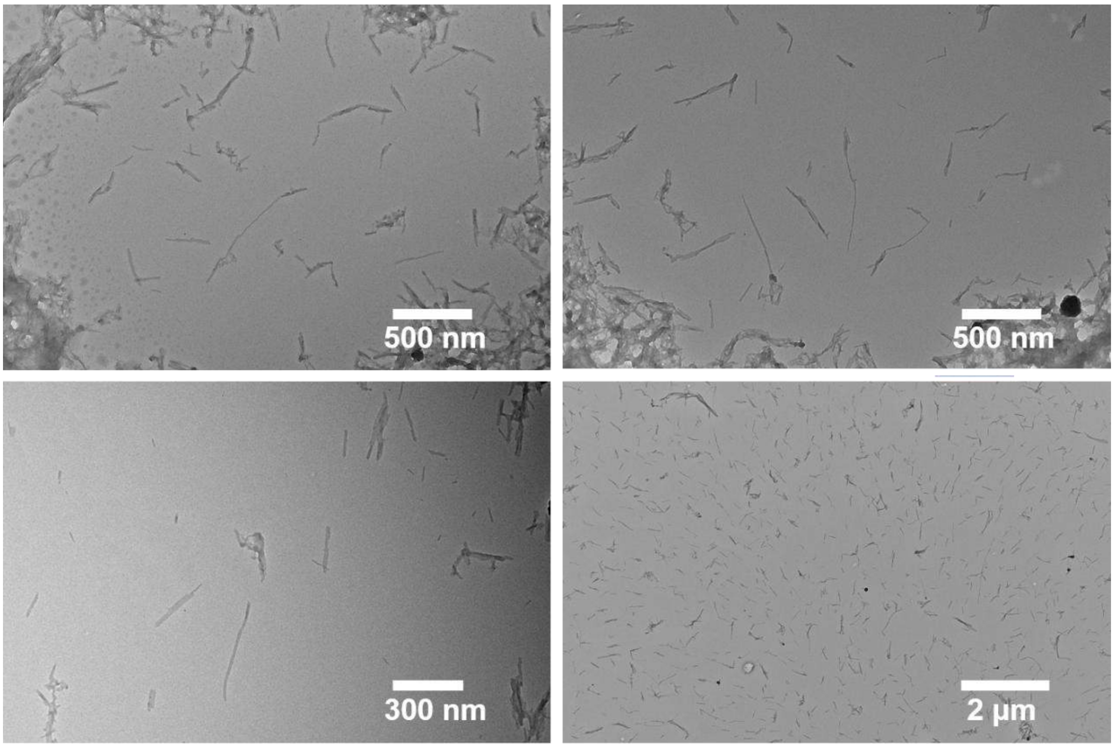

Supplementary Figure 101: TEM images of nanotubes assembled by **G2** in acetonitrile solution under isopropyl ether vapour.

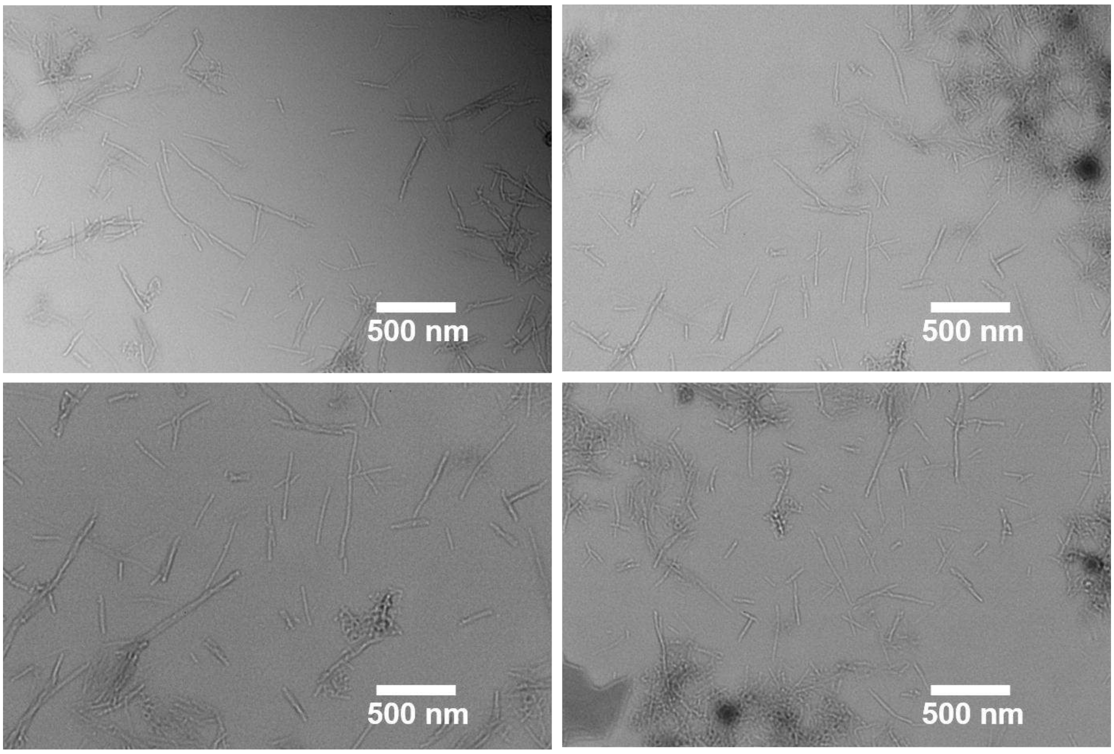

Supplementary Figure 102: TEM images of nanotubes assembled by **G3** in acetonitrile solution under isopropyl ether vapour.

TEM images of supramolecular rosettes G1–G3 in CH_3CN/methanol (or water) solutions with different methanol (or water) contents.

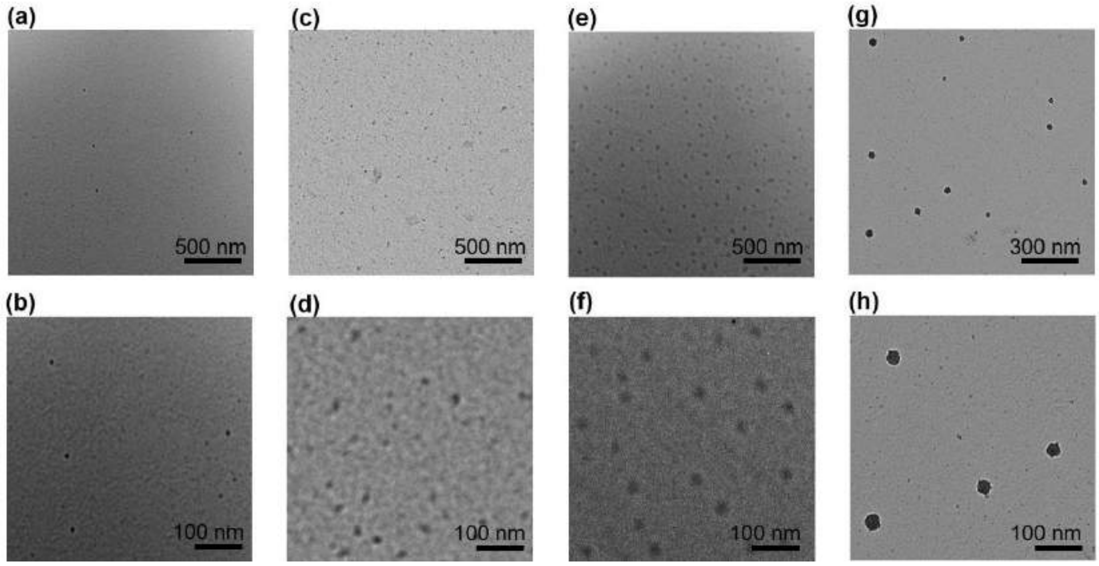

Supplementary Figure 103: TEM images of the aggregates of **G1** formed in the CH_3CN/methanol mixtures containing (a and b) 20%, (c and d) 40%, (e and f) 60%, and (g and h) 80% of methanol.

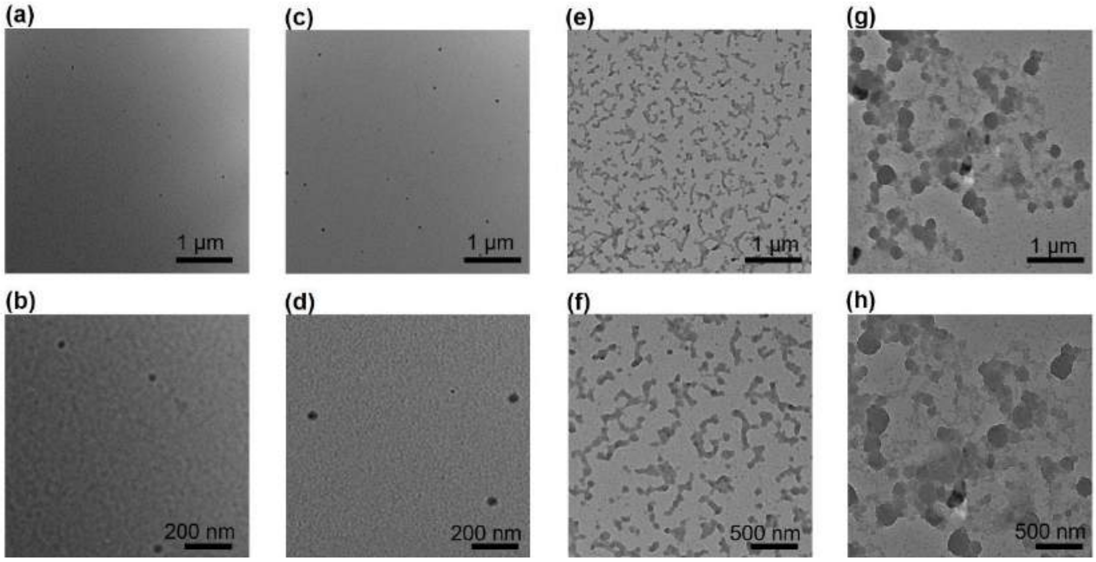

Supplementary Figure 104: TEM images of the aggregates of **G1** formed in the CH_3CN/water mixtures containing (a and b) 20%, (c and d) 40%, (e and f) 60%, and (g and h) 80% of water.

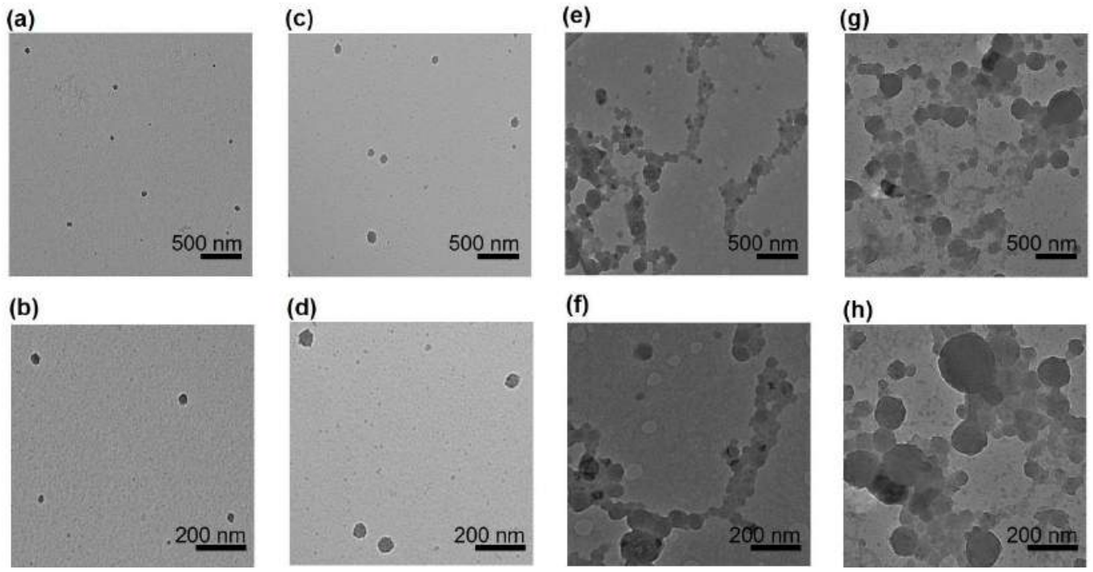

Supplementary Figure 105: TEM images of the aggregates of **G3** formed in the CH_3CN/methanol mixtures containing (a and b) 20%, (c and d) 0%, (e and f) 60%, and (g and h) 80% of methanol.

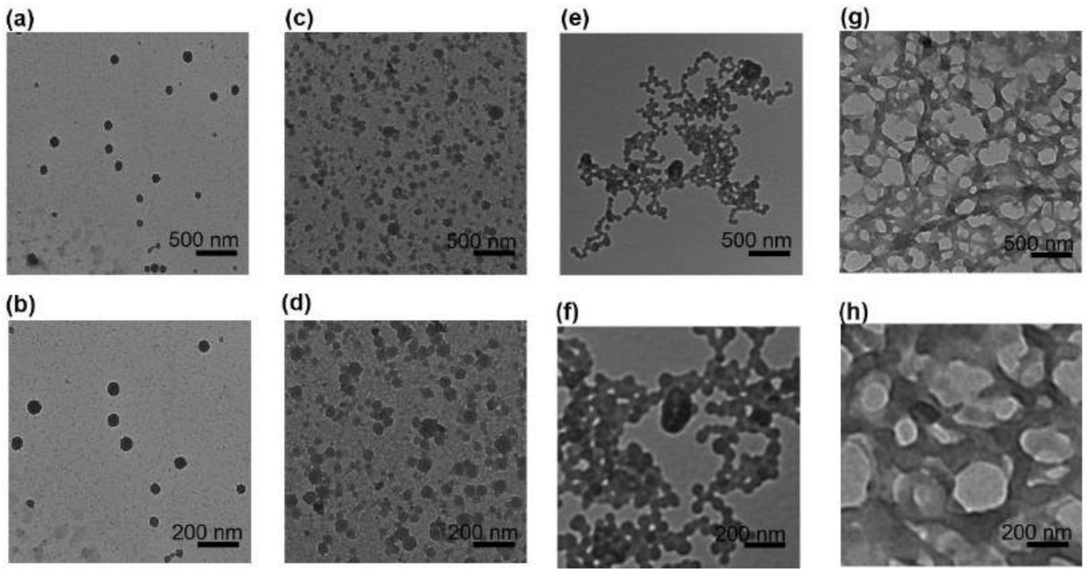

Supplementary Figure 106: TEM images of the aggregates of **G3** formed in the CH_3CN/water mixtures containing (a and b) 20%, (c and d) 40%, (e and f) 60%, and (g and h) 80% of water.

Supplementary References

1. Donovan, P. M. & Scott, L. T. Elaboration of Diaryl Ketones into Naphthalenes Fused on Two or Four Sides: A Naphthoannulation Procedure. *J. Am. Chem. Soc.* **126**, 3108-3112 (2004).

2. Jarosz, P., Lotito, K., Schneider, J., Kumaresan, D., Schmehl, R. & Eisenberg, R. Platinum(II) Terpyridyl-Acetylide Dyads and Triads with Nitrophenyl Acceptors via a Convenient Synthesis of a Boronated Phenylterpyridine. *Inorg. Chem.* **48**, 2420-2428 (2009).

3. Ni, S., Sha, W., Zhang, L., Xie, C., Mei, H., Han, J. & Pan, Y. N-Iodosuccinimide-Promoted Cascade Trifunctionalization of Alkynoates: Access to 1,1-Diiodoalkenes. *Organic Letters* **18**, 712-715 (2016).

4. Wang, M., Wang, C., Hao, X.-Q., Liu, J., Li, X., Xu, C., Lopez, A., Sun, L., Song, M.-P., Yang, H.-B. & Li, X. Hexagon Wreaths: Self-Assembly of Discrete Supramolecular Fractal Architectures Using Multitopic Terpyridine Ligands. *J. Am. Chem. Soc.* **136**, 6664-6671 (2014).

5. Rizzuto, F. J. & Nitschke, J. R. Stereochemical plasticity modulates cooperative binding in a $Co^{II}_{12}L_6$ cuboctahedron. *Nature Chemistry*, **9**, 903–908 (2017).

6. Bindfit v0.5 (Open Data Fit, 2016); http://app.supramolecular.org/bindfit/

7. Thordarson, P. Determining association constants from titration experiments in supramolecular chemistry. *Chem. Soc. Rev.* **40**, 1305-1323 (2011).

8. Shvartsburg, A. A. & Jarrold, M. F. An exact hard-spheres scattering model for the mobilities of polyatomic ions. *Chem. Phys. Lett.* **261**, 86-91 (1996).

9. Shvartsburg, A. A., Liu, B., Siu, K. W. M. & Ho, K.-M. Evaluation of Ionic Mobilities by Coupling the Scattering on Atoms and on Electron Density. *J. Phys. Chem. A.* **104**, 6152-6157 (2000).

10. Jarrold, M. F. Peptides and proteins in the vapor phase. *Ann. Rev. Phys. Chem.* **51**, 179-207 (2000).
